# General Index A-J

**Published:** 1834-06-01

**Authors:** 


### GENERAL INDEX.

| A | Vol. | Page |
|---|---|---|
| Abattement, explanation of | 20 | 98 |
| Abdomen, state of it, after ruptured uterus | 4 | 23 |
| Abdomen, pressure on | 4 | 521 |
| Abdomen, serious wounds of, cases | 4 | 547 |
| Abdomen, penetrating wounds of | 9 | 527 |
| Abdomen, knife found in the ! | 11 | 199 |
| Abdomen, morbid appearances of, in fever | 12 | 358 |
| Abdomen, chronic tumor in the | 15 | 498 |
| Abdomen, tubercular disease of | 15 | 466 |
| Abdomen, on exploration of | 16 | 446 |
| Abdomen, case of wound of | 17 | 226 |
| Abdomen, wounds of, B. Cooper on | 20 | 139 |
| Abdominal inflammation, opium in | 1 | 118 |
| Abdominal inflammation, case of | 3 | 528 |
| Abdominal tumour, 400 leeches applied ! | 4 | 201 |
| Abdominal injury, fatal case of | 6 | 472 |
| Abdominal tumours, some cases of | 7 | 169 |
| Abdominal inflammation, insidious, case of | 7 | 489 |
| Abdominal contusion, violent case | 8 | 506 |
| Abdominal aorta, cases of obliteration | 9 | 210 |
| Abdominal tumors, Dr. Seymour on | 9 | 399 |
| Abdominal fœtation, M. Rayer's case | 9 | 569 |
| Abdominal inflammation, Mr. Bates on | 12 | 313 |
| Abdominal inflammation, treatment of | 12 | 314 |
| Abdominal complication in fever | 12 | 351 |
| Abdominal aorta, aneurism of the | 14 | 174 |
| Abdominal pressure in obstetrics | 14 | 244 |
| Abdominal tumor, case of | 15 | 246 |
| Abdominal parietes, spontaneous perforation of | 17 | 441 |
| Abercrombie, Dr. on diseases of the heart | 1 | 72 |
| Abercrombie, Dr. on diseases of the brain | 8 | 337 |
| Abercrombie, Dr. on diseases of the brain | 9 | 73 |
| Abercrombie, Dr. on diseases of the stomach | 10 | 328 |
| Abercrombie, Dr. on diseases of the stomach, &c. | 11 | 1 |
| Abercrombie, Dr. on a peculiar delirium | 12 | 197 |
| Abercrombie on a peculiar affection of the pericranium | 12 | 256 |
| Abercrombie, Dr. on ramollissement of the cord | 12 | 487 |
| Abercrombie, Dr. on stricture of colon | 13 | 150 |
| Abercrombie, Dr. on the stomach and intestines | 13 | 223 |
| Abercrombie, Dr. on the intellectual powers | 14 | 289 |
| Aberdeen, regulations, &c. respecting degrees | 11 | 73 |
| Abernethian doctrines, criticisms on | 12 | 296 |
| Abernethy versus the Lancet | 3 | 297 |
| Abernethy, Mr. his optics all in the wrong | 6 | 256 |
| Abernethy, Mr. on spinal diseases | 6 | 263 |
| Abernethy, Mr. his lectures | 8 | 570 |
| Abernethy, Mr. on traumatic tetanus | 9 | 145 |
| Abernethy, Mr. on the liver | 9 | 154 |
| Abernethy on the morbid anatomy of the lungs | 9 | 272 |
| Abernethy, the late Mr. | 15 | 286 |
| Abernethy, the late Mr. | 15 | 563 |
| Abolition of the senses, melancholy case of | 9 | 440 |
| Abortion, artificial, its causes. | 1 | 341 |
| Abortion, Dr. Musgrave's case of | 2 | 513 |
| Abortion, culpable instance of | 2 | 528 |
| Abortion, the powers of medicines | 3 | 418 |
| Abortion, from compressing varicose veins | 4 | 245 |
| Abortion, Mad. Boivin on | 10 | 257 |
| Abortion produced by mercury | 10 | 472 |
| Abortion, criminal, review on | 13 | 97 |
| Abortion, has it been spontaneous or not ? | 13 | 103 |
| Abortion, English and French laws respecting | 13 | 108 |
| Abortion, Dr. Lee on | 16 | 446 |
| Abortion, Mr. Ingleby on | 17 | 342 |
| Abortion, Dr. Granville on | 19 | 130 |
| Abouzabel, Clot Bey's school at | 18 | 545 |
| Abouzabel, school at | 19 | 193 |
| Abraham, Mr. on the effects of civilization | 5 | 265 |
| Abscess in the stomach | 2 | 180 |
| Abscess of the lungs | 3 | 511 |
| Abscess of the liver, M. Louis on | 6 | 201 |
| Abscess of the liver, Dr. Graves's case of | 7 | 112 |
| Abscess of the liver; a misnomer | 7 | 522 |
| Abscess between the bladder & pubes, | 8 | 451 |
| Abscess, immense, in the abdomen | 9 | 195 |
| Abscess in the iliac region, cases of, | 9 | 274 |
| Abscess of the breast | 9 | 451 |

B

| | Vol. | Page |
|---|---|---|
| Abscess in the lungs after operations, cases of | 9 | 490 |
| Abscess in the substance of bone.... | 10 | 410 |
| Abscess of the liver, Mr. Annesley on | 10 | 314 |
| Abscess in the liver, Mr. Annesley on | 10 | 435 |
| Abscess in the cerebrum unsuspected, | 10 | 470 |
| Abscess, insidious, in front of the larynx and trachea | 10 | 575 |
| Abscess in the brain after fractured skull | 11 | 234 |
| Abscess around and in the kidney .. | 12 | 82 |
| Abscess, sloughy of the leg, report on | 13 | 281 |
| Abscess, fatal, from stricture | 14 | 160 |
| Abscess, encysted, of the spinal cord | 14 | 284 |
| Abscess, fatal, of the thigh | 14 | 516 |
| Abscess over the liver—curious case | 15 | 274 |
| Abscess of the pelvis after lithotomy | 15 | 367 |
| Abscess of the tonsil, best mode of opening | 15 | 384 |
| Abscess of the cheek, from bad teeth | 15 | 474 |
| Abscess of the brain | 16 | 255 |
| Abscess of the lung, Dr. Tweedie on, | 16 | 449 |
| Abscess external to the larynx, cases | 16 | 591 |
| Abscess of liver, cases of | 17 | 523 |
| Abscess of the tibia, Mr. Brodie on.. | 18 | 33 |
| Abscess in the heart, case of | 18 | 174 |
| Abscess in lung, opening externally.. | 18 | 182 |
| Abscess of the prostate | 18 | 445 |
| Abscess of the pelvis after lithotomy, | 18 | 571 |
| Abscess of the pelvis, cases of | 18 | 572 |
| Abscess of kidney | 19 | 159 |
| Abscess of the septum narium | 19 | 454 |
| Abscess of the cerebellum | 19 | 509 |
| Abscess, hepatic, Dr. Roots on | 19 | 540 |
| Abscess, pulmonary, obscure case of | 20 | 192 |
| Abscess of the liver, Mr. Geddes on, | 20 | 365 |
| Abscess of the mamma, Dr. Beatty on | 20 | 448 |
| Abscesses in the liver, unsuspected cases of | 7 | 278 |
| Abscesses opened by cupping-glass.. | 13 | 565 |
| Abscesses, fetid, near mucous membranes | 17 | 433 |
| Absence of the spinal marrow | 1 | 4 |
| Absorbents, effects of iodine on them | 4 | 6 |
| Absorbents, inflammation of, from poisoned wounds | 5 | 365 |
| Absorbents, nitrate of silver in inflammation of | 10 | 446 |
| Absorption, experiments on its nature | 5 | 321 |
| Absorption by skin, experiments on, | 18 | 22 |
| Abstinence in disease of heart | 15 | 491 |
| Abstinence in diseases of lungs | 15 | 492 |
| Abstinence in diseases of bowels | 15 | 492 |
| Abstinence, virtues of | 15 | 571 |
| Abstinence, Dr. Hall on | 16 | 450 |
| Abuse of purgatives in piles | 2 | 289 |
| Abuse of abstemious diet | 14 | 154 |
| Academy of minute anatomy | 6 | 630 |
| Academy of Medicine, France, proceedings at the | 18 | 543 |
| Academy of Sciences, proceedings of | 20 | 231 |
| Academy of Medicine, proceedings of | 20 | 235 |

| | Vol. | Page |
|---|---|---|
| Accidental colours, Sir I. Newton's case | 17 | 471 |
| Accoucheurs, unjust aspersions on.. | 3 | 175 |
| Account of the Penitentiary endemic | 3 | 1 |
| Accumulations in the colon, treatment of | 12 | 142 |
| Accumulations in the bowels, Mr. Annesley on | 13 | 214 |
| Acephalism, case of | 18 | 209 |
| Acephalocystes, remarks on | 18 | 544 |
| Acetas plumbi in pulmonary diseases | 20 | 193 |
| Acetate of quinine | 1 | 52 |
| Acetate of morphine in rheumatism. | 2 | 531 |
| Acetate of lead, its properties | 3 | 162 |
| Acetate of lead, its antidote | 3 | 163 |
| Acetate of lead, is it to be used? .. | 3 | 355 |
| Acetate of morphine externally, its good effects | 5 | 252 |
| Acetate of morphine and morphia .. | 12 | 545 |
| Acetate of morphia, Dr. Bardsley on | 13 | 333 |
| Acetum colchici, in hydrocephalic fever | 1 | 365 |
| Achor and acné, Dr. Todd on | 16 | 452 |
| Acid (lactic) in the human stomach.. | 2 | 145 |
| Acid nitrate of mercury, formula of, | 17 | 368 |
| Acid, nitric, effects of a large dose of | 20 | 64 |
| Acids and alkalies, influence of, on the blood | 13 | 218 |
| Acné, Mr. Plumbe on | 2 | 46 |
| Acné, cases of | 11 | 453 |
| Acné, M. Biett on | 16 | 396 |
| Acoustic surgery, Mr. Buchanan's illustrations of | 3 | 111 |
| Acoustic illusions, Sir D. Brewster on | 17 | 479 |
| Acoustics on heights and at great depths | 17 | 461 |
| Acrodynia, or Parisian epidemic | 14 | 200 |
| Action of medicines, Dr. Kinglake on | 1 | 225 |
| Action of the heart, Dr. Williams on the sounds of the | 12 | 155 |
| Actual cautery, employment of the . | 12 | 190 |
| Actual cautery, utility of | 17 | 164 |
| Actual cautery in vesico-vaginal fistula | 18 | 232 |
| Acupuncturation, Dr. Webster on .. | 3 | 562 |
| Acupuncturation, cases of | 11 | 166 |
| Acupuncture, culpable neglect in .. | 3 | 300 |
| Acupuncture, M. Pelletan on | 3 | 257 |
| Acupuncture, Mr. Bally's trials of .. | 3 | 260 |
| Acupuncture, effects of, in neuralgia | 5 | 621 |
| Acupuncture | 7 | 279 |
| Acupuncture, Dr. Elliotson on | 16 | 453 |
| Acute peritonitis | 1 | 200 |
| Acute tetanus, a fatal case of | 7 | 232 |
| Acute bronchitis, at the Val de Grace | 8 | 143 |
| Acute rheumatism, opiates in | 10 | 461 |
| Acute hepatitis—" morbid matters," | 11 | 167 |
| Acute rheumatism, quinine in | 13 | 437 |
| Acute rheumatism—pericarditis | 14 | 166 |
| Acute arteritis, supposed cases of .. | 15 | 219 |
| Adam, Dr. on hospital gangrene | 11 | 371 |
| Adams, Dr. on diseases of the heart, | 9 | 385 |

| | Vol. | Page |
|---|---|---|
| Adams, Dr. on tetanus | 13 | 476 |
| Adams, Mr. on congenital hernia | 18 | 486 |
| Addison, Mr. on the Malvern Waters | 10 | 24 |
| Addison, Dr. on poisons | 11 | 32 |
| Addison, Dr. on female disorders | 13 | 39 |
| Adherent hernia, criticisms on Mr. Stevens' views of | 11 | 546 |
| Adipocire, exostosis converted into! | 10 | 157 |
| Adipocire in a hydrocele | 11 | 533 |
| AdmiraltyOffice, new regulations from | 20 | 188 |
| Adulteration of wines | 4 | 313 |
| Adulterations of fecula | 20 | 516 |
| Adventitious structures, Dr. Hodgkin | 13 | 44 |
| Adynamic fever, Dr. Burne on | 9 | 1 |
| Adynamic fever, treatment of | 9 | 135 |
| Æstrus in the human subject | 19 | 527 |
| Æthmoid cells, polypus in the | 19 | 278 |
| Affection of legs, resembling elephantiasis | 11 | 272 |
| Affections, calculous, statistics of | 20 | 233 |
| Africa, Mr. Boyle on the diseases of, | 16 | 408 |
| African fever, cases of | 16 | 417 |
| Age of Libel | 10 | 266 |
| Age, Dr. Roget on | 16 | 453 |
| Agenesis cerebral, cases of | 7 | 420 |
| Agent immédiate du mouvement vital, M. Dutrochet on the | 11 | 49 |
| Ager, Dr. his circular | 6 | 283 |
| Aggregation of bone, thoughts on | 4 | 58 |
| Ague, interesting case of | 4 | 131 |
| Ague, Dr. Mackintosh on | 7 | 184 |
| Ague, partial, curious case of | 9 | 438 |
| Ague, masked double tertian, case of, | 11 | 215 |
| Ague, masked, curious case of | 11 | 465 |
| Ague, topical inflammations in | 12 | 307 |
| Ague treated by salicine | 20 | 482 |
| Aikin, Mr. on tartrite of iron and ammonia | 15 | 454 |
| Ainslie, Dr. on elephantiasis | 4 | 443 |
| Ainslie, Dr. his materia indica | 6 | 397 |
| Ainslie, Dr. on cholera | 16 | 439 |
| Air, fatal introduction of, by a vein | 2 | 218 |
| Air, admission of, into a vein | 9 | 522 |
| Air & exercise in phthisis pulmonalis | 12 | 241 |
| Air & pure water, action of, on lead, | 12 | 491 |
| Air, change of, in pursuit of health | 14 | 448 |
| Air, advantages of change of | 15 | 189 |
| Air, Dr. Clark on change of | 16 | 456 |
| Air, alterations of, from respiration, | 18 | 26 |
| Air in the circulating organs, accidents from | 18 | 517 |
| Air, fatal effects of admission into veins | 19 | 241 |
| Air, Dr. Graves on the secretion of. | 20 | 442 |
| Albers, Dr. on cholera in Moscow | 16 | 170 |
| Albuminous urine, Dr. Elliotson on | 14 | 513 |
| Alcock, Mr. on fractures of the patella and olecranon | 1 | 112 |
| Alcock, Mr. on the chlorurets of soda and lime | 6 | 349 |
| Alcock, Mr. his lectures on surgery | 13 | 24 |
| Alcock, Mr. death of | 19 | 571 |
| Aldersgate question | 20 | 185 |
| Alderson, Dr. on hooping-cough | 14 | 142 |
| Alexipharmacus' plan for improving the profession | 11 | 189 |
| Alibert, M. on papular and crustaceous psoriases | 7 | 189 |
| Alibert, Baron, on leuca of ancients | 12 | 523 |
| Alimentary canal, perforations of | 2 | 170 |
| Alimentary canal, hyperæmia of | 19 | 332 |
| Aliments, various, considered | 6 | 3 |
| Alison, Dr. on scrofula | 2 | 98 |
| Alison, Dr. his observations on sympathy | 6 | 63 |
| Alison, Dr. on tubercles | 10 | 72 |
| Alison, Dr. his outlines of physiology | 15 | 75 |
| Alison's, Dr. outlines of pathology | 19 | 306 |
| Allan, Mr. his contrivance for perforated soft palate | 3 | 548 |
| Allan, Mr. his case of spinal neuralgia | 10 | 244 |
| Allsop, Dr. death of | 17 | 574 |
| Aloes, Mr. Brande's account of | 3 | 145 |
| Aloes, Battley's watery extract of | 10 | 195 |
| Alopecia, Dr. Todd on | 16 | 456 |
| Alteratives, Dr. Conolly on | 16 | 457 |
| Alum, saturated solution of, as a styptic | 1 | 142 |
| Alum, as a styptic | 3 | 157 |
| Alum in diptheritis, M. Bretonneau on | 7 | 142 |
| Alveolar processes of both jaws removed; a case | 5 | 288 |
| Alveolar abscess | 11 | 414 |
| Amaurosis, sympathetic, with disordered bowels | 4 | 269 |
| Amaurosis, Dr. Belcher's case of | 4 | 572 |
| Amaurosis cured by long vomiting | 6 | 568 |
| Amaurosis, case of | 7 | 279 |
| Amaurosis, new mode of treating | 9 | 240 |
| Amaurosis | 9 | 496 |
| Amaurosis, Dr. Knox on | 13 | 206 |
| Amaurosis from debility, case of | 13 | 209 |
| Amaurosis from intemperance, case of | 13 | 209 |
| Amaurosis, treatment of | 13 | 211 |
| Amaurosis treated by strychnine | 13 | 441 |
| Amaurosis, Mr. Middlemore on | 13 | 460 |
| Amaurosis, strychnine in | 13 | 460 |
| Amaurosis, case of | 17 | 282 |
| Amaurosis from contusion | 18 | 579 |
| Amaurosis, Lisfranc's treatment of | 19 | 199 |
| Amaurosis from onanism | 19 | 214 |
| Amaurosis treated with strychnia | 19 | 260 |
| Amaurosis, Mr. Middlemore on | 19 | 447 |
| Amenorrhœa produced by mercury, | 10 | 471 |
| Amenorrhœa, Dr. Locock on | 16 | 458 |
| Amenorrhœa, causes of | 20 | 161 |
| Amenorrhœa, cured by sinapisms to the mammæ | 20 | 171 |
| America, cholera in | 20 | 179 |
| American indignation at English libellers | 12 | 455 |
| Amesbury, Mr. on non-union of fractures | 2 | 249 |
| Amesbury, Mr. on non-union of fracture | 8 | 524 |

| | Vol. | Page |
|---|---|---|
| Ammeline, Dr. his artificial preparations | 7 | 582 |
| Ammon, Dr. on restored vision | 19 | 156 |
| Ammonia in drunkenness | 1 | 229 |
| Ammoniacal pomade in cataract, its utility | 5 | 179 |
| Amnesia, or loss of language, case of, | 11 | 259 |
| Amphibious animals—Mr. Buffon's idea | 18 | 18 |
| Amputation of the lower jaw | 1 | 213 |
| Amputation for tetanus traumaticus | 1 | 217 |
| Amputation of the lower jaw | 2 | 210 |
| Amputation (successful) at hip-joint | 2 | 225 |
| Amputation, partial, of hand, case of | 3 | 267 |
| Amputation at the hip, Walther's case | 3 | 551 |
| Amputation by the flap | 4 | 237 |
| Amputation of the lower-jaw, Dr. Mc. Clellan's case | 5 | 532 |
| Amputation of a toe, death from | 5 | 578 |
| Amputation, during the progress of mortification | 7 | 219 |
| Amputation for gangrene | 7 | 238 |
| Amputation of finger followed by death | 7 | 264 |
| Amputation of part of the lower jaw | 7 | 289 |
| Amputation on the field & in hospital | 8 | 461 |
| Amputation, Mr. C. Bell on | 8 | 518 |
| Amputation during gangrene | 9 | 155 |
| Amputation at the hip-joint, successful case | 9 | 502 |
| Amputation of the neck of the uterus, | 9 | 562 |
| Amputation of cervix uteri, M. Lisfranc on | 10 | 159 |
| Amputation of the uterus | 10 | 215 |
| Amputation, Mr. Guthrie on | 10 | 233 |
| Amputation for fungus hæmatodes of the breast | 10 | 255 |
| Amputation, cases of | 10 | 477 |
| Amputation—abscess in the lungs | 11 | 162 |
| Amputation, primary, cases of | 11 | 279 |
| Amputation, secondary, cases of | 11 | 281 |
| Amputation for gangrenous ulcer | 11 | 379 |
| Amputation, partial of the foot | 15 | 124 |
| Amputation of shoulder-joint | 15 | 513 |
| Amputation at hip-joint | 15 | 513 |
| Amputation, fatal, for fungous tumor | 15 | 522 |
| Amputation, immediate, M. Larrey on | 16 | 60 |
| Amputation of fingers, fatal, cases of | 17 | 530 |
| Amputation of penis, case of | 18 | 223 |
| Amputation at the hip-joint | 19 | 195 |
| Amputation in a seven weeks' infant | 20 | 191 |
| Amputation of the leg by a ligature | 20 | 235 |
| Amputation in spreading gangrene | 20 | 265 |
| Amputations, &c. Mr. Hammick on | 12 | 405 |
| Amputations, illustrations of | 16 | 242 |
| Amussat, M. his treatment of varicocele | 11 | 256 |
| Amussat, M. his mode of stopping hæmorrhage | 11 | 545 |
| Analecta, or little periscope | 2 | 248 |
| Analecta Minora | 2 | 530 |
| Analecta Minora | 3 | 587 |
| Analogy between plague & yellow fever | 3 | 66 |
| Analogy between the effects of poison on man and brutes | 3 | 184 |
| Analysis of medical evidence, Dr. Smith's | 3 | 169 |
| Analysis, Dr. Todd's book of | 16 | 36 |
| Analysis of the Bombay reports on cholera | 17 | 65 |
| Anatomical discovery | 6 | 270 |
| Anatomical varieties, influence of, on operations | 9 | 199 |
| Anatomical preparations, new mode of preserving | 9 | 404 |
| Anatomical preparations, precautions respecting | 9 | 408 |
| Anatomical committee, report of the, | 9 | 543 |
| Anatomical preparations in wax | 13 | 192 |
| Anatomical catalogue, Dr. Hodgkin's | 13 | 367 |
| Anatomical engravings, Dr. Knox's | 14 | 245 |
| Anatomical plates, M. Schloss' | 14 | 566 |
| Anatomical models, Mr. Schloss's | 15 | 266 |
| Anatomical models, Mr. Schloss's | 17 | 564 |
| Anatomical plates, Mr. Schloss's | 18 | 261 |
| Anatomical atlas of Weber | 18 | 283 |
| Anatomical drawings in the Museum at Chatham | 20 | 304 |
| Anatomical plates recently published | 20 | 332 |
| Anatomical plates, Mr. Swan's | 20 | 334 |
| Anatomical plates, Mr. Quain's | 20 | 335 |
| Anatomie Vivante | 3 | 600 |
| Anatomie, Pathologique, by M. Cruveilhier | 12 | 79 |
| Anatomy of the ear, on the | 1 | 42 |
| Anatomy of the skin | 2 | 30 |
| Anatomy, Monro's Elements of | 4 | 52 |
| Anatomy of leeches described | 5 | 61 |
| Anatomy, petitions to Parliament on | 9 | 161 |
| Anatomy, Mr. Quain's elements of | 10 | 67 |
| Anatomy, Mr. Guthrie's letter on | 10 | 355 |
| Anatomy bill, Mr. Warburton's | 11 | 218 |
| Anatomy bill, remarks on the withdrawal of the | 11 | 285 |
| Anatomy and diseases of the teeth, Mr. Bell on | 11 | 328 |
| Anatomy, ravings of Gracchus on | 11 | 529 |
| Anatomy, general, Mr. Grainger's | 12 | 49 |
| Anatomy, lectures on, by Mr. B. Cooper | 12 | 95 |
| Anatomy, Dr. Thomson on the necessity of | 12 | 332 |
| Anatomy of a Chinese foot | 12 | 494 |
| Anatomy bill, Mr. Guthrie on the | 12 | 573 |
| Anatomy, difficulties of, in America | 13 | 499 |
| Anatomy, Bransby Cooper's lectures | 14 | 140 |
| Anatomy of the testis, Sir A. Cooper | 14 | 375 |
| Anatomy first taught in Alexandria | 15 | 398 |
| Anatomy in the 16th century | 15 | 407 |
| Anatomy, Massachusetts report on | 16 | 101 |
| Anatomy, lectures on | 16 | 239 |
| Anatomy, surgical, M. Velpeau's | 16 | 478 |
| Anatomy, pathological, of Otto | 17 | 427 |
| Anatomy, &c. of the ear, Mr. Tod's, | 17 | 431 |
| Anatomy, comparative, Carus's | 17 | 501 |

| | Vol. | Page |
|---|---|---|
| Anatomy Act | 17 | 566 |
| Anatomy, Mr. B. Cooper's lectures on | 18 | 97 |
| Anatomy, Mr. Quain's elements of | 18 | 242 |
| Anatomy, pathological, M. Cruveilhier on | 19 | 137 |
| Anatomy, pathological, Dr. Carswell on | 19 | 137 |
| Anatomy, pathological, M. Cruveilhier | 19 | 329 |
| Anatomy, morbid, by Dr. Hope | 19 | 329 |
| Anatomy, morbid, Dr. Hope on | 20 | 66 |
| Anatomy, morbid, Dr. Carswell on | 20 | 66 |
| Anatomy, morbid, Cruveilhier on | 20 | 66 |
| Anatomy of the liver, Mr. Kiernan's | 20 | 305 |
| Anatomy of the eye, Dr. Arnold on the | 20 | 490 |
| Anchylosis cured by a novel operation | 7 | 272 |
| Ancle, compound dislocation of the | 6 | 262 |
| Anderson, Dr. on tobacco in tetanus | 1 | 90 |
| Anderson, Dr. his amputation of the lower jaw | 10 | 567 |
| Anderson, Dr. on arsenic | 15 | 244 |
| Andes, thermal waters of the | 19 | 234 |
| Andral, Dr. on diseases of the bronchia | 1 | 451 |
| Andral, M. jun. on cicatrization of tubercles in the lungs | 5 | 214 |
| Andral, M. on chronic gastritis | 6 | 145 |
| Andral, M. his clinique medicale | 7 | 73 |
| Andral, M. on peritoneal inflammation | 8 | 66 |
| Andral, M. on phthisis | 12 | 97 |
| Andral, M. on cholera | 15 | 208 |
| Andral, M. on diseases of the liver | 15 | 85 |
| Andral, M. on the blood | 15 | 337 |
| Andral, M. on hyperæmia | 16 | 87 |
| Andral, M. on pericarditis | 19 | 49 |
| Andral's pathological anatomy | 14 | 434 |
| Andrews, Mr. on lunar caustic in stricture of the urethra | 7 | 69 |
| Andrew's, St. regulations respecting degrees | 11 | 74 |
| Andry, Dr. on gangrena senilis | 10 | 151 |
| Anecdote of Dr. Walker | 15 | 29 |
| Anemia, curious case of, by Dr. Coombe | 1 | 295 |
| Anencephalous monstrosity, case of, | 19 | 499 |
| Aneurism of the heart, depletion in | 3 | 101 |
| Aneurism of the heart in young people | 3 | 241 |
| Aneurism, aortic, case of | 4 | 200 |
| Aneurism cured by aneurism | 4 | 533 |
| Aneurism of aorta, Dr. Adam's case | 5 | 43 |
| Aneurism, Mr. Bell's case of | 5 | 574 |
| Aneurism from bleeding, cases of | 6 | 193 |
| Aneurism from venesection | 6 | 547 |
| Aneurism of the innominata and subclavian | 7 | 533 |
| Aneurism of the abdominal aorta | 7 | 233 |
| Aneurism of the carotid, supposed case of | 7 | 271 |
| Aneurism at the bend of the arm, Mr. White's case | 7 | 277 |
| Aneurism of the innominata, Mr. Wardrop's case | 7 | 534 |
| Aneurism, essential distinctions of | 8 | 153 |
| Aneurism—ligature *ultra aneurisma*, | 8 | 467 |
| Aneurism, false, of the heart, Dr. Elliotson | 8 | 174 |
| Aneurism of the temporal artery, cases of | 9 | 227 |
| Aneurism of the temporal artery | 9 | 469 |
| Aneurism of the aorta, bladder mistaken for | 10 | 152 |
| Aneurism of the meningea media | 10 | 203 |
| Aneurism and the new operation, Mr. Wardrop on | 11 | 46 |
| Aneurism, cure of, by pressure | 11 | 164 |
| Aneurism, false, of the heart; cases and remarks | 11 | 249 |
| Aneurism by anastomosis, ligature for | 11 | 444 |
| Aneurism, interesting cases of | 11 | 513 |
| Aneurism after venesection | 12 | 476 |
| Aneurism, remarks on | 12 | 502 |
| Aneurism, cases of | 13 | 276 |
| Aneurism, popliteal, cases of | 13 | 283 |
| Aneurism, the "dissecting" | 13 | 355 |
| Aneurism, true & false, description of | 13 | 357 |
| Aneurism, surgical treatment of | 13 | 365 |
| Aneurism, varicose, M. Dupuytren on | 13 | 509 |
| Aneurism by anastomosis | 13 | 565 |
| Aneurism of external iliac—aorta tied | 14 | 50 |
| Aneurism, common iliac tied | 14 | 54 |
| Aneurism, diffused, case of | 14 | 488 |
| Aneurism by anastomosis | 15 | 176 |
| Aneurism, iliac, successfully treated—cases | 16 | 514 |
| Aneurism, carotid, operation for | 16 | 520 |
| Aneurism, popliteal, operation for | 16 | 524 |
| Aneurism of the brachial artery | 17 | 204 |
| Aneurism, cases of, cured by compression | 17 | 204 |
| Aneurism, by anastomosis, case of, | 17 | 231 |
| Aneurism, flat ligatures in | 17 | 444 |
| Aneurism, ligature of subclavian for, | 18 | 247 |
| Aneurism of gluteal artery | 18 | 476 |
| Aneurism by anastomosis, cases of | 18 | 505 |
| Aneurism, carotid, case of | 19 | 448 |
| Aneurism, internal, cases of | 20 | 261 |
| Aneurism of the aorta, cases of | 20 | 262 |
| Aneurismal tumor, rather anomalous case of | 7 | 156 |
| Aneurismal pouch in the heart, M. Cruveilhier's case | 7 | 401 |
| Aneurismal dilatation of the posterior auris, &c. | 10 | 507 |
| Aneurismal tumor, deceptive | 13 | 569 |
| Aneurisms in the brain, M. Serres on the rupture of | 5 | 249 |
| Aneurisms, false, of brachial artery | 18 | 315 |
| Aneurisms, anastomosing, treatment of | 20 | 219 |
| Angina pectoris, Dr. Ryan on | 1 | 203 |
| Angina pectoris, Dr. Hosack on | 2 | 59 |
| Angina œdematosa, cases of | 3 | 205 |
| Angina pectoris, *mistaken* notion of its cause | 4 | 372 |
| Angina pectoris, marked case of | 4 | 497 |
| Angina maligna, muriatic acid *topically* in | 5 | 421 |

| | Vol. | Page |
|---|---|---|
| Angina maligna and croup, supposed identity of | 5 | 422 |
| Angina diptheritica, diagnosis of.... | 5 | 425 |
| Angina pectoris, supposed case of .. | 6 | 533 |
| Angina pectoris, M. Laennec on .... | 8 | 428 |
| Angina pectoris, Mr. Cooke on .... | 9 | 153 |
| Angina pectoris, pathology of ...... | 9 | 288 |
| Angina pectoris.................. | 10 | 196 |
| Angina pellicularis, M. Laennec on, | 10 | 466 |
| Angina pectoris—coronary arteries sound | 10 | 573 |
| Angina pellicularis .............. | 11 | 169 |
| Angina œdematosa, tracheotomy performed | 11 | 175 |
| Angina pectoris.. ................ | 12 | 126 |
| Angina pectoris, case of .......... | 15 | 216 |
| Angina pectoris, Dr. Forbes on .... | 16 | 459 |
| Angina externa, remarks on......... | 17 | 416 |
| Angina pectoris—diseased heart.... | 17 | 463 |
| Angina pectoris, Dr. Copland on, in diet | 18 | 112 |
| Angular projection of the spine .... | 2 | 141 |
| Angus-shire, leaping ague of ...... | 11 | 151 |
| Animal putrefaction, fatal instance of | 2 | 202 |
| Animal effluvia, their infectious powers | 3 | 67 |
| Animal heat and respiration, Dr. Williams on | 5 | 401 |
| Animal heat, Dr. Stevens' notions on | 17 | 325 |
| Animal heat, uniformity of........ | 18 | 23 |
| Animal electricity and muscular contractions | 18 | 211 |
| Animal magnetism, effects of...... | 19 | 184 |
| Animalculæ in the air, producing cholera | 16 | 182 |
| Animalculæ preceding cholera...... | 16 | 184 |
| Animalculæ in living blood ........ | 20 | 39 |
| Animalcular epidemic, in 1346 .... | 16 | 183 |
| Animals, vivisections of .......... | 9 | 103 |
| Animals, experiments on, in cases of poisoning | 12 | 373 |
| Animals, young, temperature of.... | 18 | 10 |
| Anne of Austria, sterile for 21 years | 20 | 159 |
| Annesley, Mr. on the diseases of India | 4 | 88 |
| Annesley, Mr. on effects of calomel | 4 | 328 |
| Annesley, Mr. his reports of Indian diseases | 5 | 342 |
| Annesley, Mr. on the diseases of India | 8 | 409 |
| Annesley, Mr. on the Diseases of India | 9 | 337 |
| Annesley, Mr. on the diseases of warm climates | 10 | 309 |
| Annesley, Mr. on hypochondriasis .. | 11 | 510 |
| Annesley, Mr. on accumulations in the colon | 12 | 138 |
| Annesley, Mr. on displacement of the colon | 12 | 570 |
| Annesley, Mr. on the diseases of India | 13 | 213 |
| Annesley, Mr. on dysentery........ | 15 | 102 |
| Anodynes, Dr. Whiting on ........ | 16 | 461 |
| Anomalies in eruptions .......... | 3 | 445 |
| Anomalous diseases of the spine .... | 1 | 313 |
| Anomalous gout, M. Bayle ........ | 2 | 187 |
| Anomalous disease of the testicle .. | 9 | 278 |
| Anomalous intermittents.......... | 9 | 293 |
| Anomalous pain in the head, curious case of | 10 | 172 |
| *Anonymous* school of medicine, alias the *idiopathists*, their errors, &c. | 5 | 226 |
| Antacids, *specific* in cholera morbus | 4 | 259 |
| Anterior tibial artery, wound of the, | 9 | 198 |
| Anterior cerebral lobes, congenital abscess of | 14 | 195 |
| Anterior membrane of the eyeball .. | 20 | 446 |
| Anthelmintic emulsion............ | 17 | 201 |
| Anthelmintics, Dr. Thomson on .... | 16 | 462 |
| Anti-contagionists, a *damper* for them | 3 | 292 |
| Anticyra, the mad-house of the Greeks | 13 | 303 |
| Antidotes ...................... | 3 | 589 |
| Anti-inebriants, basis of the ...... | 10 | 194 |
| Antimonial powder, inert ........ | 1 | 227 |
| Antimonial plaister, recipe for...... | 5 | 291 |
| Antimonium tartarizatum, remarks on | 2 | 357 |
| Antimony, in large doses, in pneumonia | 1 | 221 |
| Antimony, tartrite of, externally applied | 11 | 495 |
| Antimony, large doses of, in phlebitis, | 19 | 486 |
| Antimony, use of, in cholera ...... | 20 | 145 |
| Antiphlogistic treatment of gonorrhœa | 20 | 276 |
| Antiquity of the Cæsarian section .. | 4 | 474 |
| Antrum, cancer of the ............ | 11 | 400 |
| Antrum maxillare, disease of the.... | 11 | 415 |
| Antwerp, surgical account of siege of | 18 | 578 |
| Anus, leeches to the; remarks on the practice | 5 | 53 |
| Anus, imperforate, case of ........ | 10 | 515 |
| Anus, prolapsed, treated in Mr. Hey's manner | 12 | 154 |
| Anus and rectum, Dr. Colles on.... | 14 | 218 |
| Anus, fissure of, cured by belladonna, | 18 | 187 |
| Anus, fissure of.................. | 19 | 542 |
| Aorta, obliteration of the.......... | 6 | 481 |
| Aorta, obliteration of the.......... | 7 | 426 |
| Aorta, aneurism of the............ | 13 | 179 |
| Aorta, aneurisms of the .......... | 13 | 187 |
| Aorta, aneurism of the............ | 13 | 277 |
| Aorta, compression of, in uterine hæmorrhage | 14 | 152 |
| Aorta, rupture of semilunar valves .. | 14 | 197 |
| Aorta, extensive disease of the...... | 15 | 449 |
| Aorta, pressure on, in uterine hæmorrhage | 17 | 358 |
| Aorta, anomaly of the arch of...... | 19 | 239 |
| Aortic aneurisms, case of.......... | 8 | 559 |
| Aortic aneurisms, treatment of .... | 13 | 280 |
| Aortic aneurism, bursting into the œsophagus | 15 | 508 |
| Aortic aneurism, Dr. Hope on...... | 16 | 463 |
| Apennines, scenery of ............ | 14 | 454 |
| Apertures of the pelvis, dimensions of | 17 | 28 |
| Aphonia, periodical, a case of...... | 5 | 525 |
| Aphonia, intermittent, cases of .... | 11 | 488 |
| Aphonia and idiotcy from fright.... | 13 | 512 |

| | Vol. | Page |
|---|---|---|
| Aphonia—aphthæ, Dr. Robertson on | 16 | 466 |
| Aphonia treated by argenti nitras .. | 17 | 435 |
| Aphonia, Dr. Webster on.......... | 18 | 551 |
| Aphorism of Galen .............. | 18 | 514 |
| Apoplectiform disease, curious case of | 20 | 164 |
| Apoplexy, meningeal, Dr. Johnson on | 1 | 204 |
| Apoplexy, tendency of puerperal women to .................... | 2 | 517 |
| Apoplexy, its severe forms ........ | 4 | 366 |
| Apoplexy, sanguineous, a most astounding case .............. | 4 | 267 |
| Apoplexy, history and pathology of | 5 | 415 |
| Apoplexy, several forms of ........ | 9 | 75 |
| Apoplexy, serous and sanguineous .. | 9 | 76 |
| Apoplexy, treatment of .......... | 9 | 83 |
| Apoplexy thought to be caused by gymnastics ................. | 10 | 211 |
| Apoplexy of the lungs ............ | 10 | 514 |
| Apoplexy, chronic inflammation of the cerebral vessels .......... | 10 | 585 |
| Apoplexy, hypertrophy of the heart in | 11 | 458 |
| Apoplexy, interesting cases of...... | 12 | 204 |
| Apoplexy simulated by gastric irritation..................... | 12 | 251 |
| Apoplexy, interesting cases of...... | 12 | 457 |
| Apoplexy of lungs, curious cases of.. | 12 | 555 |
| Apoplexy of spleen, Mr. Ancill's case | 13 | 247 |
| Apoplexy, from ruptured middle cerebral artery .... ..... .. | 14 | 234 |
| Apoplexy of the lungs, two cases of.. | 14 | 360 |
| Apoplexy, Dr. Bright on.......... | 16 | 12 |
| Apoplexy of the spinal cord ...... | 16 | 19 |
| Apoplexy, blood-letting in ........ | 16 | 19 |
| Apoplexy, diuretics in ............ | 16 | 21 |
| Apoplexy, serous and nervous...... | 16 | 228 |
| Apoplexy, Dr. Clutterbuck on...... | 16 | 467 |
| Apoplexy, pulmonary, Dr. Townsend, | 16 | 470 |
| Apoplexy, M. Dance on a species of, | 17 | 186 |
| Apoplexy, case of................ | 17 | 187 |
| Apoplexy of uterus .............. | 17 | 319 |
| Apoplexy of new-born children .... | 19 | 138 |
| Apoplexy, French opinions on ..... | 19 | 504 |
| Apoplexy of the muscles .......... | 19 | 512 |
| Apoplexy, Dr. Uwins on .......... | 20 | 57 |
| Apothecaries' Transactions reviewed | 1 | 112 |
| Apothecaries' Act, curious trial at York | 1 | 243 |
| Apothecaries' Act, remarks on...... | 1 | 505 |
| Apothecaries, report of .......... | 3 | 599 |
| Apothecaries' Hall, regulations of the | 8 | 242 |
| Apothecaries' Company, new curriculum of the ............. | 10 | 189 |
| Apothecaries' Company, Irish, injustice of the............... | 11 | 196 |
| Apothecaries' Company, regulations of | 12 | 169 |
| Apothecaries' Company .......... | 13 | 574 |
| Apothecaries' Company, answers to queries ...... .......... | 14 | 256 |
| Apothecaries' Company's rules for lectures.................. | 15 | 145 |
| Apothecaries, and their usurpations, | 19 | 286 |
| Appendix to Parry's Elements of Pathology ................. | 3 | 188 |

| | Vol. | Page |
|---|---|---|
| Appendix to the papers on the nerves, by Mr. Bell .......... | 6 | 412 |
| Apprenticeship system, injustice of.. | 12 | 446 |
| Apyrectic diseases, Prof. Fulci on .. | 7 | 225 |
| Aquo-capsulitis, Mr. Mackenzie on.. | 15 | 69 |
| Arabian physicians .............. | 15 | 402 |
| Arachnitis spinalis .............. | 1 | 30 |
| Arachnitis cured ................ | 1 | 458 |
| Arachnitis, case of .............. | 4 | 210 |
| Arachnitis, with acute gastritis .... | 13 | 158 |
| Arachnitis, supposed cases of ...... | 13 | 169 |
| Arachnoid membrane, curious condition of the ................ | 11 | 187 |
| Arbitrary association of ideas ...... | 14 | 300 |
| Argenti nitras, Dr. Hood on the.... | 11 | 211 |
| Argenti nitras, mode of applying on a probe.................. | 18 | 187 |
| Argentum nitratum, ointment of, in eye-diseases .............. | 9 | 569 |
| Arm and shoulder presentations, Dr. Lee on.................. | 8 | 470 |
| Armies, mortality in.... .......... | 20 | 231 |
| Armstrong, Dr. on opium in inflammation ................ | 1 | 118 |
| Armstrong, Dr. his morbid anatomy, | 10 | 118 |
| Armstrong, Dr. on the morbid anatomy of the bowels, &c. ...... | 11 | 97 |
| Armstrong, Dr. biographical sketch of | 12 | 281 |
| Armstrong, Dr. the late .......... | 14 | 242 |
| Armstrong, Dr. life of ............ | 18 | 374 |
| Armstrong, Dr. his start in London, | 18 | 376 |
| Armstrong, Dr. his rejection ...... | 18 | 378 |
| Armstrong, Dr. his malady ........ | 18 | 379 |
| Armstrong, Dr. his post-mortem examination ................ | 18 | 382 |
| Armstrong, Dr. his character ...... | 18 | 383 |
| Armstrong's, Dr. letter to the College | 4 | 273 |
| Armstrong's morbid anatomy of the bowels.................. | 9 | 347 |
| Army medical fund, celebration of.. | 1 | 252 |
| Army and navy practitioners, their general merits ............ | 5 | 210 |
| Army Medical Board, regulations of | 6 | 291 |
| Arnott, Mr. on ptyalism .......... | 2 | 153 |
| Arnott, Dr. his elements of physics.. | 7 | 35 |
| Arnott, Mr. on the contagious character of erysipelas .......... | 7 | 148 |
| Arnott, Dr. reply of Dr. Barry to .. | 7 | 436 |
| Arnott, Mr. on inflammation of veins | 12 | 1 |
| Arnott, Dr. his elements of physics. | 12 | 334 |
| Arnott, Mr. letters to and from .... | 17 | 503 |
| Arnott, Mr. case of œsophagotomy.. | 20 | 172 |
| Arnott's, Dr. hydrostatic bed ...... | 17 | 564 |
| Arracan, its medical topography.... | 11 | 318 |
| Arsenic, Dr. Paris on ............ | 1 | 319 |
| Arsenic, various tests for.... .... | 1 | 321 |
| Arsenic, mixed with dumplings, Fenning's case .............. | 3 | 179 |
| Arsenic does *not* prevent the *rising* of dough ................ | 3 | 181 |
| Arsenic does *not* blacken knives .... | 3 | 182 |
| Arsenic, case of poisoning by ...... | 3 | 289 |

| | Vol. | Page |
|---|---|---|
| Arsenic in cholera, very large doses of | 7 | 164 |
| Arsenic and laudanum, poisoning by | 7 | 170 |
| Arsenic, Dr. Christison on | 7 | 379 |
| Arsenic, apparently fatal effects of | 10 | 170 |
| Arsenic, on its therapeutic properties | 15 | 244 |
| Arsenic, poisoning by | 17 | 162 |
| Arsenic, Mr. Rainey on | 17 | 198 |
| Arsenic, Berzelius on the detection of | 19 | 339 |
| Arsenic, poisoning from the fumes of | 19 | 504 |
| Arsenic, effects of a large dose of | 20 | 63 |
| Arterial elasticity and tonicity | 3 | 196 |
| Arterial action not perceptible for 24 hours | 4 | 102 |
| Arterial system, general induration of the | 10 | 174 |
| Arterial system, diseases of the | 14 | 173 |
| Arteries and their branches, relative capacity of | 9 | 101 |
| Arteries, ligature of | 9 | 255 |
| Arteries, Mr. Guthrie on the | 13 | 133 |
| Arteries, wounded, reparative processes in | 13 | 134 |
| Arteries, modes of operating on | 13 | 137 |
| Arteries, wounded, M. Dupuytren on | 13 | 139 |
| Arteries, peculiar affection of the | 13 | 180 |
| Arteries, the deposites in, considered | 13 | 279 |
| Arteries, Mr. Guthrie on diseases of | 13 | 350 |
| Arteries, contractility of | 13 | 351 |
| Arteries, erysipelatous inflammation of | 13 | 352 |
| Arteries, changes of, in age | 13 | 354 |
| Arteries, preternatural, dilatation of | 13 | 356 |
| Arteries, collateral circulation of | 13 | 363 |
| Arteries, torsion of | 15 | 177 |
| Arteries, torsion of, after operations | 17 | 174 |
| Arteries, M. Manec's treatise on | 18 | 67 |
| Arteries, anatomy of | 18 | 68 |
| Arteries, effects of ligatures on | 18 | 70 |
| Arteries, effect of small round ligature | 18 | 72 |
| Arteries, effect of large flat ligature | 18 | 74 |
| Arteries, torsion of | 18 | 75 |
| Arteries, ossified | 18 | 77 |
| Arteries, wounds of | 18 | 77 |
| Arteries, Dr. Smith's anatomy of the, | 18 | 255 |
| Arteries, bruits of the | 19 | 519 |
| Arteriotomy followed by temporal aneurism | 11 | 523 |
| Arteritis, Mr. Howel's case of | 4 | 193 |
| Arteritis | 17 | 146 |
| Arteritis, cases of | 17 | 149 |
| Arteritis and spontaneous gangrene, | 19 | 207 |
| Artery, ligature of, ultrà tumorem | 11 | 469 |
| Artery, subclavian, ligature of | 20 | 206 |
| Artery, gluteal, ligature of the | 20 | 165 |
| Arthritis treated by acupuncture | 15 | 468 |
| Arthrosia atonica, Dr. Wright on | 11 | 173 |
| Articular rheumatism | 7 | 197 |
| Articular inflammation, *Balfourian* practice in | 8 | 156 |
| Articulating cartilages, M. Cruveilhier on | 7 | 165 |
| Articulation, Sir C. Bell on | 18 | 556 |
| Artificial nose, Mr. Snell's | 3 | 305 |
| Artificial aid in parturition | 3 | 408 |
| Artificial anus, Dr. Maitland's case | 4 | 233 |
| Artificial anus successfully treated | 5 | 292 |
| Artificial anus after pregnancy | 5 | 525 |
| Artificial anus, curious case of | 7 | 558 |
| Artificial anus in the vagina | 9 | 184 |
| Artificial anus curious case of | 9 | 276 |
| Artificial anus, new mode of treating, | 9 | 313 |
| Artificial anus, anatomical description of | 9 | 313 |
| Artificial anus, use of the "enterotome" in | 9 | 318 |
| Artificial anus, cases of cure of | 9 | 320 |
| Artificial anus, case of | 9 | 525 |
| Artificial anus after hernia | 10 | 574 |
| Artificial anus opening into the vagina, case of | 12 | 151 |
| Arts, trades, &c. their influence | 14 | 312 |
| Arts, trades, &c. Mr. Thackrah on | 18 | 101 |
| Ascarides, death from | 4 | 287 |
| Ascending paralysis, curious case of | 12 | 161 |
| Ascension, fatal fever at | 2 | 5 |
| Ascherson, M. on congenital fistula of the neck | 20 | 481 |
| Ascites, complicated with pregnancy, | 1 | 492, 500 |
| Ascites, treated by compression | 2 | 530 |
| Ascites and pregnancy combined | 2 | 506 |
| Ascites, its causes and treatment | 4 | 411 |
| Ascites with pregnancy, case of | 6 | 302 |
| Ascites with pregnancy, case of | 7 | 409 |
| Ascites, periodical, a case of | 7 | 227 |
| Ascites cured by wine vapour | 8 | 508 |
| Ascites cured by pressure | 10 | 224 |
| Ascites, apparently cured by perspiration | 10 | 461 |
| Ascites, dissection of a case of | 11 | 266 |
| Ascites, iodine in | 12 | 249 |
| Ascites cured by tapping, case of | 20 | 543 |
| Ascites, curious case of | 20 | 177 |
| Ashburner on sarsaparilla | 19 | 477 |
| Ashwell, Mr. on parturition | 10 | 97 |
| Asphyxia, M. Leroy on | 7 | 234 |
| Asphyxia, Dr. Edwards on | 18 | 17 |
| Asphyxia of new-born children | 19 | 265 |
| Asphyxia, case of, during intoxication | 20 | 196 |
| Associated apothecaries, their annual report | 3 | 309 |
| Associated Apothecaries, their report | 5 | 630 |
| Asthma, fatal, from affection of glottis | 2 | 458 |
| Asthma, a case of | 5 | 217 |
| Asthma, antipathy of the French to the name | 5 | 218 |
| Asthma, M. Broussais on | 7 | 167 |
| Asthma, new remedy for | 8 | 220 |
| Asthma, Mr. Abernethy's pathology of | 8 | 573 |
| Asthma, Laennec on | 9 | 141 |
| Asthma, treatment of | 9 | 143 |
| Asthma cured by lobelia inflata | 9 | 208 |
| Asthma—enormous hypertrophy of the heart | 12 | 463 |
| Asthma, Grinders', Dr. Knight on | 14 | 202 |

| | Vol. | Page |
|---|---|---|
| Asthma & auscultation, Dr. Forbes on | 16 | 475 |
| Astragalus, case of extraction of the | 7 | 457 |
| Astragalus, dislocations of the...... | 20 | 520 |
| Atkinson, Mr. his medical bibliography | 20 | 329 |
| Atlas, disease and dislocation of the, | 10 | 582 |
| Atmosphere, its influence in cholera | 3 | 135 |
| Atmospheric constitution of 1825-6-7 and 8 | 10 | 202 |
| Atmosphere, influence of, on batrachian reptiles | 18 | 2 |
| Atmospheric air, in deafness ...... | 14 | 469 |
| Atmospheric influence on cholera .. | 16 | 202 |
| Atropa belladonna, its properties, &c. | 7 | 98 |
| Atrophy of the spinal marrow...... | 1 | 7 |
| Atrophy from unsuspected causes—singular case of............ | 11 | 241 |
| Atrophy of the fœtal brain ........ | 19 | 510 |
| Audible signs of pregnancy........ | 20 | 100 |
| Aura electrica, remarkable case of.. | 7 | 459 |
| Aural aneurism, successful operation | 15 | 472 |
| Auricle of the heart, ulceration of the | 11 | 446 |
| Auriculo-ventricular contractions .. | 1 | 193 |
| Auscultation, advantages of........ | 1 | 197 |
| Auscultation, Laennec and Forbes on | 2 | 61 |
| Auscultation in pneumonia........ | 3 | 513 |
| Auscultation, Mr. Bennett's Thesis on | 4 | 449 |
| Auscultation, mediate, Dr. Stack's inquiry into ............... | 7 | 297 |
| Auscultation, Laennec's treatise on.. | 8 | 103 |
| Auscultation, debates on .......... | 8 | 470 |
| Auscultation, M. Laennec on ...... | 10 | 420 |
| Auscultation, Dr. Elliotson on .... | 13 | 425 |
| Auscultation, evidence of pregnancy | 14 | 465 |
| Auscultation in pregnancy ........ | 14 | 495 |
| Auscultation applied to the heart .. | 16 | 360 |
| Auscultation, application of, to midwifery.................. | 17 | 163 |
| Auscultation, Dr. Forbes on ...... | 17 | 246 |
| Auscultation of the heart in the fœtus | 17 | 246 |
| Auscultation, as a sign of pregnancy | 17 | 343 |
| Auscultation, obstetric, Dr. Kennedy on | 20 | 92 |
| Auscultation, application of, to still-born children.......... | 20 | 164 |
| Auscultation, mediate, M. Piorry on | 20 | 337 |
| Autenreith, Dr. on softening of the stomach.................... | 9 | 464 |
| Avenbrugger on percussion........ | 2 | 65 |
| Average of forceps cases in midwifery | 3 | 399 |
| Averill, Mr. on loose cartilages . .. | 10 | 181 |
| Axillary aneurism, Mr. Liston's case | 6 | 579 |
| Axillary artery torn, a case of...... | 7 | 452 |
| Axillary nerves torn during the reduction of a luxation ........ | 7 | 454 |
| Axillary aneurism, case of ......... | 8 | 556 |
| Axillary aneurism cured ......... | 15 | 482 |
| Axillary aneurism cured .......... | 15 | 507 |
| Ayre, Dr. on dropsy............... | 4 | 406 |
| Ayre, Dr. on cholera ............. | 19 | 457 |
| **B** | | |
| Babington, Dr. on the black death.. | 19 | 121 |
| Babington, Dr. (Senr.) death of.... | 19 | 161 |
| Bacon, Lord, on medicine ........ | 19 | 439 |
| Bacot, Mr. on syphilis ........... | 12 | 548 |
| Bad health, a cause of distortion.... | 3 | 370 |
| Bahama Islands, climate of the .... | 13 | 78 |
| Baillie, Dr. his works, by Wardrop, | 4 | 115 |
| Baillie, Dr. his opinions as to success in medicine ............ | 4 | 117 |
| Baillie, Dr. his observations on medicine....................... | 4 | 364 |
| Baillie, Dr. anecdote of ......... | 13 | 489 |
| Baird, Dr. unjust treatment of...... | 19 | 446 |
| Baker, Dr. his case of disease of heart | 3 | 529 |
| Balfour, Dr. on hernia ............ | 2 | 212 |
| Balfour, Dr. his striking cures...... | 2 | 234 |
| Ballingall, Dr. on syphilis ........ | 1 | 85 |
| Ballingall, Dr. his case of lithotomy.. | 6 | 83 |
| Ballingall, Dr. his clinical lecture .. | 9 | 186 |
| Ballingall, Dr. his clinical report.... | 9 | 559 |
| Ballingall, Dr. his report .......... | 10 | 175 |
| Ballingall, Dr. on fractures ........ | 11 | 226 |
| Ballingall, Dr. on amputations .... | 11 | 279 |
| Ballingall, Dr. on military surgery .. | 18 | 428 |
| Ballottement, explanation of ...... | 20 | 99 |
| Bally, M. his cases of obliterated arteries ...................... | 7 | 218 |
| Bally, M. on morphine............ | 12 | 545 |
| Bals. copaibæ, extract of, in gonorrhœa.................... | 6 | 499 |
| Bampfield, Mr. obtains the Fothergillian medal ............... | 1 | 251 |
| Bampfield, Mr. on the spine........ | 2 | 112 |
| Bampfield, Mr. his death.......... | 6 | 637 |
| Bandage in large abdominal wounds.. | 3 | 338 |
| Bandagings and plaster in incised wounds ................... | 3 | 546 |
| Bangalore, climate of ............ | 20 | 362 |
| Bann, fever in .................. | 2 | 1 |
| Barbadoes, climate of ............ | 10 | 207 |
| Bardsley, Dr. his hospital facts, &c.. | 12 | 129 |
| Bardsley, Dr. his hospital facts .... | 13 | 333 |
| Baregine, description of .......... | 20 | 232 |
| Bark and arsenic in neuralgia ...... | 10 | 369 |
| Barley water poisoned by arsenic .. | 3 | 290 |
| Barometers; leeches said to be such, | 5 | 59 |
| Baron, Dr. his "Life of Edward Jenner, M.D." . ............ | 7 | 102 |
| Baron, Dr. on iodine ........... | 11 | 451 |
| Baron, Dr. his contributions to pathology .................. | 15 | 246 |
| Barras, Dr. his case of gastralgia.... | 5 | 489 |
| Barras, Dr. on gastralgia & enteralgia | 8 | 45 |
| Barry, Dr. his ideas on the circulation | 3 | 314 |
| Barry, Dr. his views of the circulation, | 4 | 488 |
| Barry, Dr. his treatment of poisoned wounds ............ | 4 | 518 |
| Barry, Dr. on circulation of the blood, | 5 | 313 |
| Barry, Dr. his reclamation ........ | 6 | 293 |
| Barry, Dr. answer to ........... | 6 | 620 |
| Barry, Dr. on the Gibraltar fever.... | 12 | 539 |
| Barry, Dr. on the Gibraltar fever .. | 13 | 337 |
| Barry, Dr. on the Gibraltar epidemic, | 14 | 69 |
| Barry, Dr. his letter to Sir J. M'Grigor, | 14 | 246 |

| | Vol. | Page |
|---|---|---|
| Barry, Sir David, and Dr. Stevens .. | 17 | 422 |
| Barry, Sir David, his visits to Coldbath Prison | 17 | 423 |
| Barry, Sir David, good fortune of .. | 19 | 185 |
| Barry, Sir David, his factory report.. | 20 | 393 |
| Barton, Dr. his case of anchylosis .. | 7 | 272 |
| Basis cranii, imagined fracture of .. | 2 | 466 |
| Bates, Mr. on abdominal inflammation, | 12 | 313 |
| Baths of Liebenzell | 15 | 256 |
| Baths of iodine in scrophula | 16 | 115 |
| Battley, Mr. on the components of opium | 1 | 253, 521 |
| Battley, Mr. his liquor cinchonæ .. | 11 | 484 |
| Baucal, M. his manual of lithotrity, | 12 | 228 |
| Baudelocque's case of the division of the symphysis pubis | 19 | 502 |
| Bayle, M. his views of the causes of madness | 4 | 185 |
| Beale, Mr. on deformities | 13 | 55 |
| Beale, Mr. on distortions of the spine, | 15 | 418 |
| Beatty, Dr. letter from, on premature labour | 1 | 357 |
| Beatty, Dr. on difficult labours | 15 | 91 |
| Beatty, Dr. on uterine hæmorrhage, | 20 | 446 |
| Beaumont, Mr. on fractures | 16 | 247 |
| Beaupré, M. on the effects of cold .. | 5 | 77 |
| Beccaria, Dr. on petechial fever | 19 | 508 |
| Beck, Dr. on infanticide | 1 | 337 |
| Bedford man of war, fever in | 2 | 10 |
| Begbie, Dr. his case of supposed fractured cervix femoris | 4 | 551 |
| Belcher, Dr. William, on enlarged tonsils | 2 | 317 |
| Belcher, Dr. his case of purpura hæmorrhagica | 3 | 284 |
| Belinaye, Mr. on smilax aspera | 19 | 477 |
| Bell, Mr. his lectures against Sir A. Cooper, on the thigh-bone, &c. | 3 | 41 |
| Bell, Dr. his unfortunate case | 4 | 74 |
| Bell's, Mr. G. cases of wounded nerves, | 5 | 245 |
| Bell, Mr. his appendix to his papers on the nerves | 6 | 412 |
| Bell, Mr. on strictures of the rectum, | 7 | 427 |
| Bell, Mr. on secondary hæmorrhage, | 8 | 565 |
| Bell, Mr. on diseases of the bones .. | 10 | 405 |
| Bell, Mr. on diseases of the teeth .. | 11 | 328 |
| Bell, Mr. on diseases of the teeth .. | 11 | 408 |
| Bell, Mr. on cholera | 16 | 186 |
| Bell, Mr. his letter to Sir H. Halford, | 16 | 224 |
| Bell, Sir C. on the human voice | 18 | 552 |
| Bell, Sir Chas. on contracted prepuce | 19 | 282 |
| Bell, Mr. G. H. on diseases of the liver | 19 | 415 |
| Bell, Sir C. doctrine of the nerves confirmed | 20 | 225 |
| Bell, Sir C. letter to the editor | 20 | 228 |
| Belladonna preventive of scarlatina.. | 1 | 490 |
| Belladonna in tic douloureux | 3 | 300 |
| Belladonna, Mr. Chevalier on | 6 | 231 |
| Belladonna, case of poisoning by | 7 | 233 |
| Bellard, M. his cases of paralysis of the portio dura | 3 | 204 |
| Bengal reports on the cholera | 17 | 72 |

| | Vol. | Page |
|---|---|---|
| Bennati, M. on the mechanism of the voice | 18 | 79 |
| Bennett, Mr. his last illness | 15 | 251 |
| Beriberi, Mr. Hamilton on | 5 | 396 |
| Berlin, report of hospital at | 20 | 218 |
| Bermudas, climate of the | 13 | 78 |
| Berzelius, on inorganic bodies | 19 | 337 |
| Beulah Spa, account of | 16 | 554 |
| Bibliographical Record | 3 | 301 |
| Bibliography, medical, Mr. Atkinson's | 20 | 329 |
| Bidois, Dr. his treatment of pericarditis | 3 | 242 |
| Biett, M. on lichen | 11 | 567 |
| Biett, M. on herpes | 11 | 568 |
| Biett, M. on eczema | 11 | 570 |
| Biett, M. on elephantiasis Arabum .. | 11 | 571 |
| Biett, M. his lectures, by Cazenave, | 16 | 382 |
| Biett on diseases of the skin | 17 | 360 |
| Bifid brain, Dr. Duncan's case of | 1 | 297 |
| Bile, Mr. Mayo's experiments on the, | 6 | 247 |
| Bile, alterations in its state | 6 | 515 |
| Bile effused into the substance of the liver | 7 | 274 |
| Bile, alterations of the | 15 | 90 |
| Biliary calculus, Dr. Craigie on | 2 | 159 |
| Biliary ducts, obliteration of the | 3 | 237 |
| Biliary secretion, Dr. Simon's experiments on | 4 | 488 |
| Biliary ducts, diseases of the | 8 | 529 |
| Biliary secretion, Mr. Abernethy on, | 9 | 174 |
| Biliary calculi, recent effects of | 11 | 447 |
| Biliary ducts, diseases of the | 15 | 89 |
| Biliary calculi, deceptive symptoms.. | 15 | 218 |
| Biliary abscess, case of | 19 | 243 |
| Bill for legalising anatomy, Mr. Warburton's | 11 | 218 |
| Bill, factories' regulation, on the | 19 | 181 |
| Billing, Dr. his principles of medicine, | 14 | 592 |
| Biographical sketch of Dr. Armstrong, | 12 | 281 |
| Birmingham Eye Infirmary, report of | 19 | 257 |
| Bische, a malignant dysentery of Trinidad | 6 | 470 |
| Bistoury and knife in extirpation of the tonsils | 3 | 545 |
| Bite from karrait snake | 17 | 525 |
| Bitter almonds, case of poisoning by, | 6 | 293 |
| Black drop, *reminiscences* on the | 5 | 291 |
| Black vomit, its nature considered.. | 6 | 155 |
| Black death, Dr. Babington on | 19 | 121 |
| Blacklock, Mr. on inflation of the bowels | 15 | 267 |
| Blackmore, Dr. on pulmonary consumption | 17 | 400 |
| Bladder, sloughing of, post partum.. | 1 | 235 |
| Bladder opened by Mr. A. C. Hutchison | 2 | 224 |
| Bladder, remarkable disease of | 5 | 287 |
| Bladder, spasm of the | 5 | 502 |
| Bladder, Mr. Thompson's case of distension of the | 5 | 507 |
| Bladder, living worms discharged from, | 5 | 528 |
| Bladder, curious case of perforation of the | 7 | 438 |

| | Vol. | Page |
|---|---|---|
| Bladder, puncture of, Mr. Fereday | 7 | 561 |
| Bladder, remarkable disease of the | 8 | 492 |
| Bladder, paracentesis abdominis thro' | 9 | 224 |
| Bladder mistaken for aneurism of the aorta | 10 | 152 |
| Bladder, case of cancer of the | 15 | 453 |
| Bladder, wound of the | 16 | 59 |
| Bladder, on the form and disposition of the | 16 | 144 |
| Bladder, states of, *quoad* lithotrity | 16 | 152 |
| Bladder, description of | 17 | 27 |
| Bladder, ruptured, case of | 17 | 540 |
| Bladder, Dr. Macfarlane on wounds of the | 18 | 443 |
| Bladder, inversion of the | 18 | 460 |
| Bladder, the removal of clots in the, | 20 | 183 |
| Blake, Dr. on delirium tremens | 13 | 196 |
| Bland, M. on hooping-cough | 15 | 480 |
| Bland, M. his physiology | 15 | 510 |
| Blandin, M. on amputation at the hip-joint | 19 | 195 |
| Blandy, Miss, her trial | 3 | 179 |
| Bleeding in insanity | 3 | 110 |
| Bleeding, *ad deliquium*, in laryngitis | 3 | 207 |
| Bleeding, its effects in a severe case of laryngitis | 3 | 210 |
| Bleeding, in dissection wounds, Mr. Shaw on | 3 | 253 |
| Bleeding, in dissection wounds, Dr. Thomson on | 3 | 256 |
| Bleeding, in difficult parturition from rigidity | 3 | 420 |
| Bleeding successful in a case of irritative fever | 4 | 70 |
| Bleeding in irritative fever, considered | 4 | 77 |
| Bleeding, when indicated | 4 | 111 |
| Bleeding late in fever, its good effects, | 4 | 132 |
| Bleeding in dropsy | 4 | 137 |
| Bleeding in convulsions, Mr. North's sentiments on | 5 | 158 |
| Bleeding, Medicus on | 5 | 232 |
| Bleeding in the cold stage of ague, Dr. Mackintosh on | 7 | 184 |
| Bleeding in erysipelas, successful case | 8 | 475 |
| Bleeding for purpura | 9 | 213 |
| Bleeding, fatal, in puerperal mania | 11 | 364 |
| Bleeding in fever | 12 | 389 |
| Bleeding, diffused aneurism from | 14 | 282 |
| Bleeding, Mr. Wardrop on | 20 | 476 |
| Bleeding, injurious effects of | 20 | 476 |
| Bleedings, immense, in concussion | 13 | 561 |
| Bleedings, small, in hæmoptysis | 15 | 146 |
| Blennorrhagia, acute, M. Ricord on, | 18 | 389 |
| Blennorrhagia, chronic, M. Ricord on | 18 | 390 |
| Blind, eyes for them! | 13 | 481 |
| Blindness, temporary | 1 | 438 |
| Blister, nitrate of silver as a | 10 | 449 |
| Blistered surfaces treated by carded cotton | 12 | 572 |
| Blistering in erysipelas | 9 | 284 |
| Blistering for ulcers | 12 | 474 |
| Blisters, Dr. Kennedy on | 10 | 564 |
| Blood, Dr. Scudamore on | 1 | 137 |
| Blood, its state in epidemic Indian cholera | 4 | 93 |
| Blood, can it be the seat of disease? | 6 | 218 |
| Blood, fluid after death, cases of | 11 | 185 |
| Blood, fœtid fluid injected into the | 11 | 347 |
| Blood of man, peculiar odorous principle in | 11 | 483 |
| Blood, Dr. Hall on the loss of | 13 | 60 |
| Blood, treatment of the loss of | 13 | 67 |
| Blood, Dr. Steevens on the | 13 | 217 |
| Blood, on the particles of the | 13 | 371 |
| Blood, concrete oil in the | 14 | 208 |
| Blood, M. Andral on the | 15 | 337 |
| Blood, forces influencing it | 15 | 337 |
| Blood, alterations in the | 15 | 339 |
| Blood, experiments on the | 15 | 342 |
| Blood, influence of the nerves on the, | 15 | 344 |
| Blood, inflammatory state of the | 15 | 346 |
| Blood, deleterious substances in the | 15 | 346 |
| Blood, state of, in chronic affections, | 15 | 348 |
| Blood, state of, in dropsy, scurvy, &c. | 15 | 350 |
| Blood, M. Piorry on the | 15 | 488 |
| Blood, microscopic researches on the, | 16 | 256 |
| Blood and bloodletting | 17 | 249 |
| Blood, Dr. Stevens on the | 17 | 321 |
| Blood, composition of, in jaundice | 18 | 490 |
| Blood, pathology of, in cholera | 18 | 515 |
| Blood, analysis of the serum | 19 | 502 |
| Blood, animalculæ found in the | 20 | 39 |
| Blood, alterations of, in disease | 20 | 381 |
| Blood, Muller's experiments on the, | 20 | 501 |
| Bloodletting, on the best mode of | 1 | 141 |
| Bloodletting, in cholera morbus | 3 | 138 |
| Bloodletting in tetanus suggested | 3 | 395 |
| Bloodletting, experiments on | 10 | 248 |
| Bloodletting, fatal effects of, in puerperal affections | 11 | 298 |
| Bloodletting, Dr. Marshall Hall on | 12 | 40 |
| Bloodletting in fever | 12 | 399 |
| Bloodletting in inflammation | 13 | 325 |
| Bloodletting in irritation | 13 | 327 |
| Bloodletting in fever | 13 | 412 |
| Bloodvessels, curious distribution of, | 17 | 178 |
| Bloody perspiration | 15 | 496 |
| Bloomfield, Mr. on abscess of tibia | 18 | 36 |
| Bloxam, Mr. case of fracture and dislocation of the femur | 19 | 480 |
| Blow-pipe, use of the | 16 | 381 |
| Blue skin in malformed hearts | 16 | 73 |
| Blue urine | 18 | 541 |
| Blumenbach's physiology, Dr. Elliotson's translation of | 9 | 97 |
| Blundell, Dr. his physiological researches | 2 | 400 |
| Blundell, Dr his extirpation of uterus | 9 | 519 |
| Blundell, Dr. his cases of amputation, | 10 | 215 |
| Blundell, Dr. on animal magnetism | 19 | 184 |
| Board of Health on cholera | 15 | 527 |
| Board of Health, circulars of | 17 | 546, 559 |

| | Vol. | Page |
|---|---|---|
| Boards, medical, on the | 19 | 180 |
| Body, changes of, during existence.. | 12 | 54 |
| Body, discharge of worms from the, | 19 | 168 |
| Boerhaave and Haller, notice of .... | 15 | 410 |
| Boggie, Dr. on hospital gangrene .. | 9 | 323 |
| Boismont, M. on the plica polonica.. | 20 | 215 |
| Boivin, Madame, on abortion ...... | 10 | 257 |
| Boivin, Madame, on polypus uteri and pregnancy | 11 | 571 |
| Boivin, Mad. on supposed rheumatism | 14 | 261 |
| Boivin, Madame, on diseases of the uterus | 18 | 406 |
| Boivin, Madam, on the uterus .... | 19 | 522 |
| Bombay reports on the epidemic cholera | 17 | 57 |
| Bond, Mr. his case of rupture of the perineum | 3 | 288 |
| Bone formed in different organs; case of | 4 | 119 |
| Bone, inflammation of | 10 | 407 |
| Bone, bloody tumor of | 11 | 200 |
| Bones, their thickness—on what dependant? | 4 | 57 |
| Bones of the cranium, disease and removal of the | 11 | 523 |
| Bones, soup prepared from | 17 | 183 |
| Bones, scrofulous disease of the.... | 17 | 439 |
| Bones, mode of cleansing | 20 | 150 |
| Bony union of fractured cervix femoris | 20 | 170 |
| Boott, Dr. his life of Dr. Armstrong, | 18 | 374 |
| Borate of potassa, Dr. Hamilton's catholicon | 2 | 515 |
| Borough-hospital pupils, letter from | 9 | 164 |
| Bostock, Dr. on the mercurial saliva, | 4 | 490 |
| Bostock, Dr. on Thames water...... | 12 | 443 |
| Botany, medical, Mr. Reid's ...... | 17 | 387 |
| Bougie, in stricture of œsophagus .. | 15 | 387 |
| Bougies, use of, in amenorrhœa .... | 20 | 161 |
| Bouillaud, Dr. his cases of angina œdematosa | 3 | 205 |
| Bouillaud and Cruveilhier on the anterior lobes of the brain .... | 7 | 129 |
| Bouillaud, Dr. his case of hypertrophy of the stomach | 7 | 221 |
| Bouillaud, Dr. on pulmonary apoplexy | 7 | 466 |
| Bouillaud, M. on chronic inflammation of the cerebral vessels .. | 10 | 585 |
| Bouillaud, M. on scalds | 19 | 195 |
| Bouillaud on pneumonia | 19 | 225 |
| Bouillaud on the bruits of the heart and arteries | 19 | 519 |
| Bouillaud, M. on typhus fever...... | 20 | 514 |
| Bouillaud, M. on typhus fever...... | 20 | 524 |
| Bourdon, M. on organic disease of stomach | 2 | 174 |
| Bourgeois, M. his case of scirrhus of the stomach | 5 | 206 |
| Bousquet, M. on vaccination ...... | 20 | 417 |
| Bowels, complicated affections of the | 3 | 7 |
| Bowels, extraordinary constipation.. | 12 | 181 |
| Bowels and stomach, morbid anatomy of the | 10 | 118 |
| Bowels, morbid matters in | 13 | 214 |
| Bowels, obscure affection of the .... | 13 | 225 |
| Bowels, fatty discharge from the.... | 20 | 8 |
| Bowen, Dr. on cataract | 1 | 396 |
| Bower, Mr. his clinical report...... | 20 | 561 |
| Boyle, Mr. on the treatment of syphilis | 1 | 172 |
| Boyle, Mr. on moxa | 2 | 377 |
| Boyle, Mr. on the diseases of Africa.. | 16 | 408 |
| Bracco, a magnificent mountain-pass | 14 | 461 |
| Brachet, M. on the ganglionic system, | 14 | 33 |
| Brachial artery, aneurism of the.... | 10 | 540 |
| Brachial artery, aneurism of, from bleeding | 11 | 157 |
| Brain, on the functions of the...... | 1 | 179 |
| Brain, Dr. Lallemand on the pathology of the | 1 | 265 |
| Brain, Dr. Kellie on the pathology of the | 1 | 285 |
| Brain, its consistence and state in inflammation | 4 | 194 |
| Brain, ulcer of, Dr. Scoutteten's case of | 4 | 208 |
| Brain, symptoms of irritability of the, | 4 | 436 |
| Brain, Dr. Gregory's case of hæmatoid tumor of the | 4 | 523 |
| Brain, Dr. Clutterbuck's *primum mobile* in fever | 5 | 7 |
| Brain, its state in convulsions...... | 5 | 153 |
| Brain, Dr. Mill's observations on its diseases | 5 | 350 |
| Brain diseased, without any symptoms, | 6 | 234 |
| Brain, state of, in hooping-cough .. | 6 | 612 |
| Brain, metastasis of rheumatism to.. | 7 | 351 |
| Brain and spinal marrow, diseases of the | 7 | 438 |
| Brain, fatal disease of, mistaken .... | 8 | 454 |
| Brain, Mr. Brodie on injuries of.... | 9 | 400 |
| Brain, hypertrophy of the | 10 | 565 |
| Brain, curious and common condition of its arachnoid membrane, | 11 | 187 |
| Brain, ramollissement of the ...... | 11 | 245 |
| Brain, partial ramollissement of the.. | 12 | 215 |
| Brain and spine, irritation of the.... | 12 | 293 |
| Brain, extensive disease of | 12 | 460 |
| Brain fever, Dr. Burton Pearson on.. | 13 | 198 |
| Brain, opposite causes of disease in.. | 13 | 316 |
| Brain, mollescence of the | 13 | 500 |
| Brain, anomalous symptoms referred to | 13 | 560 |
| Brain, inflamed, from old injury of head | 14 | 231 |
| Brain, abscess of the | 14 | 233 |
| Brain, tumors in | 14 | 545 |
| Brain, sympathetic affections of the, | 15 | 296 |
| Brain, abscess and ulceration of the, | 15 | 317 |
| Brain, softening of the | 15 | 322 |
| Brain, effects of pressure on the .... | 15 | 326 |
| Brain, tumors in the | 16 | 22 |
| Brain, hypertrophy of the | 16 | 26 |

| | Vol. | Page |
|---|---|---|
| Brain, membranous alterations of .. | 16 | 28 |
| Brain, cases of mollescence of...... | 16 | 228 |
| Brain, encysted abscess of ........ | 16 | 255 |
| Brain, on the functions of the...... | 16 | 423 |
| Brain, size of, its influence ........ | 16 | 427 |
| Brain, diseased in insanity ........ | 16 | 427 |
| Brain, inflammation of the ........ | 16 | 598 |
| Brain, ulceration of the .......... | 16 | 601 |
| Brain, Mr. Crampton on inflammation of .................. | 18 | 237 |
| Brain, disturbances of the ........ | 19 | 341 |
| Brain, inflammation of the ........ | 19 | 459 |
| Brain, fœtal, atrophy, of.. ....... | 19 | 510 |
| Brain, asthenic disease of, Dr. Uwins on .................. | 20 | 57 |
| Brande, Mr. his Manual of Pharmacy | 3 | 141 |
| Brandreth's, Dr. case of hydrophobia | 3 | 534 |
| Brandreth's, Dr. case of hydrophobia | 3 | 575 |
| Bransby Cooper's description of a Chinese foot ................ | 12 | 494 |
| Brass-founders, diseases of ........ | 18 | 103 |
| Brazil, medical journal of.......... | 17 | 182 |
| Breast, case of encysted tumor of the, | 7 | 261 |
| Breast, Sir A. Cooper on diseases of the | 10 | 381 |
| Breast, common inflammation of the, | 10 | 384 |
| Breast, chronic abscesses in the .... | 10 | 385 |
| Breast, lacteal swelling of the ...... | 10 | 386 |
| Breast, hydatid disease of the...... | 10 | 386 |
| Breast, chronic tumor of the ...... | 10 | 392 |
| Breast, cartilaginous tumor of the .. | 10 | 394 |
| Breast, adipose tumor of the ...... | 10 | 395 |
| Breast, large and pendulous ...... | 10 | 395 |
| Breast, scrofulous swelling of the .. | 10 | 396 |
| Breast, irritable tumor of the...... | 10 | 397 |
| Breast, ecchymosis of the.......... | 10 | 398 |
| Breast, fungus hæmatodes of the.... | 12 | 238 |
| Breech-presentations, Dr. Ramsbotham on.................... | 17 | 289 |
| Breech-presentations, cases of...... | 17 | 291 |
| Breschet, M. on a new kind of extra-uterine pregnancy ........ | 5 | 518 |
| Breschet, M. on equivocal sanguineous tumors .............. | 7 | 159 |
| Breschet, M. his case of disease of the heart ... .............. | 8 | 151 |
| Bretonneau, Dr. and his theory of fever ...................... | 5 | 199 |
| Bretonneau, Dr. on "diptheritis" .. | 5 | 419 |
| Brewster, Sir David, on ocular illusions.................... | 17 | 467 |
| Brigham, Mr. his case of empyema.. | 18 | 450 |
| Bright, Dr. his reports of medical cases | 8 | 90 |
| Bright, Dr. on dropsical effusion.... | 8 | 313 |
| Bright, Dr. on the intestines in fever, | 10 | 321 |
| Bright's, Dr. report of medical cases, | 12 | 97 |
| Bright, Dr. his medical reports .... | 15 | 289 |
| Bright, Dr. his medical cases ...... | 16 | 1 |
| Bright, Dr. his medical reports .... | 16 | 321 |
| Bright, Dr. on nymphomania ...... | 19 | 468 |
| Bright, Dr. on diseases of the pancreas | 20 | 1 |
| Bright, Dr. on diseases of duodenum, | 20 | 1 |
| Bristol, Medical School of ........ | 20 | 181 |
| Bristol Infirmary, strange exhibition at .................... | 19 | 267 |
| Bristol Medical Conversation Society, | 20 | 188 |
| Britain, progressive changes of mortality in.................... | 11 | 423 |
| British physicians, lives of ........ | 13 | 478 |
| British Madeira.................. | 15 | 495 |
| Brodie, Mr. his theory of cold ...... | 5 | 93 |
| Brodie, Mr. on poisonous matter in offal .................... | 7 | 166 |
| Brodie, Mr. his case of ununited fracture .................... | 7 | 478 |
| Brodie, Mr. his case of diseased breast | 8 | 126 |
| Brodie, Mr. his case of inguinal aneurism ................ | 8 | 547 |
| Brodie, Mr. on injuries of the brain | 9 | 400 |
| Brodie, Mr. on loose cartilages .... | 10 | 525 |
| Brodie, Mr. on aneurism by anastomosis .................. | 11 | 440 |
| Brodie, Mr. on cysts connected with the liver .............. | 11 | 535 |
| Brodie's, Mr. recto-vesical lithotomy, | 14 | 183 |
| Brodie, Mr. on calculous disorders.. | 15 | 129 |
| Brodie, Mr. on abscess of the tibia .. | 18 | 33 |
| Bronchia, on organic changes in the | 1 | 451 |
| Bronchia, cases of dilatation of its tubes .................... | 3 | 501 |
| Bronchia, cherry-stone in the ...... | 6 | 228 |
| Bronchial inflammation and its consequences ................ | 3 | 504 |
| Bronchial disease, new kind of .... | 3 | 526 |
| Bronchitis, account of ............ | 3 | 496 |
| Bronchitis, sputa in .............. | 3 | 506 |
| Bronchitis, Mr. Jowett's case of .... | 5 | 255 |
| Bronchitis, Dr. Williams on ....... | 17 | 250 |
| Bronchocele, iodine in ............ | 2 | 229 |
| Bronchocele, its etiology .......... | 4 | 2 |
| Bronchocele, some fatal cases of .... | 5 | 189 |
| Bronchocele, some cases of, treated with iodine .............. | 5 | 196 |
| Bronchocele, thyroid arteries *tied* for, | 5 | 585 |
| Bronchocele, its etiology .......... | 6 | 243 |
| Bronchocele, successfully treated by seton.................... | 7 | 263 |
| Bronchocele in Lower Canada...... | 13 | 186 |
| Bronchocele, influence of water, as a cause .................... | 13 | 373 |
| Bronchotomy, Mr. Porter on ...... | 6 | 375 |
| Bronchotomy in croup considered .. | 6 | 383 |
| Bronchotomy, for cynanche laryngea, | 7 | 417 |
| Bronchotomy, Dr. Cullen on ...... | 8 | 496 |
| Bronchotomy for laryngitis....... | 10 | 263 |
| Bronchotomy, Mr. Wood on ...... | 18 | 345 |
| Bronchus, tooth lodged in the...... | 20 | 471 |
| Brook, Dr. on liver-cough ........ | 2 | 208 |
| Brookes, miniature bust of ........ | 18 | 581 |
| Brookes, Joshua, portrait of ...... | 20 | 189 |
| Broussais' doctrine, proofs of it .... | 3 | 325 |
| Broussais and Clutterbuck in danger! | 6 | 244 |
| Broussais', M. principles of medicine | 6 | 313 |
| Broussais, his propositions of medicine.................... | 7 | 46 |

| | Vol. | Page |
|---|---|---|
| Broussais, M. & our *leading* Doctors, | 5 | 30 |
| Broussais, M. his hospital report.... | 8 | 142 |
| Broussais, M. Casimir on gymnastics, | 8 | 159 |
| Broussais, M. on English medicine.. | 8 | 205 |
| Broussais, M. on the chronic phlegmasiæ .......... | 17 | 158 |
| Broussais's doctrines, sketch of .... | 18 | 469 |
| Broussaisism and Brunonianism estimated .......... | 3 | 334 |
| Broussaisism *v.* the errors of the schools, a case .......... | 5 | 21 |
| Broussaisism, Dr. Brown on ...... | 10 | 90 |
| Brown's, Mr. case of paracentesis capitis .......... | 1 | 214 |
| Brown, Dr. his treatment of intermittents .......... | 6 | 229 |
| Brown, Dr. on spinal irritation ... | 9 | 219 |
| Brown, Dr. his medical essays...... | 10 | 85 |
| Brown, Dr. on ischuria renalis .... | 10 | 254 |
| Brown, Dr. on the mercurial treatment of fever .......... | 10 | 459 |
| Brown, Dr. on chorea .......... | 11 | 236 |
| Brown, Dr. his letter to Dr. Johnson, | 16 | 301 |
| Brown, Mr. on cholera. .......... | 16 | 584 |
| Brown, M. R. elected a member of the French Institute .......... | 19 | 232 |
| Browne, Sir Thomas, on cremation.. | 13 | 480 |
| Bruit, placentary, Dr. Kennedy on .. | 20 | 101 |
| Bryce, Mr. his new syphonic stomach-pump .......... | 4 | 264 |
| Bryce, Dr. his capital operations.... | 15 | 513 |
| Bryce, Dr. on cholera .......... | 18 | ·195 |
| Bubo treated by iodine.......... | 10 | 131 |
| Buboes, treated by iodine frictions .. | 3 | 277 |
| Buboes, syphilitic and otherwise.... | 3 | 478 |
| Buchanan, Mr. on the ear ........ | 1 | 42 |
| Buchanan, Mr. on acoustics........ | 3 | 111 |
| Buchanan, Mr. on the organ of hearing | 9 | 204 |
| Buchanan, Mr. on diseased joints .. | 10 | 128 |
| Buchanan, Mr. his ligature of the subclavian .......... | 13 | 501 |
| Buchanan, Dr. on lithoplatomy .... | 14 | 265 |
| Buchanan's history of the Glasgow infirmary .......... | 18 | 559 |
| Buchu leaves, in diseases of the bladder | 2 | 485 |
| Buckthorn, is it useful? .......... | 3 | 161 |
| Buffy coat of blood .......... | 1 | 142 |
| Bulimia, extraordinary case of...... | 19 | 206 |
| Bullet, long residence of, in a skull.. | 9 | 212 |
| Bulletins on his late Majesty ...... | 15 | 200 |
| Bunyon, Mr. Lawrence on .. ..... | 13 | 439 |
| Burke, cerebral development of .... | 11 | 193 |
| Burn followed by tetanus ........ | 9 | 553 |
| Burn, cicatrix from a, removal of .. | 20 | 249 |
| Burne, Dr. on typhus fever ........ | 9 | 18 |
| Burne, Dr. review of, on typhus fever | 9 | 121 |
| Burne, Dr. on otorrhœa purulenta.. | 11 | 538 |
| Burne, Dr. on stricture of the colon, | 13 | 228 |
| Burne, Dr. on the pulse .......... | 15 | 474 |
| Burnett, Dr. on fever .......... | 2 | 1 |
| Burnett, Dr. his curious case of epilepsy .......... | 4 | 502 |
| Burnett, Sir W. on a fever at Chatham, | 15 | 561 |
| Burns, Mr. Travers' treatment of .. | 6 | 111 |
| Burns and scalds, discussion on the treatment of .......... | 9 | 156 |
| Burns and scalds .......... | 10 | 237 |
| Burns and scalds .......... | 12 | 199 |
| Burns, carded cotton for.......... | 12 | 570 |
| Burns and scalds, M. Perry on .... | 16 | 257 |
| Burns, cases of.......... | 16 | 259 |
| Burns, our treatment of .......... | 16 | 261 |
| Burns, Mr. Earle on.......... | 17 | 492 |
| Burns, opposite plans of treating.... | 17 | 495 |
| Burns, Mr. Earle's plan .......... | 17 | 496 |
| Burns, effects of stimulants in ..... | 17 | 497 |
| Burns, secondary effects of ........ | 17 | 498 |
| Burns, contractions after.......... | 17 | 499 |
| Burns, M. Dupuytren on.......... | 19 | 72 |
| Burns, Robert, his character ...... | 19 | 322 |
| Burns, and scalds, Dr. Macfarlane on, | 19 | 529 |
| Burnt sponge, objections to its use.. | 4 | 6 |
| Burrows, Dr. his case of catalepsy .. | 9 | 511 |
| Burrows, Dr. on insanity.......... | 10 | 1 |
| Burrows, Dr. and Dr. Gooch ...... | 12 | 278 |
| Burrows, Dr. his letter.......... | 13 | 147 |
| Bursa of the knee-joint, affections of the.......... | 7 | 500 |
| Bury, Mr. his prize hospital report.. | 7 | 249 |
| Bushell, Mr. on sulphate of quinine | 1 | 226 |
| Bushell, Mr. on rupture of the fallopian tube .......... | 1 | 234 |
| Bushnan, Mr. on animalculæ in blood .......... | 20 | 39 |
| Butel, the Chevalier de, on plague .. | 5 | 233 |
| Butter, Dr. on the dock-yard fever.. | 4 | 66 |
| Bye-law of the College of Surgeons.. | 1 | 241 |
| Bye-law of College of Surgeons .... | 5 | 633 |
| Bye-laws of the College of Surgeons, | 15 | 278 |
| Byron, Lord—dissection of ........ | 2 | 164 |
| Byron, Lord, his character ........ | 19 | 327 |

### C

| | Vol. | Page |
|---|---|---|
| Cachexy, purulent, on the ........ | 19 | 213 |
| Cadmium, sulphate, for corneal specks, | 20 | 485 |
| Cæcum, ruptured, Mr. Speer's case of | 7 | 121 |
| Cæcum and colon, displacement of, | 12 | 571 |
| Cæsarian section, self-performed.... | 1 | 236 |
| Cæsarian operation successful...... | 1 | 494 |
| Cæsarian section, substitute for .... | 2 | 522 |
| Cæsarian section, Mr. Ruth's case of, | 3 | 285 |
| Cæsarian section, culpable adoption of, | 3 | 286 |
| Cæsarian section, Drs. Mansfield, Schenk, and Meyer on ........ | 4 | 473 |
| Cæsarian section, *modes* of, reviewed, | 4 | 476 |
| Cæsarian section successful........ | 4 | 578 |
| Cæsarian section, another successful case .......... | 5 | 284 |
| Cæsarian operation, successful case of | 10 | 264 |
| Cæsarian operation on a dead woman | 12 | 521 |
| Cæsarian operation in Germany .... | 19 | 420 |
| Cæsarian operation, case of........ | 20 | 207 |
| Cæsarian operation, case of........ | 20 | 221 |
| Calamine powder, use of, in small-pox, | 20 | 60 |

| | Vol. | Page |
|---|---|---|
| Calcareous affections, statistics of | 20 | 233 |
| Calcareous incrustation on the lens | 20 | 501 |
| Calculi dissolved by galvanism | 1 | 223 |
| Calculi, renal, Mr. Brodie on | 15 | 136 |
| Calculi in the bladder | 15 | 140 |
| Calculi, composition of | 15 | 141 |
| Calculi, encysted | 15 | 142 |
| Calculi, symptoms of | 15 | 143 |
| Calculi, with enlarged prostate | 15 | 143 |
| Calculi in the female bladder | 15 | 257 |
| Calculi, treatment of | 15 | 257 |
| Calculi, dilatation of the urethra for, | 15 | 257 |
| Calculi, solvents and injections for | 15 | 259 |
| Calculi, vesical, forms of | 16 | 146 |
| Calculi of large size, Dr. King on | 17 | 40 |
| Calculi removed by Weiss' dilator | 18 | 224 |
| Calculous disorders, Mr. Brodie on | 15 | 129 |
| Calculus, biliary, a large one passed, | 2 | 159 |
| Calculus in the colon | 3 | 532 |
| Calculus extracted from the female bladder by dilatation; two cases | 5 | 402, 403 |
| Calculus extracted from the urethra, | 15 | 195 |
| Calculus in the bladder, cases of | 18 | 127 |
| Calculus extracted by Weiss' forceps | 18 | 547 |
| Calcutta Medical Society, its Transactions | 5 | 36 |
| Calcutta Society, transactions of the, | 10 | 399 |
| Calcutta, medical society of | 11 | 314 |
| Calcutta society, transactions of | 11 | 371 |
| Calcutta, transactions of the Medical Society of | 20 | 361 |
| Calderini, Dr. his opinion of euphorbia lathyrus | 3 | 273 |
| Calendar, medical; or student's guide | 11 | 70 |
| Calf of leg, malignant tumor in | 18 | 279 |
| Callaway, Mr. case of priapism | 1 | 214 |
| Callous ulcers treated by sheet-lead, | 9 | 451 |
| Callow, Mr. on obstructed colon | 1 | 125 |
| Callow, surgeon, on abscess of the stomach | 2 | 180 |
| Calomel in cholera | 3 | 137 |
| Calomel, scruple doses of, in dysentery | 3 | 271 |
| Calomel, large doses of, in meningitis | 3 | 280 |
| Calomel in Indian diseases | 3 | 576 |
| Calomel, prejudices against | 4 | 253 |
| Calomel—*facts* versus *prejudice* | 4 | 329 |
| Calomel, its action on the secretions, | 4 | 334 |
| Calomel, its inapplicability to the French stomach | 5 | 74 |
| Calomel, its *moderate* doses in the olden time | 5 | 295 |
| Calomel *not* the cause of green stools | 5 | 353 |
| Calomel and opium, effects of in inflammation | 9 | 238 |
| Calomel in cynanche tonsillaris | 13 | 484 |
| Calomel, effects of mixture with bile, &c. | 16 | 611 |
| Calvert, Mr. on hæmorrhoids | 2 | 273 |
| Cambridge school of medicine | 9 | 522 |
| Cameron, Mr. on nitre in scurvy | 12 | 483 |
| Cameron, Mr. his letter on cholera | 16 | 633 |
| Cameron, Mr. on variety in diet | 18 | 94 |

| | Vol. | Page |
|---|---|---|
| Camphor & hyosciamus in gonorrhœa | 4 | 537 |
| Camps and garrisons, diseases of | 18 | 429 |
| Canada, bronchocele in | 13 | 156 |
| Cancer, correction of its fetor | 2 | 249 |
| Cancer, antiphlogistic treatment of | 5 | 286 |
| Cancer of the penis, removal of the part | 6 | 551 |
| Cancer of the penis | 7 | 243 |
| Cancer of the face, M. Lassere on | 9 | 247 |
| Cancer of the cervix uteri | 10 | 159 |
| Cancer of the tongue—arsenic apparently fatal | 10 | 170 |
| Cancer of the rectum, operation for, | 10 | 581 |
| Cancer, origin and nature of | 11 | 394 |
| Cancer of the face | 11 | 395 |
| Cancer, easy fracture of bones in | 11 | 449 |
| Cancer of the kidney, case of | 12 | 81 |
| Cancer treated by compression | 12 | 490 |
| Cancer, mammæ, cases of | 12 | 525 |
| Cancer of the rectum, M. Lisfranc on | 13 | 148 |
| Cancer of the jaw—M. Delpech on | 13 | 546 |
| Cancer, Sir E. Home on | 14 | 44 |
| Cancer, sarsaparilla for | 14 | 48 |
| Cancer of the jaws, Chirurgus on | 14 | 210 |
| Cancer uteri, Dr. Montgomery on | 14 | 266 |
| Cancer of rectum successfully extirpated | 15 | 210 |
| Cancer of the bladder, case of | 15 | 453 |
| Cancer of the stomach mistaken | 16 | 231 |
| Cancer of stomach, without symptoms, | 18 | 467 |
| Cancer of the heart and kidney | 20 | 236 |
| Canceroid ulcers of lip cured, cases, | 10 | 535 |
| Cancerous tumours, cases of | 10 | 226 |
| Cancerous ulcerations of the face | 15 | 196 |
| Cancrum oris infantium, Dr. Coates on | 5 | 586 |
| Candidates for prize-hospital reports, | 6 | 635 |
| Canning, Mr. his speech on contagion | 3 | 294 |
| Cannula for tying the polypus uteri, plan of, &c. | 12 | 328 |
| Cantharides, a case of poisoning by | 4 | 272 |
| Cantharides, poisoning by, a case | 5 | 240 |
| Cantharides, poisoning by | 15 | 156 |
| Capillaries, state of, in inflammation, | 17 | 411 |
| Capitis paracentesis in hydrocephalus | 1 | 214 |
| Captain Moir, trial of | 14 | 150 |
| Carbonate of iron in neuralgia | 1 | 198 |
| Carbonate of iron in neuralgiæ | 6 | 500 |
| Carbonate of iron in neuralgia | 7 | 403 |
| Carbonate of iron, preparation of the, | 13 | 571 |
| Carbonic acid gas in the blood | 1 | 138 |
| Carbuncle, Polish, Dr. Helbich on | 11 | 204 |
| Carbuncle—gangrene of the stomach | 13 | 545 |
| Carbuncle, malignant, in Italy | 19 | 203 |
| Carbuncular inflammation of fingers, | 17 | 418 |
| Carcinoma, Dr. Carswell on | 19 | 186 |
| Carcinoma, varieties of | 20 | 69 |
| Carcinoma, chemical characters of | 20 | 71 |
| Carcinomatous ulceration, cases of, | 15 | 536 |
| Carded cotton for blistered surfaces, | 12 | 572 |
| Cardia, interesting case of cancer of, | 7 | 439 |
| Cardia, stricture of the | 10 | 120 |
| Cardiac disease, M. Breschet on | 1 | 188 |

| | Vol. | Page |
|---|---|---|
| Cardiac veins, great dilatation of the | 7 | 279 |
| Cardiac disease, simulated by helleborus albus | 11 | 196 |
| Carditis, Dr. Gardner's case of | 6 | 79 |
| Carditis, acute idiopathic, description | 11 | 248 |
| Carditis simulating chorea | 11 | 439 |
| Carditis, supposed fatal case of | 12 | 149 |
| Carditis, chronic, case of | 13 | 176 |
| Care-worn countenance | 14 | 449 |
| Caries of the vertebræ, remarkable case of | 4 | 181 |
| Caries of the teeth, Mr. Koecker's opinions on | 6 | 137 |
| Caries, Dr. Nicol on the treatment of | 8 | 441 |
| Carious joints, Dr. Crampton's cases of excision of | 7 | 301 |
| Carlile, Mr. on sounds of the heart | 20 | 134 |
| Carlisle, Sir A. his letter in the Times | 10 | 546 |
| Carlisle, Sir Anthony, eyes for him | 13 | 481 |
| Carlisle, Sir Anthony, on the spleen, | 13 | 486 |
| Carmichael, Mr. on tetanus | 2 | 193 |
| Carmichael, Mr. on the venereal | 3 | 435 |
| Carmichael, Mr. on tracheotomy | 18 | 227 |
| Carmichael, Mr. on phrenitis | 19 | 459 |
| Carmichael, Mr. ligature of the gluteal artery | 20 | 165 |
| Carmichael, Mr. his reclamation | 20 | 285 |
| Carotid artery wounded, curious case | 3 | 244 |
| Carotid, one obliterated, Dr. Baillie's remarks | 4 | 119 |
| Carotid tied *beyond* the aneurism | 4 | 528 |
| Carotid tied by Mr. Travers | 6 | 165 |
| Carotid tied *originally* by Mr. Fleming | 6 | 167 |
| Carotid aneurism, ligature beyond the tumor | 7 | 209 |
| Carotid aneurism, Mr. Lambert's case | 7 | 214 |
| Carotid aneurism (supposed) ligature ultrà tumorem | 12 | 165 |
| "Carotid aneurism" | 12 | 501 |
| Carotid tied for hæmorrhage | 13 | 263 |
| Carotid tied for the same | 13 | 265 |
| Carotid tied for hemiplegia | 15 | 180 |
| Carotid aneurism, operation for | 16 | 520 |
| Carotid, tied for hemiplegia | 18 | 253 |
| Carotid aneurism, Mr. Montgomery's case of | 19 | 448 |
| Carotids tied for aneurism | 13 | 565 |
| Carpo-pedal spasm, case of | 14 | 366 |
| Carriage exercise, M. Coriolis on | 10 | 519 |
| Carswell, Mr. on melanosis | 2 | 154 |
| Carswell, Dr. on morbid anatomy | 18 | 366 |
| Carswell, Dr. on pathological anatomy, | 19 | 137 |
| Carswell, Dr. on carcinoma | 20 | 68 |
| Carswell, Dr. on pathological anatomy, | 20 | 289 |
| Carter, Dr. on occult disease of the chest | 2 | 161 |
| Carter, Dr. his hospital reports | 5 | 195 |
| Cartilaginous degeneration of bone | 10 | 418 |
| Cartwright, Mr. his Essay on syphilis, | 4 | 328 |
| Carus' comparative anatomy | 17 | 501 |
| Casamayor, M. on recto-vaginal fistula | 12 | 151 |
| Case of supposed disease of the heart, | 2 | 461 |
| Case of dropsy, cured by depletion | 3 | 89 |
| Case, specimen of a | 17 | 403 |
| Cases of premature labour cured by mercury | 1 | 357, 358 |
| Cashin, Miss, inquest on | 13 | 572 |
| Castor oil, substitute for | 2 | 249 |
| Castration for diseased testis, case | 13 | 457 |
| Castration—French practice inferior to our's | 13 | 556 |
| Castration, operation of | 14 | 11 |
| Catalepsy, astonishing case of | 4 | 201 |
| Catalepsy, Mr. North's case of | 8 | 217 |
| Catalepsy, curious case of | 9 | 511 |
| Catalepsy, Dr. Duncan's case of | 13 | 250 |
| Catalepsy, case of | 15 | 511 |
| Catalogue, anatomical, of Guy's Hospital | 13 | 367 |
| Cataplasms used in the French Hospitals | 3 | 351 |
| Cataract, early removal of, by absorption | 4 | 234 |
| Cataract, Dr. Gondret's observations, | 5 | 177 |
| Cataract in India, Mr. Richmond on, | 5 | 263 |
| Cataract and glaucoma | 10 | 472 |
| Cataract, fluid, case of | 13 | 238 |
| Cataract, report on | 13 | 239 |
| Cataract, soft, case of | 13 | 239 |
| Cataract, congenital, cases of | 13 | 239 |
| Cataract, anomalous, case of | 13 | 240 |
| Cataract, capsular and hard, cases of, | 13 | 240 |
| Cataract, cases of | 17 | 282 |
| Cataract, M. Dupuytren on | 18 | 295 |
| Cataracts alternating with diabetes | 12 | 176 |
| Catarrh, suffocative, on | 19 | 441 |
| Catarrhal ophthalmia | 14 | 401 |
| Catarrho-rheumatic ophthalmia, Mr. Mackenzie on | 7 | 136 |
| Catarrhus æstivus, Dr. Bostock on the | 9 | 462 |
| Catarrhus vesicæ of old people | 11 | 210 |
| Cath. de Medicis, sterile for 10 years, | 20 | 159 |
| Cathartine in senna leaves | 3 | 158 |
| Catheter slipped into bladder; lithotomy | 17 | 528 |
| Catheter, death from introduction of | 18 | 249 |
| Catheterism, Mr. Phillips on | 19 | 112 |
| Cato's mode of reducing dislocations, | 15 | 399 |
| Causes of artificial abortion | 1 | 341 |
| Caustic in inflamed absorbents | 6 | 246 |
| Caustic for hydrophobia | 13 | 436 |
| Cautery, actual, its virtues | 4 | 219 |
| Caution necessary in using the forceps | 3 | 432 |
| Cava inferior, obliteration of the | 13 | 550 |
| Cava, inferior, obliteration of | 15 | 569 |
| Cave, vapour, at Pyrmont | 20 | 201 |
| Cayol, M. his views of fever | 2 | 463 |
| Cazenave, M. on elephantiasis | 11 | 492 |
| Cazenave on diseases of the skin | 16 | 382 |
| Cazenave on diseases of skin | 17 | 360 |
| Celibacy in those disposed to insanity | 13 | 90 |
| Cellular texture, inflammation of | 2 | 335 |
| Cellular texture, diffuse inflammation of | 4 | 255 |

| | Vol. | Page |
|---|---|---|
| Cellular inflammation, Dr. Duncan on | 3 | 212 |
| Cellular inflammation from poisoned wound | 5 | 366 |
| Cellular inflammation, Mr. Earle on, | 6 | 535 |
| Cellular inflammation, fatal case of.. | 9 | 450 |
| Celsus, Dr. Collier's | 16 | 156 |
| Celsus, Mr. Lee's | 16 | 157 |
| Celsus, was he a practitioner? | 16 | 159 |
| Cephalæmatomia, description of.... | 20 | 210 |
| Cephalalgia, severe case of, cured by quinine | 1 | 226 |
| Cephalitis simulating hepatitis .... | 3 | 216 |
| Cephaloma, Dr. Carswell on | 19 | 187 |
| Cephalo-spinal fluid | 7 | 228 |
| Cerebellum, case of hydatid of | 4 | 525 |
| Cerebellum, organic disease of the .. | 9 | 542 |
| Cerebellum, abscess of | 14 | 547 |
| Cerebellum, injuries and diseases of, | 15 | 15 |
| Cerebellum, deficiency of | 17 | 177 |
| Cerebellum, abscess of | 19 | 509 |
| Cerebral affection | 2 | 192 |
| Cerebral and worm fever, parallel between | 5 | 219 |
| Cerebral affection, Dr. Latham's case | 5 | 577 |
| Cerebral agenesis, memoir on | 7 | 420 |
| Cerebral hæmorrhage, curious cases of | 8 | 148 |
| Cerebral injury, curious case of.... | 9 | 448 |
| Cerebral affection, periodical | 10 | 195 |
| Cerebral and spinal irritation, Dr. Darwall on | 11 | 433 |
| Cerebral complication in fever | 12 | 349 |
| Cerebral disease, rapidly fatal case of, | 12 | 458 |
| Cerebral artery, middle, ruptured .. | 14 | 234 |
| Cerebral inflammation, Dr. Bright on, | 15 | 291 |
| Cerebral pressure, Dr. Bright on.... | 16 | 2 |
| Cerebral effusion and diseased kidney | 16 | 5 |
| Cerebral pressure from injury | 16 | 33 |
| Cerebral obstruction, Mr. Howship on | 18 | 44 |
| Cerebral diseases, Dr. Paine on .... | 20 | 433 |
| Cerebral affection, complicated with jaundice | 20 | 479 |
| Cerebritis, Dr. Crawford on | 16 | 599 |
| Cerebro-spinal irritation, dangers of the doctrine | 12 | 297 |
| Cerumen, redundancy or deficiency of | 10 | 47 |
| Cerumenous glands, defective action, | 3 | 114 |
| Cervical vertebra fractured | 7 | 240 |
| Cervical vertebra dislocated, without fracture | 7 | 243 |
| Cervical vertebræ, luxation of the . | 8 | 222 |
| Cervical vertebræ, fracture of | 8 | 452 |
| Cervical vertebræ, spontaneous luxation of | 10 | 455 |
| Cervical vertebræ, supposed caries of | 10 | 456 |
| Cervical vertebræ, first and second caries of the | 10 | 582 |
| Cervix femoris; supposed case of fracture | 4 | 551 |
| Cervix femoris, fracture of the ..... | 6 | 272 |
| Cervix femoris, fracture of the . . .. | 6 | 299 |
| Cervix scapulæ, case of fracture of the | 7 | 267 |
| Cervix femoris, fracture of the .... | 8 | 437 |

| | Vol. | Page |
|---|---|---|
| Cervix humeri, fracture of the | 9 | 235 |
| Cervix uteri, state of, in pregnancy.. | 12 | 44 |
| Cervix uteri, inflamed, symptoms of, | 13 | 420 |
| Cervix femoris, Mr. Green on fracture of the | 17 | 150 |
| Cervix femoris fractured, bony union, | 20 | 170 |
| Chalmeil, Dr. on the paralysis attendant on insanity | 6 | 362 |
| Chalybeate springs of Harrogate.... | 15 | 356 |
| Chambers, Dr. his cases of fever.... | 6 | 161 |
| Chambers, Dr. on pulmonary abscess | 8 | 138 |
| Chancre and gonorrhœa considered, | 3 | 448 |
| Chancre, definition of | 3 | 477 |
| Chancre, cases of | 15 | 225 |
| Change of air, its beneficial effect .. | 3 | 30 |
| Change of air, Dr. Johnson on | 14 | 448 |
| Change of air, Dr. Ward on | 15 | 189 |
| Changes of structure in man, &c. Dr. Baron on | 9 | 35 |
| Chaplains and veterinary surgeons .. | 13 | 113 |
| Charcoal, suffocation from the fumes, | 16 | 4 |
| Charité, reports from that hospital, | 4 | 196 |
| Charles, King, his letter to the College of Physicians | 7 | 515 |
| Charter of the College of Physicians, | 7 | 509 |
| Cheap anatomy and its consequences, | 4 | 550 |
| Cheek, remarkable enlargement of.. | 10 | 172 |
| Chemical analysis in cases of poisoning | 12 | 371 |
| Chemistry, Dr. Reid's elements of.. | 16 | 374 |
| Cherry-laurel water for neuralgia . . | 20 | 480 |
| Cheselden's mode of lithotomy .... | 1 | 207 |
| Chest, cases of penetrating wounds of | 3 | 314 |
| Chest, report on the diseases of the.. | 13 | 446 |
| Chest, rheumatic affection of the .. | 14 | 489 |
| Chevalier, Mr. on effusion in the spinal canal | 1 | 23 |
| Chevenix, Mr. on animal magnetism, | 10 | 560 |
| Cheyne, Dr. on a fatal erethism of the stomach | 7 | 124 |
| Cheyne, Dr. on hæmoptysis | 15 | 146 |
| Chiappa, Dr. on pellagra | 19 | 507 |
| Chick, on the evolution of the | 18 | 173 |
| Chilblains, a "morceau" for | 3 | 353 |
| Children, suffocation of | 1 | 351 |
| Children, drowning, strangulation, hanging of | 1 | 351 |
| Children, Dr. Kennedy on the management of | 3 | 485 |
| Children, food and education of .... | 9 | 146 |
| Children, Mr. Marley on diseases of, | 12 | 553 |
| Children, M. Guersent on diseases of, | 13 | 552 |
| Children, partial fractures in | 16 | 588 |
| Chimney-sweeper's cancer, Sir A. Cooper on | 14 | 29 |
| Chimney-sweeper's cancer, Mr. Earle, | 17 | 153 |
| Chinese foot, anatomical description, | 12 | 494 |
| Chinnock, Mr. his case of phlebitis.. | 11 | 155 |
| Chirurgical knowledge | 6 | 638 |
| Chisholm, Dr. on melena | 1 | 231 |
| Chisholm, Dr. on tape-worm | 1 | 232 |
| Chlorate of soda, Dr. Darling on.... | 4 | 574 |
| Chlorate of lime, effects of | 10 | 222 |

| Entry | Vol. | Page |
|---|---|---|
| Chlorates of soda and lime, Mr. Scott on the | 6 | 349 |
| Chloride of lime, Mr. Davidson on | 8 | 566 |
| Chloride of lime between the decks of ships | 9 | 458 |
| Chlorine, as a preventive of hydrophobia | 13 | 433 |
| Chlorine in consumption | 14 | 103 |
| Chlorine, inhalation of, in phthisis | 15 | 208 |
| Chlorosis, amenorrhœa, &c. cases of, | 12 | 186 |
| Chlorosis, diagnosis of | 19 | 196 |
| Chlorotic diseases, memoir on | 17 | 179 |
| Chlorotic diseases, cases of | 17 | 180 |
| Chloruret of lime, its antiseptic powers | 5 | 298 |
| Chloruret of soda, formula for its preparation | 6 | 350 |
| Chloruret of lime, Dr. Reid on | 8 | 38 |
| Chloruret of lime, uncertain composition of | 8 | 43 |
| Chlorurets of soda and lime, Mr. Alcock on the | 6 | 349 |
| Cholera morbus of India, Mr. Cormick on | 1 | 481 |
| Cholera, Mr. Scot's report of | 3 | 121 |
| Cholera morbus, Dr. Ainslie on | 4 | 258 |
| Cholera morbus, Mr. Montgomery's cases of | 5 | 259 |
| Cholera, Dr. Christie on | 10 | 123 |
| Cholera, treatment of | 10 | 127 |
| Cholera, Mr. Searle on | 13 | 375 |
| Cholera morbus in Russia | 14 | 191 |
| Cholera of India, Dr. Smith on | 14 | 347 |
| Cholera, nature of | 14 | 348 |
| Cholera, symptoms of | 14 | 350 |
| Cholera, treatment of | 14 | 350 |
| Cholera, M. Andral on | 15 | 208 |
| Cholera morbus, remarks on the | 15 | 279 |
| Cholera morbus, Dr. Drysen's report, | 15 | 280 |
| Cholera morbus, Dr. Todd on | 15 | 285 |
| Cholera morbus, Mr. Herrmann | 15 | 285 |
| Cholera of India and Russia | 15 | 447 |
| Cholera, our treatment of | 15 | 448 |
| Cholera, mode of preventing | 15 | 463 |
| Cholera, papers on | 15 | 527 |
| Cholera, post-mortem appearances in | 15 | 528 |
| Cholera, Drs. Russell and Barry on | 15 | 529 |
| Cholera, symptoms of | 15 | 529 |
| Cholera at Port Glasgow | 16 | 130 |
| Cholera from fruit | 16 | 133 |
| Cholera, the epidemic | 16 | 163 |
| Cholera, Prof. Lichtenstadt on | 16 | 164 |
| Cholera at Orenburg in Aug. 1829 | 16 | 164 |
| Cholera, convulsions after death in, | 16 | 165 |
| Cholera, not *imported* into Orenburg, | 16 | 165 |
| Cholera, Sir William Crichton on | 16 | 168 |
| Cholera, its introduction into Russia, | 16 | 168 |
| Cholera, causes and prevention of | 16 | 169 |
| Cholera in Moscow | 16 | 170 |
| Cholera, Moscow report on the | 16 | 172 |
| Cholera, Dr. Walker's letter on | 16 | 175 |
| Cholera, Sir W. Pym's letter on | 16 | 177 |
| Cholera, Sir H. Halford's first letter, | 16 | 178 |
| Cholera, Sir Henry's second letter on | 16 | 179 |
| Cholera, Mr. Kennedy on | 16 | 180 |
| Cholera, Mr. Neale on | 16 | 181 |
| Cholera, Mr. Bell on | 16 | 186 |
| Cholera, bleeding in | 16 | 187 |
| Cholera, affection of the ganglionic system in | 16 | 188 |
| Cholera, mode of propagation of | 16 | 189 |
| Cholera, singularities of | 16 | 190 |
| Cholera, stated to accompany troops, | 16 | 191 |
| Cholera, mischiefs of restrictive cordons in | 16 | 192 |
| Cholera, venæsection in | 16 | 192 |
| Cholera, Mr. Orton on | 16 | 193 |
| Cholera, symptoms of | 16 | 194 |
| Cholera, post-mortem appearances | 16 | 196 |
| Cholera, proximate cause of | 16 | 197 |
| Cholera, compared with other epidemics | 16 | 198 |
| Cholera, contagiousness of | 16 | 199 |
| Cholera, atmospheric influence in | 16 | 202 |
| Cholera, influence of locality in | 16 | 205 |
| Cholera along rivers, considered | 16 | 206 |
| Cholera, susceptibility of mankind to, | 16 | 208 |
| Cholera, treatment of | 16 | 209 |
| Cholera, Mr. Searle on | 16 | 211 |
| Cholera, cause and treatment of | 16 | 211 |
| Cholera, Dr. Lefevre on | 16 | 213 |
| Cholera at St. Petersburgh | 16 | 214 |
| Cholera, symptoms of | 16 | 215 |
| Cholera, primary fatality of | 16 | 216 |
| Cholera, treatment of | 16 | 216 |
| Cholera, Dr. Young on | 16 | 217 |
| Cholera, its progress | 16 | 218 |
| Cholera, origin of, at many places | 16 | 219 |
| Cholera, non-contagiousness of | 16 | 220 |
| Cholera, miscellaneous works on | 16 | 221 |
| Cholera in England | 16 | 266 |
| Cholera, ultra and moderate Boards, | 16 | 267 |
| Cholera, Dr. Johnson's opinions on, | 16 | 268 |
| Cholera as contagious as typhus | 16 | 268 |
| Cholera in Newcastle and Sunderland, | 16 | 269 |
| Cholera constitution of 1831 | 16 | 270 |
| Cholera in St. James's workhouse | 16 | 271 |
| Cholera in Piccadilly | 16 | 272 |
| Cholera, is it a new disease? | 16 | 273 |
| Cholera, reputed peculiarities of | 16 | 273 |
| Cholera, Dr. Johnson's propositions, | 16 | 27[illegible] |
| Cholera, Drs. Russell and Barry on | 16 | 277 |
| Cholera in Sunderland, Dr. Tinn on, | 16 | 280 |
| Cholera, how propagated | 16 | 281 |
| Cholera, a *Chinese* blessing | 16 | 283 |
| Cholera, Dr. Sanders' letter on | 16 | 297 |
| Cholera, Dr. Brown's letter on | 16 | 301 |
| Cholera, Dr. Negri on | 16 | 303 |
| Cholera as it is, our views of | 16 | 304 |
| Cholera at Danzig, Dr. Hamett on | 16 | 305 |
| Cholera, its origin in that city | 16 | 305 |
| Cholera, its prevalence there | 16 | 307 |
| Cholera, its symptoms there | 16 | 308 |
| Cholera, its pathologic characters there | 16 | 310 |
| Cholera, treatment of, there | 16 | 313 |

| | Vol. | Page |
|---|---|---|
| Cholera, general treatment of...... | 16 | 315 |
| Cholera, contagion of, considered by facts.................... | 16 | 318 |
| Cholera, Mr. Bell on, 2d edition.... | 16 | 434 |
| Cholera at Haddington............ | 16 | 435 |
| Cholera at Newton .............. | 16 | 437 |
| Cholera, Dr. Ainslie on .......... | 16 | 439 |
| Cholera, Mr. Thackrah on ........ | 16 | 441 |
| Cholera at Leeds; remarkable...... | 16 | 442 |
| Cholera, on the etiology of the .... | 16 | 484 |
| Cholera, Orton's theory of ........ | 16 | 485 |
| Cholera, Russian embassy on...... | 16 | 489 |
| Cholera, O'Shaughnessy & Clanny on ...................... | 16 | 496 |
| Cholera, report of Madras Board on, | 16 | 497 |
| Cholera at Arcot, in 1787 .......... | 16 | 499 |
| Cholera described by Bontius...... | 16 | 499 |
| Cholera described by Dr. Paisley, in 1774.................... | 16 | 501 |
| Cholera described by Sonnerat in 1774 | 16 | 501 |
| Cholera at the Mauritius.......... | 16 | 502 |
| Cholera at Ganjam in 1781........ | 16 | 503 |
| Cholera described by Curtis in 1782, | 16 | 503 |
| Cholera described by Girdlestone ... | 16 | 504 |
| Cholera described by Dr. Duffin in 1787 .................. | 16 | 505 |
| Cholera described by Dr. Davis in 1787 | 16 | 506 |
| Cholera described by Mr. Thompson, | 16 | 507 |
| Cholera in the Northern Circars, in 1790 ...................... | 16 | 507 |
| Cholera since 1787 .............. | 16 | 508 |
| Cholera in 1814.................. | 16 | 509 |
| Cholera in Travancore............ | 16 | 510 |
| Cholera, names of .............. | 16 | 512 |
| Cholera at Sunderland, Dr. Ogden on | 16 | 533 |
| Cholera at Sunderland, &c. Dr. Lawrie on ................ | 16 | 541 |
| Cholera at Gateshead, Dr. White on, | 16 | 541 |
| Cholera at Kirkintilloch ......... | 16 | 542 |
| Cholera hospitals, Dr. Lawrie on.... | 16 | 544 |
| Cholera, endemic character of...... | 16 | 546 |
| Cholera, predisposition to ........ | 16 | 546 |
| Cholera, symptoms of............ | 16 | 548 |
| Cholera, treatment of ............ | 16 | 550 |
| Cholera at Sunderland, Mr. Greenhow on .................. | 16 | 555 |
| Cholera; the ultra-contagionists ... | 16 | 556 |
| Cholera; analysis of its symptoms.. | 16 | 557 |
| Cholera, treatment of............ | 16 | 558 |
| Cholera, causes of.............. | 16 | 561 |
| Cholera, influence of fear in causing, | 16 | 562 |
| Cholera, inefficacy of quarantine against .................... | 16 | 564 |
| Cholera, arguments for contagion of, | 16 | 566 |
| Cholera hospitals, mortality of .... | 16 | 570 |
| Cholera at Musselburgh, Mr. Moir on | 16 | 572 |
| Cholera; Edinburgh contagionists.. | 16 | 572 |
| Cholera, erratic nature of......... | 16 | 575 |
| Cholera, Mr. Dodd on ............ | 16 | 576 |
| Cholera, Dr. Fergusson on ........ | 16 | 580 |
| Cholera; effects of fear .......... | 16 | 583 |
| Cholera, Mr. Brown on .......... | 16 | 584 |
| Cholera, fumigation in............ | 16 | 586 |
| Cholera and cheap whiskey........ | 16 | 587 |
| Cholera, Dr. Clarke's case of ..... | 16 | 605 |
| Cholera, Dr. O'Halloran on........ | 16 | 608 |
| Cholera, description of, by the Madras Board .................. | 16 | 609 |
| Cholera, varieties in.............. | 16 | 610 |
| Cholera, particular symptoms considered..................... | 16 | 613 |
| Cholera, rapid recovery from, in Madras .................... | 16 | 616 |
| Cholera, state of blood in......... | 16 | 617 |
| Cholera, terminations of, in Madras, | 16 | 618 |
| Cholera, morbid appearances of .... | 16 | 620 |
| Cholera, tabular analysis of the individual Madras reports on .... | 16 | 622 |
| Cholera, Editor's remarks on ...... | 16 | 627 |
| Cholera, consecutive fever in Asia and Europe .................. | 16 | 629 |
| Cholera described by former authors, | 16 | 631 |
| Cholera at Haddington, Drs. Lorimer and Burton on .......... | 16 | 640 |
| Cholera, sporadic, in many villages.. | 16 | 643 |
| Cholera, treatment of, at Haddington, | 16 | 644 |
| Cholera, progress of, in England.... | 16 | 645 |
| Cholera at Sunderland............ | 16 | 645 |
| Cholera at Newcastle ............ | 16 | 647 |
| Cholera, North and South Shields,.. | 16 | 648 |
| Cholera, Gateshead .............. | 16 | 649 |
| Cholera, Haddington ............ | 16 | 650 |
| Cholera, Tranent ................ | 16 | 651 |
| Cholera, Preston Pans. .......... | 16 | 652 |
| Cholera, Edinburgh .............. | 16 | 652 |
| Cholera, propagation by contagion.. | 16 | 653 |
| Cholera, symptoms of ............ | 16 | 655 |
| Cholera; premonitory diarrhœa.... | 16 | 656 |
| Cholera, cases of ................ | 16 | 657 |
| Cholera, cadaveric appearances in .. | 16 | 660 |
| Cholera; ratio signorum.......... | 16 | 665 |
| Cholera, treatment of ............ | 16 | 669 |
| Cholera, causes of .............. | 16 | 672 |
| Cholera, Dr. Yates' letter on ...... | 16 | 674 |
| Cholera, concluding remarks on.... | 16 | 675 |
| Cholera, address to the profession, on a cholera association ...... | 16 | 676 |
| Cholera, Bombay reports on the.... | 17 | 57 |
| Cholera, commencement of, in Bombay .......................... | 17 | 59 |
| Cholera, predisposing causes of .... | 17 | 62 |
| Cholera, treatment of ............ | 17 | 63 |
| Cholera, analysis of cases of ...... | 17 | 65 |
| Cholera, abstract of cases.......... | 17 | 69 |
| Cholera, Bengal report on the...... | 17 | 72 |
| Cholera, atmospheric changes previous to .................... | 17 | 74 |
| Cholera at Jessore .............. | 17 | 76 |
| Cholera, progress of.............. | 17 | 76 |
| Cholera, ravages of, in the grand army, | 17 | 79 |
| Cholera, eccentricity of, in Bengal .. | 17 | 85 |
| Cholera, symptoms of ............ | 17 | 88 |
| Cholera, consecutive fever ........ | 17 | 90 |
| Cholera, remote causes of the.... . | 17 | 93 |

| | Vol. | Page |
|---|---|---|
| Cholera, influence of locality on .... | 17 | 96 |
| Cholera, contagious nature of the .. | 17 | 98 |
| Cholera, instances of immunity from, | 17 | 103 |
| Cholera, mortality of, in the army .. | 17 | 109 |
| Cholera of Newburn, Dr. Craigie on, | 17 | 121 |
| Cholera, propagation of .......... | 17 | 128 |
| Cholera, Dr. Kirk on the. ........ | 17 | 159 |
| Cholera, Baron Dupuytren's letter on, | 17 | 166 |
| Cholera, Dr. Ochel's letter on...... | 17 | 171 |
| Cholera, Professor Delpech's letter on, | 17 | 174 |
| Cholera, account of, in Paris ....... | 17 | 189 |
| Cholera in Vienna, account of...... | 17 | 199 |
| Cholera, Broussais' lectures on .... | 17 | 199 |
| Cholera, Mr. Lizars' letter on... .. | 17 | 229 |
| Cholera, importation of .......... | 17 | 234 |
| Cholera at Paris ... ...... ..... | 17 | 234 |
| Cholera at Ely .. ............... | 17 | 235 |
| Cholera, Baron Heurteloup on .... | 17 | 236 |
| Cholera, modification of opinion on.. | 17 | 237 |
| Cholera in Glasgow .............. | 17 | 238 |
| Cholera in Cork ................ | 17 | 239 |
| Cholera, saline injections in........ | 17 | 240 |
| Cholera in the ship Brutus ........ | 17 | 285 |
| Cholera in Cold-bath Fields ...... | 17 | 330 |
| Cholera, Dr. Stevens' treatment of.. | 17 | 331 |
| Cholera again in Cold-bath Fields .. | 17 | 335 |
| Cholera, Dr. Webster on.......... | 17 | 383 |
| Cholera, French mandate on ...... | 17 | 445 |
| Cholera, French official report on .. | 17 | 448 |
| Cholera, remarks on.............. | 17 | 544 |
| Cholera, contagiousness of ........ | 17 | 545 |
| Cholera, circular of Board of Health, | 17 | 546 |
| Cholera, moveable commission for .. | 17 | 547 |
| Cholera—Manchester riot ........ | 17 | 548 |
| Cholera Excerpta ................ | 17 | 550 |
| Cholera, saline injections into the veins, | 17 | 550 |
| Cholera, iodine for .............. | 17 | 550 |
| Cholera, bandaging the abdomen for, | 17 | 551 |
| Cholera, anodyne paste for ........ | 17 | 551 |
| Cholera, cold water for .......... | 17 | 551 |
| Cholera, narcotic glysters for ...... | 17 | 552 |
| Cholera, prohibition of drink for.... | 17 | 553 |
| Cholera, mortality from, in London. | 17 | 553 |
| Cholera, Dr. Gregory on .......... | 17 | 554 |
| Cholera, salines unsuccessful in .... | 17 | 555 |
| Cholera, Dr. Lindsey's treatment of, | 17 | 555 |
| Cholera, treatment of, at Leeds .... | 17 | 556 |
| Cholera, cured by calomel and opium, | 17 | 556 |
| Cholera, a logical deduction........ | 17 | 556 |
| Cholera, cold water in............ | 17 | 556 |
| Cholera, MM. Foville & Parchappe on, | 17 | 558 |
| Cholera, last circular on .......... | 17 | 559 |
| Cholera, editor's remarks on ...... | 17 | 562 |
| Cholera, epidemic, Mr. Corbyn on .. | 18 | 63 |
| Cholera, Mr. Corbyn's treatment of.. | 18 | 64 |
| Cholera, previous to 1816 ........ | 18 | 66 |
| Cholera at Dantzick, Dr. Hamett on | 18 | 136 |
| Cholera, Dr. M'Cormack on .. ... | 18 | 152 |
| Cholera simulated by gastro-enterite, | 18 | 173 |
| Cholera, different modes of treatment of, in Paris ............ | 18 | 185 |
| Cholera anecdotes................ | 18 | 186 |
| Cholera, occurrences antecedent to its outbreak ................ | 18 | 186 |
| Cholera, etiology of, in Glasgow.... | 18 | 195 |
| Cholera, Mr. Watt on ........... | 18 | 198 |
| Cholera, proximate cause of ...... | 18 | 199 |
| Cholera in Scotland .............. | 18 | 216 |
| Cholera, what is its contagion ? .... | 18 | 245 |
| Cholera, Dr. Haslewood and Mr. Mordey on................... | 18 | 261 |
| Cholera, Dr. Keir on ............ | 18 | 262 |
| Cholera, Mr. Morrah on cupri sulphas in ................ .. | 18 | 263 |
| Cholera, Dr. Tweedie on .......... | 18 | 263 |
| Cholera, Mr. Steward on .......... | 18 | 264 |
| Cholera, Dr. Ayre on ............ | 18 | 264 |
| Cholera, Mr. Hickman on ........ | 18 | 264 |
| Cholera, Messrs. Tweedie & Gazelee on, | 18 | 265 |
| Cholera, letter on .... ........... | 18 | 268 |
| Cholera, Dr. Thomson on ........ | 18 | 269 |
| Cholera, charcoal in............. | 18 | 272 |
| Cholera, Dr. Williams on.......... | 18 | 272 |
| Cholera, cutaneous eruption in .... | 18 | 466 |
| Cholera and sweating sickness at Pontoise.................... | 18 | 514 |
| Cholera, pathology of the blood in.. | 18 | 515 |
| Cholera in France, mortality from .. | 19 | 212 |
| Cholera, lectures on, by Majendie .. | 19 | 244 |
| Cholera, effects of, upon the eye.... | 19 | 447 |
| Cholera, Dr. Ayre on ............ | 19 | 457 |
| Cholera, state regulations in Saxe-Weimar against the .......... | 19 | 513 |
| Cholera, Dr. Froriep on .......... | 19 | 514 |
| Cholera on board the Melpomene frigate ...................... | 19 | 528 |
| Cholera, state of intestinal glands in, | 20 | 67 |
| Cholera, Cruveilhier on the........ | 20 | 74 |
| Cholera, state of alimentary tube in.. | 20 | 76 |
| Cholera, Asiatic, Mr. Langford on .. | 20 | 145 |
| Cholera in Philadelphia .......... | 20 | 178 |
| Cholera, Dr. Tytler's doctrine of.. .. | 20 | 180 |
| Cholera at New Orleans .......... | 20 | 239 |
| Cholera, treatment of ............ | 20 | 505 |
| Choleraphobia in Ireland.......... | 18 | 268 |
| Choleraphobia in Scotland ........ | 20 | 456 |
| Chomel, M. his report from La Charité | 9 | 250 |
| Chorea, various authors on........ | 3 | 563 |
| Chorea, iodine in ................ | 4 | 9 |
| Chorea, Dr. Venables on .......... | 4 | 260 |
| Chorea, Dr. M'Andrew on ........ | 6 | 189 |
| Chorea, Dr. Hawkins' fatal case of.. | 7 | 146 |
| Chorea, large doses of arsenic in.... | 7 | 164 |
| Chorea, Mr. Turner on............ | 7 | 569 |
| Chorea, fatal case of.......... .... | 11 | 236 |
| Chorea, some observations on...... | 11 | 481 |
| Chorea, Dr. Bright on ............ | 16 | 328 |
| Choroid coat, Mr. Mackenzie on.... | 13 | 253 |
| Choroiditis, symptoms of.......... | 13 | 253 |
| Choroiditis, constitutional symptoms | 13 | 255 |
| Choroiditis, causes of ............ | 13 | 256 |
| Choroiditis, Mr. Mackenzie on...... | 15 | 63 |
| Choroiditis, treatment of.......... | 15 | 66 |
| Christison, Dr. on arsenic ........ | 7 | 379 |

| | Vol. | Page |
|---|---|---|
| Christison, Dr. his treatise on poisons | 12 | 362 |
| Christison, Dr. on lead | 12 | 491 |
| Chromate of potass, properties of | 20 | 507 |
| Chronic peritonitis | 1 | 202 |
| Chronic diseases, percussion in | 2 | 70 |
| Chronic enlargement of the tonsils | 2 | 217 |
| Chronic indigestion, progress and symptoms of | 6 | 35 |
| Chronic gastritis, M. Andral on | 6 | 145 |
| Chronic diarrhœa, Dr. Elliotson on | 7 | 155 |
| Chronic rheumatism, treatment of | 7 | 352 |
| Chronic enlargement of the testicle, | 7 | 529 |
| Chronic enteritis, the rage in France, | 8 | 47 |
| Chronic peritonitis, some well-marked cases of | 8 | 71 |
| Chronic affections of the brain, Dr. Elliotson on | 8 | 448 |
| Chronic inflammation of the testicle, case of | 9 | 237 |
| Chronic inflammation, Mr. Scott's work on | 9 | 307 |
| Chronic enlargements of the joints, treatment of | 9 | 309 |
| Chronic affections of the joints | 10 | 178 |
| Chronic hepatitis, hysteria mistaken for | 12 | 240 |
| Chronic arachnitis, supposed case of, | 13 | 169 |
| Chronic inflammation of arteries | 13 | 353 |
| Chronic disease of the testis | 13 | 393 |
| Chronic phlegmasiæ, history of the | 17 | 151 |
| Chronic ophthalmia, case of | 17 | 166 |
| Chronic diseases, use of whey in | 20 | 197 |
| Chyle in the urine, case of | 9 | 109 |
| Chymification, derangements of | 14 | 438 |
| Cicatrices of burns, excision of | 4 | 553 |
| Cicatrix, excision of, in hydrophobia, | 7 | 236 |
| Cicatrix from a burn, removal of | 20 | 249 |
| Cicatrization, new mode of inducing, | 4 | 283 |
| Cicatrization of burns | 19 | 77 |
| Cicuta, poisoning by | 1 | 505 |
| Cinchona, salts of | 1 | 51 |
| Cinchonæ cordifoliæ, Battley's liquor, | 11 | 484 |
| Cinchonine, sulphate of, its employment | 5 | 283 |
| Circular swing in insanity, its powers | 7 | 29 |
| Circulating function, Dr. Uwins's ideas of | 2 | 385 |
| Circulating system, disease of the | 15 | 449 |
| Circulating organs, on the presence of air in | 18 | 517 |
| Circulation in the veins, Dr. Barry's theory | 4 | 488 |
| Circulation of the blood, Dr. Barry's researches on the | 5 | 313 |
| Circulation, new theory of the | 9 | 504 |
| Circulation in the lungs, Mr. Malden on the | 15 | 252 |
| Circulation of the blood | 19 | 247 |
| Cities, medical statistics of | 11 | 426 |
| City of London Med.-Chir. Society | 14 | 192 |
| Civilization, its influence on human life | 5 | 265 |
| Civilized and savage life contrasted | 3 | 190 |
| Clark, Mr. on the carb. ferri | 13 | 571 |
| Clark, Dr. on change of air | 16 | 456 |
| Clarke, Dr. on the fever of Smyrna, | 5 | 637 |
| Classification of poisons | 1 | 317 |
| Classification of cutaneous diseases | 2 | 45 |
| Classification of venereal diseases | 3 | 443 |
| Classifications of diseases | 16 | 382 |
| Classifications in botany | 17 | 395 |
| Clavicle excised for osteo-sarcoma | 10 | 549 |
| Clavicle, dislocation of, forwards | 12 | 178 |
| Clavicle, case of dislocation of | 18 | 165 |
| Clavicle, excision of | 19 | 501 |
| Cleft palate, operation unsuccessful, | 7 | 522 |
| Clement, Mr. on surgery & pathology, | 17 | 43 |
| Clerk, Dr. his answer to Tommasini, | 1 | 166 |
| Clerkships and house-surgeoncies, should be gratuitous | 18 | 562 |
| Cleverly, Dr. his death | 2 | 256 |
| Climate of Barbadoes—consumption, | 10 | 207 |
| Climate, Dr. Clark on | 11 | 121 |
| Climate, Dr. Clark on | 12 | 90 |
| Climate, Dr. Clark on | 13 | 77 |
| Climate of the Neilgherry | 13 | 308 |
| Climate of Malta | 14 | 336 |
| Climate, effects of, on fecundity | 20 | 162 |
| Climates, have they changed? | 3 | 318 |
| Clinical instruction in France and Germany | 4 | 216 |
| Clinical report of M. Lisfranc | 6 | 189 |
| Clinical observations by Dr. Bland | 6 | 238 |
| Clinical medicine, Dr. Clarke's pamphlet on | 6 | 580 |
| Clinical assistants | 7 | 284 |
| Clinical medicine, remarks on | 11 | 201 |
| Clinical Review, preface to the | 14 | 155 |
| Clinical reports, Mr. Macfarlane's | 17 | 515 |
| Clinical reports, Dr. Macfarlane's | 18 | 126 |
| Clinical remarks on opium, by Dr. Stokes | 18 | 156 |
| Clinical instruction, hints on | 18 | 226 |
| Clinical lectures of Dupuytren | 18 | 289 |
| Clinical reporting, remarks on | 18 | 560 |
| Clinical lectures, remarks on | 18 | 561 |
| Clinical lectures of Dr. Stokes | 20 | 257 |
| Clinique Medicale, by M. Andral | 3 | 493 |
| Clinique of the Hôtel Dieu | 6 | 531 |
| Clinique chirurgicale, M. Larry's | 15 | 1 |
| Clinique of Boyer and Roux | 15 | 521 |
| Clinique of Dupuytren | 18 | 530 |
| Clinique of M. Bouillaud | 18 | 533 |
| Clinique of M. Dupuytren | 20 | 241 |
| Clinique of M. Ricord | 20 | 245 |
| Clinique of M. Bouillaud | 20 | 254 |
| Clitoris, extirpation of | 3 | 558 |
| Clitoris and nymphæ, morbid enlargements of | 18 | 448 |
| Cloathing of infants, cautions respecting | 3 | 489 |
| Cloquet's anatomy, translated by Dr. Knox | 11 | 117 |
| Cloquet on the fascia transversalis | 19 | 471 |

| | Vol. | Page |
|---|---|---|
| Clot Bey on the Guinea-worm .... | 18 | 483 |
| Clot Bey on elephantiasis.......... | 18 | 529 |
| Clot Bey's School of Medicine at Abouzabel.................. | 18 | 545 |
| Clot Bey, M. on the Egyptian school, | 19 | 193 |
| Clot Bey, M. on the cholera ...... | 19 | 222 |
| Clot Bey, M. on lithotomy ........ | 19 | 489 |
| Clouds, Dr. Thompson on ........ | 13 | 475 |
| Clubs for suicide !................ | 11 | 430 |
| Clutterbuck's, Dr. theory of fever .. | 5 | 1 |
| Coagulation of the blood, Dr. Scudamore on.................... | 1 | 137 |
| Coagulation of the blood, in aneurismal sacs.................. | 13 | 361 |
| Coimbatoor;—climate of the ...... | 13 | 308 |
| Colchicum, fatal effects of ........ | 1 | 53 |
| Colchicum, Dr. Kinglake on ...... | 1 | 224 |
| Colchicum, in tape-worm ........ | 1 | 232 |
| Colchicum in rheumatism ........ | 2 | 430 |
| Colchicum, gathering of the flowers, | 3 | 573 |
| Colchicum in gout, Dr. Scudamore on.................... | 4 | 44 |
| Colchicum, various preparations of, | 4 | 46 |
| Colchicum, effects of, on the urine.. | 10 | 192 |
| Colchicum in cholera ............ | 15 | 205 |
| Cold, on the fatal effects of, on two people .................... | 1 | 265 |
| Cold water, affusions of, in derangement.................... | 3 | 110 |
| Cold affusion in poisoning by laudanum .................... | 3 | 276 |
| Cold, M. Beaupré on its effects .... | 5 | 77 |
| Cold, theory of.................. | 5 | 79 |
| Cold, its medicinal properties ...... | 5 | 89 |
| Cold and the warm bath conjointly in convulsions .............. | 5 | 159 |
| Cold water for mental hallucinations, | 9 | 203 |
| Cold air in pulmonary diseases...... | 10 | 559 |
| Cold lotions to the chest .......... | 13 | 153 |
| Cold affusion, M. Recamier on .... | 13 | 462 |
| Cold, its action on the lungs ...... | 14 | 147 |
| Cold, in poisoning by laudanum.... | 15 | 327 |
| Cold, Dr. Clendinning on ........ | 18 | 200 |
| Cold, prevention of diseases from .. | 18 | 202 |
| Cold affusion in cerebral cases...... | 18 | 205 |
| Cold affusion in tetanus .......... | 18 | 526 |
| Cold-bath Prison, dreadful mortality in | 17 | 423 |
| Cold-taking, remarks on .......... | 14 | 312 |
| Coley, Mr. on ischuria vesicalis .... | 19 | 190 |
| Colic, inflation for .............. | 15 | 157 |
| Colica pictonum, its treatment by "les Pères de la Charité," .... | 3 | 364 |
| Colica pictonum, leeches in. ...... | 3 | 528 |
| Colica pictonum, salivation in...... | 4 | 198 |
| Colica pictonum and "dry belly-ache," distinguished.......... | 4 | 251 |
| Colica pictonum, counter-irritation in | 4 | 282 |
| Colica pictonum, its seat.......... | 5 | 298 |
| Colica pictonum, M. Andral on .... | 7 | 86 |
| Colica pictonum, supposed case of, in the Royal Infirmary........ | 7 | 92 |
| Colica pictonum, Dr. Ranque on.... | 7 | 474 |
| Colica pictonum, cured by tobacco fomentations and croton oil.... | 7 | 114 |
| Coliseum, reflections on .......... | 14 | 458 |
| Collateral circulation of arteries .... | 13 | 363 |
| College of Surgeons, bye-law of .... | 1 | 241 |
| College of Surgeons and its Members, | 4 | 581 |
| College of Surgeons, new regulations | 5 | 632 |
| College of Surgeons, new regulations of the .................. | 6 | 268 |
| College of Physicians ............ | 7 | 505 |
| College of Physicians, celebrated memorial to the ............ | 8 | 233 |
| College of Physicians *v.* Dr. Harrison | 8 | 248 |
| College of Physicians *v.* Dr. Harrison | 8 | 479 |
| Coll. of Surgeons, its last curriculum | 8 | 515 |
| College curriculum, examination of it | 8 | 545 |
| College of Physicians, steps taken by | 8 | 560 |
| College-conversazione—vaccine virus | 9 | 215 |
| College of Physicians, ancient statutes | 9 | 262 |
| College of Physicians *v.* Dr. Harrison | 9 | 453 |
| College of Surgeons—Mr. Lawrence's appointment........ | 9 | 550 |
| College of Surgeons of Edinburgh, regulations of the............ | 11 | 552 |
| College of Physicians, soirées of the, | 12 | 520 |
| College of Surgeons .............. | 13 | 232 |
| College of Surgeons of Dublin...... | 14 | 239 |
| College of Surgeons, bye-laws of the, | 15 | 278 |
| College of Physicians ; reform..... | 18 | 582 |
| College of Physicians, reform of .... | 19 | 566 |
| Colleges of Surgeons and Physicians, | 15 | 162 |
| Colles, Dr. on dissection wounds ... | 7 | 306 |
| Colles, Dr. on diseases of the anus .. | 14 | 218 |
| Collins, Dr. his case of eleven-months' pregnancy .......... | 5 | 212 |
| Collins, Dr. on laceration of uterus.. | 14 | 357 |
| Collyrium, acetate of lead, a ...... | 3 | 163 |
| Colon, cases of obstruction in the .. | 1 | 125 |
| Colon, cases of accumulations in the, | 2 | 489 |
| Colon, a case of stricture and perforation of.................. | 5 | 275 |
| Colon, Mr. Annesley on disorders of, | 12 | 138 |
| Colon, stricture of the sigmoid flexure of the.......... ...... | 12 | 425 |
| Colon, fatal stricture of the........ | 12 | 504 |
| Colon, stricture of sigmoid flexure of | 13 | 150 |
| Colon, cases of stricture of the .... | 13 | 228 |
| Colon, cancer of its lower portion .. | 14 | 172 |
| Colon, spasm of the.............. | 15 | 207 |
| Colon, accumulation in the........ | 15 | 498 |
| Colon, abnormal flexure of ........ | 20 | 200 |
| Colours, insensibility to . ........ | 17 | 472 |
| Coma from loss of blood .......... | 13 | 62 |
| Combe, Dr. on anemia............ | 1 | 295 |
| Combe, Mr. on phrenology........ | 14 | 321 |
| Combe, Dr. on mental derangement, | 15 | 564 |
| Combe, Dr. on mental derangement, | 16 | 423 |
| Combustion, spontaneous, case of .. | 5 | 286 |
| Commentaries on female diseases .. | 7 | 316 |
| Common iliac artery, ligature of the, | 8 | 472 |
| "Compact Œdema," M. Leger's Memoir on.................... | 4 | 205 |

| | Vol. | Page |
|---|---|---|
| Company of Apothecaries, regulations of the | 12 | 169 |
| Compendium of medical practice .. | 2 | 382 |
| Compliments from our Gallic neighbours | 4 | 284 |
| Compound fractures, Larrey's treatment of | 3 | 269 |
| Compound fracture of the cranium, two cases of | 7 | 195 |
| Compound fracture of the skull, without depression | 7 | 525 |
| Compound fracture of the thigh—mortification | 8 | 533 |
| Compound fracture, Dr. Young's mode of treating | 8 | 554 |
| Compound fracture of the skull, case | 9 | 445 |
| Compound fracture of the tibia and fibula | 10 | 157 |
| Compound fractures, treatment of .. | 10 | 262 |
| Compound fracture of skull, case of, | 11 | 234 |
| Compound fracture of the skull—trephining | 11 | 238 |
| Compound fracture of femur, case of, | 18 | 162 |
| Compound fracture of cranium, trephining in | 18 | 509 |
| Compression of the spinal marrow .. | 1 | 13 |
| Compression, its effect in ascites.... | 2 | 500 |
| Compression in poisoned wounds .. | 6 | 232 |
| Compression in articular inflammation | 8 | 156 |
| Conception, Mr. Ashwell on ...... | 10 | 102 |
| Conception without consciousness .. | 20 | 131 |
| Concretions in the intestines, M. Langier on | 4 | 303 |
| Concussion of the spinal marrow .. | 1 | 15 |
| Concussion and compression, Sir A. Cooper on | 1 | 466 |
| Concussion of the spine, Mr. Dundas's case of | 3 | 225 |
| Concussion, case of ............... | 4 | 555 |
| Concussion of the brain and nervous system | 6 | 105 |
| Concussion, protracted sleep from .. | 9 | 214 |
| Concussion, interesting case of .... | 9 | 446 |
| Concussion of the brain, case of .... | 13 | 233 |
| Concussion, secondary symptoms .. | 13 | 234 |
| Concussion, scalp-wound.......... | 13 | 235 |
| Concussion, remarks on .......... | 13 | 558 |
| Concussion, treatment of.......... | 13 | 561 |
| Concussion of the brain, case of.... | 14 | 278 |
| Concussion of the spine, cases of .. | 17 | 9 |
| Concussion of spine, consequences of, | 17 | 13 |
| Condiments considered ........... | 6 | 8 |
| Condylomata, venereal, lotion for .. | 20 | 204 |
| Confinement in distortion, injurious, | 3 | 374 |
| Congenital blindness removed...... | 5 | 622 |
| Congenital cataract, operation for .. | 11 | 474 |
| Congenital malformations of the heart | 16 | 68 |
| Congenital hernia, Mr. Adams on .. | 18 | 486 |
| Congenital enterocele ............ | 18 | 486 |
| Congenital encephalocele.......... | 18 | 487 |
| Congestion, pseudo, of the brain.... | 12 | 75 |
| Congestive fever in the sea ........ | 2 | 240 |
| Congestive form of yellow fever, sketch of the | 8 | 77 |
| "Congestive fever," Dr. Burne's remarks on | 9 | 125 |
| Conium maculatum, M. Fodéré on.. | 19 | 218 |
| Conolly, Dr. on insanity .......... | 13 | 289 |
| Consecutive dislocation of the femur, | 3 | 55 |
| Consolations in travel ............ | 12 | 401 |
| Constantinople, curious singularity of the plague at | 5 | 237 |
| Constantinople, practice of physic in, | 11 | 142 |
| Constipation, fatal in pregnancy.... | 1 | 233 |
| Constipation, Dr. Crampton's case of | 7 | 118 |
| Constipation of the bowels, extraordinary case of | 12 | 181 |
| Constipation, fatal case of......... | 17 | 530 |
| Constitution, state of, amongst our students | 5 | 375 |
| Constitutional irritation from injuries | 3 | 388 |
| Constitutional syphilitic symptoms.. | 3 | 479 |
| Constitutional irritation, rationale of | 4 | 105 |
| Constitutional irritation, Mr. Travers on | 5 | 95 |
| Constitutional irritation, Mr. Travers on | 6 | 101 |
| Constitutional irritation from injuries | 6 | 115 |
| Constitutional irritation from inflammation | 6 | 117 |
| Constitutional irritation from loss of blood | 6 | 120 |
| Constitutional irritation from animal poison | 6 | 121 |
| Consultation, melancholy case for .. | 9 | 435 |
| Consumption, position in.......... | 3 | 497 |
| Consumption in France, fearful prevalence of | 4 | 493 |
| Consumption—abominable quackery | 9 | 533 |
| Consumption—St. John Long..... | 10 | 245 |
| Consumption, St. John Long's *cures*, | 11 | 287 |
| Consumption, Dr. Parrish on ...... | 12 | 241 |
| Consumption, Dr. Blackmore on.... | 17 | 400 |
| Contagion, definition of .......... | 2 | 389 |
| Contagion, arguments of its opponents | 3 | 67 |
| Contagion believed in by very ancient authors | 3 | 578 |
| Contagion and infection, their ultimate identity | 5 | 330 |
| Contagion, specific, does it exist after death? | 5 | 378 |
| Contagion, means of guarding against | 8 | 64 |
| Contagion of plague.............. | 14 | 190 |
| Contagion of cholera, M. Chervin on, | 15 | 500 |
| Contagion, Dr. Esquirol on........ | 17 | 183 |
| Contagion, fundamental doctrine of, | 17 | 239 |
| Contagion, Dr. Ferguson on ...... | 17 | 465 |
| Contagion, fear of................ | 18 | 148 |
| Contagion of diseases ............ | 18 | 187 |
| Contagionists, veracity of.......... | 18 | 271 |
| Contagious glanders, cure of ...... | 2 | 249 |
| Contagious psoriasis, supposed instances of | 7 | 116 |
| Contagious ophthalmia .......... | 14 | 403 |

| | Vol. | Page |
|---|---|---|
| Contagious fever at Chatham in 1813, | 15 | 561 |
| Contagiousness, acquired, of fever .. | 15 | 562 |
| Contracted joints, shampooing in .. | 3 | 382 |
| Contracted vagina, remarkable case, | 3 | 420 |
| Contracted mitral valve, diagnosis of, | 12 | 561 |
| Contraction of the anus .......... | 2 | 283 |
| Contraction of muscles in curved spine. | 3 | 369 |
| Contraction of the chest, some instances of .................. | 4 | 563 |
| Contraction of the limbs on suppression of menses .......... | 9 | 151 |
| Contraction of the chest, case of.... | 13 | 515 |
| Contusions from gunshot.......... | 18 | 579 |
| Convalescent retreats for the poor .. | 16 | 82 |
| Conversations on the "physiological doctrine"................ | 5 | 1 |
| Conversion of disease, Dr. Crampton, | 2 | 197 |
| Convulsion from loss of blood...... | 13 | 60 |
| Convulsion, peculiar, Dr. M. Hall on | 14 | 366 |
| Convulsions peculiar to children.... | 2 | 493 |
| Convulsions during labour ........ | 3 | 427 |
| Convulsions from loss of blood.. ... | 5 | 119 |
| Convulsions of infants, Mr. North on | 5 | 149 |
| Convulsions during or after delivery, | 10 | 108 |
| Convulsions, Dr. Bright on ........ | 16 | 343 |
| Convulsions, puerperal, Dr. Ramsbotham on.................. | 17 | 304 |
| Convulsions, puerperal, cases of.... | 17 | 305 |
| Convulsions, cold affusion in ...... | 17 | 459 |
| Convulsive epidemic............... | 13 | 571 |
| Convulsive croup, article on ...... | 14 | 366 |
| Convulsive disease, curious case of .. | 19 | 220 |
| Cooke, the tragedian, dissection of.. | 2 | 61 |
| Cooke, Mr. *persecution* of ........ | 7 | 286 |
| Cooke, Mr. on angina pectoris...... | 9 | 153 |
| Cooke, Mr. on the profession ...... | 15 | 526 |
| Cookery, its effects on different aliments.................... | 6 | 6 |
| Cool air, breathing, in pulmonary diseases .................... | 9 | 484 |
| Coombe, Mr. his system of phrenology | 5 | 437 |
| Cooper, Sir A. his lectures on surgery | 4 | 98 |
| Cooper, Sir A. his lectures ........ | 4 | 397 |
| Cooper, Sir A. on disease of the nails | 7 | 181 |
| Cooper *versus* Wakley ............ | 10 | 268 |
| Cooper Sir A. on diseases of breast, | 10 | 381 |
| Cooper, Mr. Sam. his edition of the study of medicine............ | 11 | 91 |
| Cooper, Mr. Bransby, his anatomy.. | 12 | 95 |
| Cooper, Sir Astley, on the testis.... | 13 | 385 |
| Cooper, Sir Astley, on the testis.... | 14 | 1 |
| Cooper's, Bransby, lectures........ | 14 | 140 |
| Cooper, Sir Astley, on the testis.... | 14 | 375 |
| Cooper, Mr. S. his case of aneurism, | 15 | 508 |
| Cooper, Mr. on anatomy.... ..... | 16 | 239 |
| Cooper, Mr. Bransby, his ligature of the iliac.................. | 16 | 514 |
| Cooper, Sir A. on the thymus gland, | 17 | 139 |
| Cooper, Mr. B. on anatomy........ | 18 | 97 |
| Cooper, Sir A. elected a member of the French Institute........ | 19 | 232 |
| Cooper, B. surgical essays ........ | 20 | 137 |
| Cooper, B. on wounds of the abdomen, | 20 | 139 |
| Cooper, B. on rupture of the jejunum, | 20 | 142 |
| Cooper, B. on rupture of the spleen.. | 20 | 144 |
| Cooper, B. on injuries of the head .. | 20 | 556 |
| Copaiba, balsam of, its properties .. | 3 | 151 |
| Copaiba in gonorrhœa, formula for, | 3 | 152 |
| Copland's, Dr. dictionary of medicine, | 18 | 111 |
| Copland, Dr. his Dictionary of Practical Medicine .......... | 20 | 46 |
| Copland, Dr. on diarrhœa ........ | 20 | 47 |
| Copper, sugar an antidote to ...... | 18 | 528 |
| Copper-plate printers, diseases of .. | 18 | 104 |
| Cord, encysted hydrocele of the .... | 12 | 269 |
| Cord, diffused hydrocele of the .... | 17 | 529 |
| Cord, inflamed, mistaken for hernia, | 18 | 468 |
| Corfu, climate, &c. of ............ | 14 | 97 |
| Corfu, diseases in ................ | 14 | 101 |
| Cormic, Mr. on cholera of India .... | 1 | 481 |
| Cornaro—should we all live like him? | 9 | 217 |
| Cornea and sclerotica, malformation, | 15 | 159 |
| Cornea, interstitial abscess of the .. | 17 | 276 |
| Cornea, ulcer of the.............. | 17 | 277 |
| Cornea, small-pox pustule on the .. | 19 | 257 |
| Cornea, deposition, interlamellar, of, | 19 | 448 |
| Cornea, ulceration of ............ | 19 | 448 |
| Corneitis, scrofulous.............. | 14 | 421 |
| Corns, Mr. Lawrence on .......... | 13 | 438 |
| Coronary arteries, ossification of the, | 9 | 158 |
| Coroner's inquest at St. George's .. | 3 | 583 |
| Coroner, duties of the............ | 8 | 504 |
| Coroner for Middlesex ............ | 13 | 573 |
| Coroner's inquest on St. John Long, | 13 | 521 |
| Corpora lutea found in a child five years old .................. | 3 | 588 |
| Corpora lutea, Dr. Montgomery on.. | 20 | 127 |
| Corpora lutea, their anatomical characters .................. | 20 | 128 |
| Corpora lutea, produced by impregnation .................... | 20 | 129 |
| Corporation laws, are they favourable to talent? .............. | 8 | 37 |
| Corporation monopolies .......... | 11 | 196 |
| Corporations for promoting science.. | 15 | 163 |
| Corrigan, Dr. on the heart ........ | 13 | 122 |
| Corrigan, Dr. on spinal irritation ... | 15 | 182 |
| Corrigan, Dr. his theory looking foolish | 15 | 220 |
| Corrigan, Dr. on dothinenterite .... | 16 | 227 |
| Corrigan, Dr. on sounds of the heart, | 20 | 134 |
| Corruption in medical charities .... | 20 | 186 |
| Corvisart, on percussion .......... | 2 | 65 |
| Costal abscess communicating with the bronchiæ................ | 3 | 213 |
| Coste, Dr. on embryology ........ | 19 | 527 |
| Costello, Mr. on lithotrity ........ | 13 | 257 |
| Cough from elongation of the uvula, | 9 | 206 |
| Cough, intermittent, cure of ...... | 19 | 217 |
| Coughing, fracture of rib from .... | 19 | 441 |
| Counteraction as a means of cure .. | 18 | 170 |
| Counter-irritation in diseases of heart | 13 | 535 |
| Counter-irritation, remarks on .... | 17 | 415 |
| Countries, medical statistics of .... | 11 | 425 |
| Coup-de-soleil, Mr. Mitchell on .... | 8 | 486 |

| | Vol. | Page |
|---|---|---|
| Coventry, Dr. his remarks on the water of the Mohawk valley, and its influence on goitre | 3 | 229 |
| Cowper, the poet, his character | 19 | 323 |
| Cow-pox, artificial production of | 20 | 208 |
| Cow-pox, origin of | 20 | 504 |
| Cradles, their often dangerous effects | 3 | 491 |
| Craigie, Dr. on whitlow | 9 | 170 |
| Cramp of the stomach, Dr. Macfarlane on | 11 | 489 |
| Crampton, Dr. on tinea | 1 | 359 |
| Crampton, Dr. on chorea | 1 | 363 |
| Crampton, Dr. on conversion of disease | 2 | 197 |
| Crampton, Dr. on opium poisoning, | 2 | 235 |
| Crampton, Dr. on tubercles of the skin | 9 | 169 |
| Crampton, Mr. his ligature of the common iliac | 14 | 54 |
| Crampton, Dr. on melanosis | 14 | 363 |
| Crampton, Mr. on injuries of the head, | 17 | 507 |
| Crampton, Mr. on inflammation of brain, &c. | 18 | 237 |
| Crampton, Mr. on dislocations | 19 | 270 |
| Cranial fracture, bold depletion in | 6 | 566 |
| Cranial bones, peculiar affection of the | 8 | 522 |
| Cranial bones, disease & removal of, | 11 | 523 |
| Cranial temperament | 13 | 14 |
| Craniotomy, directions for its performance | 4 | 160 |
| Craniotomy, child lived after | 20 | 204 |
| Cranium and spine, fracture of the | 7 | 485 |
| Cranium, peculiar affection of the | 8 | 489 |
| Cranium, compound fracture of the, trephining | 10 | 586 |
| Cranium, caries of | 14 | 546 |
| Cranium, on suppuration in the | 15 | 536 |
| Cranium, on compound fracture of | 17 | 511 |
| Cranium, injuries of, during parturition | 19 | 211 |
| Cremation, Sir Thomas Brown on | 13 | 480 |
| Creosote, its medicinal properties | 20 | 497 |
| Cretinism, Dr. Johnson on | 14 | 452 |
| Criminal abortion, review on | 13 | 97 |
| Crocodile, circulation in the | 19 | 247 |
| Croton oil in West Indian yellow fever | 4 | 263 |
| Croton tiglium, new preparation of | 4 | 573 |
| Croton oil as a counter-irritant | 19 | 448 |
| Croton oil, internal use of | 20 | 255 |
| Croup, Dr. Jadelot's treatment of | 3 | 348 |
| Croup, Mr. Mackenzie on | 4 | 261 |
| Croup, Mr. Pretty on | 4 | 590 |
| Croup; exudation expelled from the larynx | 5 | 538 |
| Croup, Mr. Mackenzie on the pathology of | 6 | 289 |
| Croup, epidemic | 11 | 169 |
| Croup, Dr. Goodlad on | 15 | 241 |
| Croup, Dr. Walker on | 19 | 363 |
| Croup, Dr. Steinmetz on | 19 | 501 |
| Croup, M. Maingault on | 19 | 526 |
| Croup, use of tartar-emetic in | 20 | 212 |
| Crown Assurance Company, prospectus of | 2 | 556 |
| Cruelty, physiological, to animals | 3 | 198 |
| Cruveilhier, M. aneurismal pouch in the heart | 7 | 401 |
| Cruveilhier, M. his anatomie pathologique | 12 | 79 |
| Cruveilhier, M. on fungous tumors | 13 | 494 |
| Cruveilhier, M. on laryngitis | 16 | 591 |
| Cruveilhier, M. on pathological anatomy | 19 | 137 |
| Cruveilhier's pathological anatomy | 19 | 329 |
| Cruveilhier's Anatomie Pathologique, | 20 | 303 |
| Crystalline lens, its reproduction asserted | 4 | 303 |
| Cubebs, in gonorrhœa and rheumatism | 1 | 228 |
| Cubebs, volatile oil of | 2 | 532 |
| Cubebs, paralysis from | 9 | 168 |
| Cullen, Mr. on melanosis | 2 | 154 |
| Cullen, Dr. on bronchotomy | 8 | 496 |
| Cullerier, M. his treatment of syphilis | 3 | 105 |
| Cumin, Dr. on the peripneumonia of children | 9 | 67 |
| Cuming, Dr. on an affection of the mouth | 7 | 120 |
| Cunningham, Mr. on the treatment of syphilis | 1 | 173 |
| Cunningham, Mr. on retroversio uteri, | 20 | 156 |
| Cupping in poisoned wounds strongly recommended | 5 | 38 |
| Cupping, Mr. Knox on | 15 | 471 |
| Cupping, dry, on | 19 | 178 |
| Cupping-glass in poisoned wounds, its application | 5 | 331 |
| Cupping-glass for emptying abscess, | 13 | 565 |
| Cupping-glasses to poisoned wounds, | 4 | 589 |
| Cupping-glasses in poisoned wounds, | 9 | 506 |
| Cupping-glasses applied in a case of viper-bite | 12 | 175 |
| Curative effects of loss of blood | 13 | 323 |
| Cures, spontaneous, of dropsical ovaria | 20 | 206 |
| Curious case of dyspnœa | 3 | 219 |
| Curriculum, new, of the Apothecaries' Company | 10 | 189 |
| Curriculum of the Irish College | 12 | 446 |
| Curvature of spine, Dr. Jarrold on | 1 | 303 |
| Curvatures of the spine, essay on | 2 | 112 |
| Cusack, Mr. on amputation of the jaw | 7 | 289 |
| Cusack, Dr. his report | 15 | 21 |
| Cut throat, Mr. Haden on | 1 | 132 |
| Cut-throat—curious symptoms of cerebral disturbance | 10 | 172 |
| Cut-throat, case of — abscess in front of the windpipe | 10 | 575 |
| Cutaneous diseases, works on | 2 | 29 |
| Cutaneous disorders, treatment of, at the Hôpital St. Louis | 5 | 76 |
| Cutaneous medication, M. Lesieur on | 5 | 531 |
| Cutaneous diseases, Drs. Graves and Stokes on | 7 | 479 |
| Cutaneous diseases, Drs. Graves & Stokes on | 8 | 465 |
| Cutaneous diseases, severe, on | 14 | 477 |

| | Vol. | Page |
|---|---|---|
| Cuticle, anatomy and functions of .. | 2 | 30 |
| Cuvier, last illness and memoir of .. | 17 | 451 |
| Cyanosis, on ..................... | 19 | 223 |
| Cyanuret of potassium in neuralgia.. | 18 | 525 |
| Cyclopædia of Medicine, Part I..... | 16 | 445 |
| Cyclopædia of Practical Medicine, Part III.................... | 16 | 598 |
| Cyclopædia of Practical Medicine .. | 17 | 246 |
| Cyclopædia of Practical Medicine .. | 18 | 362 |
| Cynanche trachealis .............. | 2 | 181 |
| Cynanche trachealis, observations on, | 6 | 380 |
| Cynanche trachealis, blood-letting in, | 6 | 382 |
| Cynanche trachealis, bronchotomy in | 6 | 383 |
| Cynanche laryngea; tracheotomy successful .................. | 7 | 122 |
| Cynanche trachealis, Dr. Mills on .. | 11 | 76 |
| Cynanche trachealis, cases of ...... | 12 | 218 |
| Cynanche tonsillaris.............. | 13 | 260 |
| Cynanche, fatal case of .......... | 14 | 171 |
| Cynanche, laryngea, Mr. Robertson, | 20 | 155 |
| Cyst, ovarian, injection of the...... | 12 | 427 |
| Cyst communicating with stomach.. | 15 | 568 |
| Cyst in the orbit, containing hydatids, | 18 | 342 |
| Cystitis hepatica, fatal instance of .. | 3 | 222 |
| Cystocele, long mistaken case of.... | 12 | 444 |
| Cystocele, fatal case of. .......... | 17 | 228 |
| Cystocele, vaginal, case of ........ | 18 | 208 |
| Cysts on the convex surface of liver, | 11 | 535 |
| Cysts, simple serous, characters of.. | 13 | 45 |
| **D** | | |
| Dance, M. on intus-susception ..... | 6 | 176 |
| Dance, M. on some remarkable cures, | 19 | 228 |
| Dancing, Mr. Sheldrake on ........ | 10 | 489 |
| Dandy fever ...................... | 9 | 213 |
| Dandy fever ...................... | 10 | 205 |
| Danger of suppressing habitual evacuations.................... | 13 | 467 |
| Dangerous symptoms after drinking porter ................. | 10 | 530 |
| Dangers of iodine ................ | 2 | 233 |
| Dangers of operations for deformities | 13 | 505 |
| Dantzig, cholera at .............. | 16 | 305 |
| Darwall, Dr. on cerebral and spinal irritation.................. | 11 | 433 |
| Darwall, Dr. on a peculiar paralysis, | 14 | 249 |
| Darwall, Dr. death of ............ | 19 | 569 |
| Datura stramonium in nervous complaints................... | 2 | 491 |
| Davids, Mr. case of wounded intestine | 20 | 182 |
| Davies, Mr. his case of phlegmatia dolens .................... | 3 | 540 |
| Davies, Mr. lunatic commission on.. | 12 | 433 |
| Davis, Dr. his essay on phlegmatia dolens .................... | 1 | 378 |
| Davis, Dr. his elements of midwifery, | 3 | 396 |
| Davis, Dr. his improved forceps .... | 3 | 408 |
| Davis, Dr. on the forceps.......... | 3 | 598 |
| Davis, Dr. on operative midwifery .. | 4 | 148 |
| Davis, Dr. on the ergot of rye...... | 4 | 276 |
| Davis, Dr. on perforation of the stomach...................... | 9 | 465 |
| Davis, Mr. on the ergot of rye...... | 11 | 480 |
| Davis, Dr. on obstetric medicine.... | 16 | 235 |
| Davis, Dr. on obstetric medicine.... | 16 | 579 |
| Davis, Dr. his obstetric medicine.... | 18 | 491 |
| Davis, Dr. D. on hysteralgia........ | 19 | 162 |
| Davis, Dr. D. on hysteria.......... | 19 | 190 |
| Davis, Dr. D. on nymphomania .... | 19 | 465 |
| Davis, Dr. D. on sterility.......... | 20 | 159 |
| Davy, Dr. on anatomical preparations | 9 | 404 |
| Davy's, Sir H. consolations in travel, | 12 | 401 |
| Davy, Sir Humphry, life of........ | 14 | 423 |
| Dawson's, Dr. practice of physic.... | 2 | 382 |
| Dead body, prosecution for *possessing* | 9 | 161 |
| Deafness in old people ............ | 3 | 299 |
| Deafness, iodine in .............. | 4 | 13 |
| Deafness, Mr. Stevenson on........ | 10 | 45 |
| Death, sudden, in an operation .... | 2 | 218 |
| Death from epileptiform attacks.... | 3 | 218 |
| Death after forceps operations...... | 4 | 157 |
| Death from excessive depletion; melancholy case ............ | 4 | 355 |
| Death, sudden, from peritoneal inflammation ................ | 5 | 276 |
| Death of Dr. Gall................ | 10 | 190 |
| Death from stricture .............. | 10 | 204 |
| Death from loss of blood .......... | 14 | 491 |
| Death from dread of operation .... | 15 | 175 |
| Death of Dr. Allsop .............. | 17 | 574 |
| Death from lithotomy, cases and remarks.................... | 18 | 564 |
| Death, black, Dr. Babington on .... | 19 | 121 |
| Death from severe injuries ........ | 19 | 195 |
| Debility, Dr. Shearman on ........ | 1 | 391 |
| Debility a cause of hysteria ... .... | 13 | 31 |
| Debility, remarks on.............. | 13 | 314 |
| Decayed teeth, curious cases of irritation from................ | 4 | 101 |
| Defamation of character .......... | 1 | 511 |
| Defecation, Dr. O'Beirne on ....... | 19 | 1 |
| Defect of power in arteries, a cause of predisposition .......... | 3 | 197 |
| Definitions of contagion and infection | 3 | 68 |
| Delirium during labour, bleeding in, | 3 | 423 |
| Delirium tremens, speculations on .. | 4 | 37 |
| Delirium tremens, cases of ........ | 4 | 38 |
| Delirium tremens, Mr. Playfair's paper on .................... | 5 | 40 |
| Delirium tremens, Dr. Coates on.... | 8 | 457 |
| Delirium tremens, discussion on.... | 8 | 484 |
| Delirium, treated by mercury...... | 8 | 503 |
| Delirium tremens, memoir on...... | 9 | 509 |
| Delirium tremens, treatment of .... | 10 | 16 |
| Delirium traumaticum—effects of opium .................... | 10 | 525 |
| Delirium, Dr. Abercrombie on...... | 12 | 197 |
| Delirium tremens, Dr. Weir on .... | 12 | 568 |
| Delirium from loss of blood........ | 13 | 61 |
| Delirium tremens, Dr. Blake on .... | 13 | 196 |
| Delirium tremens, Dr. B. Pearson on, | 13 | 198 |
| Delirium traumaticum, fatal case .. | 15 | 272 |
| Delirium tremens, cases of ........ | 15 | 294 |
| Delirium tremens, case of.......... | 17 | 173 |

| | Vol. | Page |
|---|---|---|
| Delirium tremens, Dr. O'Beirne on.. | 19 | 26 |
| Delirium tremens, Dr. Stokes on.... | 20 | 457 |
| Delivery, sudden ................ | 1 | 496 |
| Delivery, immediate, in ruptured uterus ........ ........... | 4 | 24 |
| Delivery, without consciousness.... | 20 | 132 |
| Delpech's amputation at the hip-joint | 2 | 225 |
| Delpech, M. on elephantiasis ...... | 12 | 535 |
| Delpech, M. on cancer of the jaw .. | 13 | 546 |
| Delpech, M. on imperforate os uteri, | 13 | 553 |
| Delpech, M. on varicocele ........ | 16 | 252 |
| Delpech, M. assassination of ...... | 18 | 519 |
| Democritus, jun. on quackery ...... | 14 | 193 |
| Denman, Dr. his letter to Dr. Parry, | 13 | 479 |
| Denmark, Mrs. postcript to her case, | 8 | 281 |
| Denmark, Mrs. strange statements respecting ................. | 8 | 468 |
| Dental gangrene, causes of ........ | 11 | 330 |
| Depletion for traumatic gangrene .. | 9 | 237 |
| Depletion in dissection-wounds .... | 12 | 533 |
| Depositions of pus in the liver and lungs, Mr. Rose on .......... | 9 | 457 |
| Depositions in the coats of arteries.. | 13 | 352 |
| Depôt in one arm from scalp-wound, | 14 | 236 |
| Depôts in distant parts from erysipelas | 14 | 237 |
| Descot, Dr. on local affections of the nerves ................. | 6 | 434 |
| Determination to the brain, case of.. | 4 | 145 |
| Deuto-iodure of mercury in cutaneous affections.......... | 6 | 566 |
| Development of the embryo ....... | 20 | 508 |
| Devon, climate of its south-west coast | 11 | 126 |
| Devrient's, Mr. trans. of Hahnemann, | 19 | 429 |
| Dewees, Dr. on the secale cornutum, | 9 | 209 |
| Diabetes, Dr. Sharkey on.......... | 2 | 237 |
| Diabetes, venesection in .......... | 4 | 260 |
| Diabetes mellitus, animal diet in.... | 6 | 238 |
| Diabetes mellitus, M. Luroth's case.. | 7 | 496 |
| Diabetes mellitus, discussion ...... | 8 | 545 |
| Diabetes, M. Hufeland on ......... | 9 | 436 |
| Diabetes, cataracts alternating with, | 12 | 176 |
| Diabetes, Dr. Venables on ........ | 13 | 68 |
| Diabetes, morbid anatomy of ...... | 13 | 71 |
| Diabetes, treatment of............ | 13 | 73 |
| Diabetes, Dr. Bright on .......... | 16 | 9 |
| Diabetes, analysis of blood & urine in | 17 | 219 |
| Diabetes mellitus relieved ........ | 17 | 439 |
| Diabetes mellitus, case of.......... | 18 | 176 |
| Diabetes mellitus, case of.......... | 18 | 192 |
| Diagnosis of aortic aneurisms ...... | 1 | 183 |
| Diagnosis of valvular disease of the heart ..................... | 1 | 196 |
| Diagnosis & prognosis, M. Rostan on | 6 | 51 |
| Diagnosis of hernia, discussions on the | 8 | 575 |
| Diagnosis of hernia .............. | 12 | 450 |
| Diaphragm, supposed wounds of.... | 1 | 510 |
| Diaphragm, its action prevented by stays ..................... | 3 | 192 |
| Diaphragm and vena cava, rupture of | 9 | 280 |
| Diaphragm, neuralgia of the........ | 13 | 461 |
| Diarrhœa, inflammatory .......... | 1 | 112 |
| Diarrhœa after severe labours...... | 4 | 155 |
| Diarrhœa hectica, Mr. Tytler on.... | 10 | 399 |
| Diarrhœa, Dr. Copland on ........ | 20 | 47 |
| Diastasis of the vertebræ, case of.... | 3 | 44 |
| Diastasis of the epiphysis of femur .. | 3 | 49 |
| Diathesis, Rassori's observations on, | 1 | 222 |
| Diathesis, purulent, case of........ | 19 | 242 |
| Dickson, Dr. on sulphate of quinine, | 1 | 331 |
| Dickson, Dr. case of ascites........ | 20 | 177 |
| Dictionary of practical medicine, Dr. Copland's .............. | 18 | 111 |
| Dieffenbach on staphyloraphy...... | 5 | 188 |
| Dieffenbach, M. on restored vision.. | 19 | 158 |
| Diet, its effect on the Millbank prisoners .................... | 3 | 4 |
| Diet, certain *dicta* respecting, remarks on .................. | 5 | 103 |
| Diet, Dr. Paris's treatise on........ | 6 | 1 |
| Diet of phthisical patients ......... | 6 | 45 |
| Diet, abstemious, abuse of ... .... | 14 | 154 |
| Diet, Mr. Cameron on............ | 18 | 94 |
| Differences between yellow and remittent fever................ | 3 | 77 |
| Difficult labours, Mr. Ashwell on .. | 10 | 109 |
| Difficulties of anatomy in America .. | 13 | 499 |
| Diffuse inflammation, Dr. Duncan on | 2 | 335 |
| Diffuse inflammation after bleeding in foot ..................... | 18 | 462 |
| Diffuse cellular inflammation after lithotomy ................ | 18 | 565 |
| Diffused spurious aneurism, case of.. | 14 | 282 |
| Diffused aneurism, case of ........ | 14 | 488 |
| Digestion, curious experiments on .. | 6 | 510 |
| Digestion, important experiments on, | 8 | 209 |
| Digestion, new theory of.......... | 11 | 254 |
| Digestive organs in, and prior to, scrophula ................... | 3 | 483 |
| Digestive organs, nature of the sensibility of the............... | 5 | 27 |
| Digestive organs, influence of, on the mind .................... | 9 | 149 |
| Digitalis in insanity ............... | 10 | 23 |
| Digitalis, morbid effects of ........ | 15 | 496 |
| Digitalis, use of in some affections of the head ................. | 20 | 57 |
| Dilatation of the bronchial tubes .. | 3 | 217 |
| Dilatation of the bronchial tubes.... | 3 | 500 |
| Dilatation of the urethra, M. Dupuytren on ................. | 7 | 449 |
| Dilatation of the urethra in the female for stone ............. | 12 | 209 |
| Dilatation of arteries.............. | 13 | 356 |
| Dilator vaginæ found in Pompeii.... | 12 | 247 |
| Dill, Dr. his dissertation on cuticular absorption ............ | 5 | 240 |
| Dill, Dr. the late ................ | 17 | 285 |
| Dinner of the general practitioners .. | 12 | 518 |
| Dinners, medical, ancient and modern | 13 | 489 |
| Diplogenesis, monstrosity by ...... | 20 | 231 |
| Diploma of London University...... | 13 | 204 |
| Diptherite, M. Maingault on ...... | 19 | 234 |
| Diptherite, on the................ | 19 | 245 |
| Diptherite, M. Gendron on........ | 19 | 528 |

| | Vol. | Page |
|---|---|---|
| "Diptheritis," Dr. Bretonneau on .. | 5 | 419 |
| Discharge, hæmorrhoidal, nature of.. | 2 | 274 |
| Discharge of a fœtus from umbilicus, | 20 | 162 |
| Discharge, mucous, from the uterus, | 20 | 222 |
| Discolouration of the skin, from imperfect heart.............. | 11 | 159 |
| Discovery of the use of the spleen .. | 13 | 486 |
| Disease of the heart, Dr. Baker's case, | 3 | 529 |
| Disease, *morbid* generalizations of .. | 5 | 351 |
| Disease of the heart, Dr. Macmichael on.................. | 5 | 605 |
| Disease of the heart, remarkable cases of.................. | 5 | 623 |
| Disease & disorder—function & organ | 6 | 52 |
| Disease of the heart, very instructive case of .................. | 6 | 215 |
| Disease of the breast, two cases of .. | 8 | 126 |
| Disease of the heart, valvular, case of | 8 | 463 |
| Disease of the heart, Mr. Maynard's case of ............... | 8 | 474 |
| Disease of the lungs—remarkable case | 8 | 487 |
| Disease of the heart, Mr. Wishart's case | 9 | 483 |
| Disease, spread of................ | 12 | 300 |
| Disease, influence of, on the mind.. | 15 | 197 |
| Disease, appearances of the tongue in, | 15 | 243 |
| Disease transmitted along an electric wire .................. | 18 | 215 |
| Diseased and wounded arteries...... | 7 | 461 |
| Diseased joints, Mr. Buchanan on .. | 10 | 128 |
| Diseases of the teeth considered .... | 6 | 132 |
| Diseases of the hip-joint, cases of .. | 7 | 254 |
| Diseases of the brain, &c. Dr. Abercrombie on ................. | 8 | 337 |
| Diseases of the jaws, Mr. Koecker on | 8 | 425 |
| Diseases of brain, Dr. Abercrombie on | 9 | 73 |
| Diseases of infants, clinical report on | 9 | 234 |
| Diseases of the joints, Mr. Scott on, | 9 | 307 |
| Diseases of India, Mr. Annesley on.. | 9 | 337 |
| Diseases of the heart, Dr. Adams on, | 9 | 385 |
| Diseases of the stomach, Dr. Abercrombie on .................. | 10 | 328 |
| Diseases of the bones, Mr. B. Bell on | 10 | 405 |
| Diseases of the teeth, Mr. Bell on the, | 11 | 408 |
| Diseases of the heart, interesting cases of ................... | 11 | 517 |
| Diseases of the heart, instructive cases of .................... | 11 | 556 |
| Diseases of arteries, Mr. Guthrie on.. | 13 | 133 |
| Diseases of arteries, Mr. Guthrie on.. | 13 | 350 |
| Diseases of the chest, report on .... | 13 | 446 |
| Diseases of the lungs, report on .... | 13 | 513 |
| Diseases of the heart.............. | 13 | 535 |
| Diseases of the eye, Mr. Mackenzie.. | 14 | 397 |
| Diseases of the heart, Dr. Hope on.. | 16 | 262 |
| Diseases of the heart, their importance | 16 | 263 |
| Diseases of the spine, Mr. Stafford on | 17 | 1 |
| Diseases of women, Dr. R. Lee on .. | 19 | 58 |
| Diseases of urethra ............... | 19 | 107 |
| Diseases, organic, of the fœtus ..... | 19 | 140 |
| Diseases of Egypt.................. | 19 | 194 |
| Diseases of the liver ............... | 19 | 415 |
| Diseases of the muscles ........... | 19 | 512 |

| | Vol. | Page |
|---|---|---|
| Dislocated head of humerus........ | 13 | 464 |
| Dislocation of the vertebræ in curvature ..................... | 3 | 376 |
| Dislocation and fracture of the cervix femoris ............... | 4 | 543 |
| Dislocation of the cervical vertebræ, supposed case of ........ | 6 | 613 |
| Dislocation of head of humerus, under pectoral muscle ........ | 10 | 138 |
| Dislocation of the humeri undiscovered ................... | 10 | 142 |
| Dislocation of the radius backwards, | 10 | 177 |
| Dislocation into the ischiatic notch.. | 10 | 238 |
| Dislocation into the ischiatic notch, diagnosis of ............... | 10 | 251 |
| Dislocation, partial, of the knee .... | 10 | 582 |
| Dislocation of the patella outwards, curious cases of ............ | 11 | 145 |
| Dislocation of the cornua of the os hyoides ................... | 12 | 159 |
| Dislocation of the clavicle ........ | 12 | 178 |
| Dislocation of the head of the radius backwards............ | 12 | 178 |
| Dislocation, congenital, of radius .. | 13 | 464 |
| Dislocation, compound, of the os naviculare ................. | 15 | 160 |
| Dislocation of the clavicle.......... | 18 | 165 |
| Dislocation of the humerus........ | 18 | 165 |
| Dislocation of the shoulder, Mr. Crampton on .............. | 19 | 270 |
| Dislocation of the hip, with fracture, | 19 | 480 |
| Dislocation of the humerus backwards | 19 | 503 |
| Dislocations of the vertebræ, Mr. Lawrence on ............... | 8 | 320 |
| Dislocations of clavicle, remarks on, | 9 | 526 |
| Dislocations of the astragalus ...... | 20 | 520 |
| Disorder of the general health in females, chronic form ........ | 7 | 324 |
| Disorders, mental, Dr. Uwins on.... | 20 | 49 |
| Disorganization of the stomach; Dr. Gairdner's case .......... | 6 | 86 |
| Dispensary patients, a picture of.... | 16 | 81 |
| Dissecting, slovenly mode of, in the French school .............. | 4 | 215 |
| Dissecting-room, pathology in the.. | 8 | 542 |
| Dissecting aneurism, description of.. | 13 | 355 |
| Dissection-wounds, Mr. Shaw on .. | 3 | 249 |
| Dissection-wounds, Dr. Thomson's own case ................... | 3 | 253 |
| Dissection-wounds, their effects on the system................. | 3 | 254 |
| Dissection-wounds, American practice in .................... | 3 | 300 |
| Dissection, very able, of a hydrophobic patient .............. | 3 | 534 |
| Dissection-wounds treated by lunar caustic ................... | 4 | 383 |
| Dissection-wounds, distinctions of .. | 4 | 384 |
| Dissection-wound, death from...... | 5 | 364 |
| Dissection-wound, severe, case of .. | 5 | 371 |
| Dissection-wound, fatal case of .... | 5 | 615 |
| Dissection-wound, Mr. Shaw's case of | 6 | 546 |

| | Vol. | Page |
|---|---|---|
| Dissection-wound, fatal case of .... | 7 | 306 |
| Dissection-wound, incisions in .... | 9 | 174 |
| Dissection-injury—not *wound*...... | 9 | 215 |
| Dissection-wound ................ | 9 | 449 |
| Dissection-wound, rapidly fatal ... | 9 | 488 |
| Dissection-wound, case of ........ | 12 | 522 |
| Dissection-wounds, Mr. Lawrence on | 12 | 529 |
| Dissection of the dead, report on.... | 16 | 101 |
| Dissections, advantage of chloruret of lime in ................ | 6 | 354 |
| Dissolution of calculi by galvanism.. | 1 | 223 |
| Distended bladder; Mr. Thompson's case ................ | 5 | 507 |
| Distention of the duodenum........ | 7 | 366 |
| Distention of intestines .......... | 13 | 151 |
| Distinction without separation .... | 15 | 161 |
| Distortion, does it begin in the loins or back?.................... | 3 | 368 |
| Distortion of the face cured by disease of the brain! ............ | 12 | 264 |
| Distortions of the spine, Mr. Bampfield on .................... | 2 | 112 |
| Distortions of the spine, Mr. Beale on | 15 | 418 |
| Disturbance of speech, curious case of | 3 | 526 |
| Division of the spinal marrow, in the dorsum ................ | 9 | 442 |
| Dix, Mr. his case of wounded abdomen | 4 | 549 |
| Dobson, Dr. on punctures in erysipelas | 9 | 382 |
| Dobson, Mr. on the spleen ........ | 13 | 531 |
| Dobson, Mr. on the epidemic cholera at Leeds ................ | 16 | 636 |
| Doctors; shewing how at times they disagree ................ | 4 | 441 |
| Doctors deceived ................ | 18 | 231 |
| Doctrine homoiopathique........ | 17 | 132 |
| Dodd, Mr. on cholera ............ | 16 | 576 |
| Dods, Dr. A. on contorted spine .... | 1 | 92 |
| Domesticated animals, their maladies | 3 | 195 |
| Donellan's case, Mr. Hunter's evidence in .................... | 3 | 183 |
| Donnall's trial for poisoning Mrs. Downing .................. | 3 | 183 |
| Dorsal vertebræ, fracture and depression of the .............. | 8 | 176 |
| Dorsal vertebræ, removal of ...... | 12 | 207 |
| Doses, large, of tartarised antimony, | 3 | 106 |
| Dothinenterite, Dr. Corrigan on .... | 16 | 227 |
| Dothinenterite, cases of .......... | 18 | 193 |
| Dothinenteritis—another "new theory of fever!" ............ | 5 | 199 |
| Double bladder, supposed case of .. | 13 | 160 |
| Dough *rises* when arsenic is mixed with it .................... | 3 | 181 |
| Dracunculus, mercurialization for .. | 5 | 43 |
| Drainage and tillage, their effects on health .................. | 8 | 20 |
| Drinks, different kinds of.......... | 6 | 9 |
| Dropsical diseases, pathology of .... | 8 | 92 |
| Dropsical diseases, treatment of .... | 8 | 101 |
| Dropsical effusion and liver disease.. | 8 | 313 |
| Dropsical ovaria, spontaneous cures of | 20 | 206 |
| Dropsy, Dr. Stoker on ............ | 1 | 144 |
| Dropsy in pregnancy, curious case of | 2 | 523 |
| Dropsy, Dr. Venables on .......... | 3 | 88 |
| Dropsy (encysted) of the liver, case of | 3 | 224 |
| Dropsy after hæmorrhages ........ | 4 | 134 |
| Dropsy, a curative process, case of.. | 4 | 135 |
| Dropsy, Drs. Ayre and Shearman on; | 4 | 406 |
| Dropsy, various kinds & treatment of | 7 | 62 |
| Dropsy with pregnancy .......... | 10 | 104 |
| Dropsy, iatraleptic treatment of. .. | 10 | 199 |
| Dropsy, encysted, of the ovaria .... | 12 | 419 |
| Dropsy, Dr. Billing's views of ...... | 14 | 395 |
| Dropsy, Dr. Elliotson on .......... | 14 | 511 |
| Dropsy of the membranes ........ | 17 | 312 |
| Dropsy of the uterus, case of ...... | 20 | 117 |
| Dropsy, use of iodine in .......... | 20 | 259 |
| Drowning, signs of .............. | 9 | 489 |
| Drowning, Sir Astley Cooper's experiments on.............. .. | 18 | 19 |
| Drug, Dr. on the etiology of goitre.. | 2 | 442 |
| Drunkards, state of the stomach in.. | 3 | 531 |
| Drunkenness, cured by ammonia .. | 1 | 229 |
| Drunkenness, prevention of........ | 1 | 480 |
| Dryden, Mr. his Report on the Dock-yard fever ............ | 4 | 66 |
| Dublin, regulations relating to medical degrees ................ | 11 | 71 |
| Dublin Fever Hospital, report from, | 13 | 408 |
| Dublin College of Surgeons........ | 14 | 239 |
| Dublin Medical Transactions ...... | 14 | 357 |
| Dublin Fever Hospital, report of .. | 18 | 149 |
| Ducamp, Dr. on retention of urine.. | 17 | 49 |
| Ducasse, M. his exposé of French surgeons .................. | 4 | 550 |
| Dufau, M. on intermittent irritations | 6 | 457 |
| Duffin, Dr. his new pessary ........ | 15 | 281 |
| Dugés, M. on diseases of the uterus, | 18 | 406 |
| Dugés, M. on the uterus .......... | 19 | 522 |
| Dumeril, M. his mystical collar .... | 3 | 358 |
| Dumfries meeting on cholera ...... | 18 | 216 |
| Duncan, Dr. on hydrocephalus .... | 1 | 297 |
| Duncan, Dr. on phlebitis .......... | 2 | 161 |
| Duncan, Dr. on diffuse inflammation, | 2 | 335 |
| Duncan, Dr. his case of cellular inflammation................ | 3 | 212 |
| Duncan, Dr. on empyema, &c....... | 8 | 131 |
| Duncan, Dr. his case of catalepsy .. | 13 | 250 |
| Duodenal indigestion, remarks on .. | 6 | 31 |
| Duodenum, *not quite* a second stomach .................... | 4 | 422 |
| Duodenum, perforation of the...... | 9 | 573 |
| Duodenum, diseases of, Dr. Bright on | 20 | 1 |
| Dupuytren, operations by.......... | 2 | 219 |
| Dupuytren, M. his tonic potion .... | 3 | 356 |
| Dupuytren, M. his powder for gangrene .................... | 3 | 357 |
| Dupuytren, M. his merits.......... | 3 | 363 |
| Dupuytren, M. his case of ligature of the subclavian ............ | 6 | 163 |
| Dupuytren, M. on congenital dislocation of the hip-joint ...... | 6 | 544 |
| Dupuytren, M. his case of ligature on the external iliac.......... | 7 | 177 |

| | Vol. | Page |
|---|---|---|
| Dupuytren, M. his mode of treating ranula | 9 | 226 |
| Dupuytren, M. on lateral depression of the thorax | 9 | 311 |
| Dupuytren, M. on artificial anus | 9 | 313 |
| Dupuytren, M. on fractures and dislocations | 10 | 133 |
| Dupuytren, M. on the right iliac fossa | 10 | 217 |
| Dupuytren foiled in lithotomy | 10 | 453 |
| Dupuytren, M. his case of ligature ultrà aneurisma | 11 | 469 |
| Dupuytren, M. on herniæ of the muscles | 11 | 566 |
| Dupuytren, M. his rhinoplastic operation | 12 | 268 |
| Dupuytren, M. on specks of the cornea | 12 | 539 |
| Dupuytren, M. his treatment of scrofula | 12 | 554 |
| Dupuytren, M. on wounded arteries, | 13 | 139 |
| Dupuytren, M. criticised by Mr. Guthrie | 13 | 142 |
| Dupuytren, M. on union by first intention | 13 | 190 |
| Dupuytren, M. on varicose aneurism, | 13 | 508 |
| Dupuytren's clinical lectures, review of | 18 | 289 |
| Dupuytren on permanent retraction of fingers | 18 | 290 |
| Dupuytren on cataract | 18 | 295 |
| Dupuytren on enlargements of the testis | 18 | 300 |
| Dupuytren on traumatic emphysema, | 18 | 302 |
| Dupuytren on fistulous sinuses | 18 | 303 |
| Dupuytren on prolapsus recti | 18 | 305 |
| Dupuytren on traumatic delirium | 18 | 306 |
| Dupuytren on fractures of fibula | 18 | 308 |
| Dupuytren on false aneurism of brachial artery | 18 | 315 |
| Dupuytren on fractures of patella | 18 | 316 |
| Dupuytren on fractures in general | 18 | 317 |
| Dupuytren on excision of hæmorrhoids | 18 | 318 |
| Dupuytren, clinical lectures of | 19 | 68 |
| Dupuytren, his lecons orales | 19 | 542 |
| Dupuytren on hydatid tumors of wrist | 20 | 241 |
| Dupuytren on club-feet | 20 | 242 |
| Dupuytren on encysted tumors | 20 | 242 |
| Dupuytren on syphilis and syphiloid diseases | 20 | 243 |
| Dura mater, tumor growing from | 11 | 512 |
| Dura mater, fungus of the | 13 | 496 |
| Dura mater, fungus of the | 17 | 483 |
| Dura mater, fungoid tumor of the | 20 | 526 |
| Duration of pregnancy, tables on the, | 8 | 232 |
| Dutrochet, M. sur l'agent immédiate de la vie | 11 | 49 |
| Dutrochet, sur l'endosmose et l'exosmose | 11 | 49 |
| Dysentery, Dr. O'Halloran's treatment of | 2 | 234 |
| Dysentery, employment of tobacco in | 2 | 495 |
| Dysentery, P. Recamier's ideas of | 3 | 220 |
| Dysentery in the Fly sloop of war | 3 | 270 |
| Dysentery, cases of, treated by the chloruret of lime | 8 | 42 |
| Dysentery & cholera, Dr. Macculloch on | 9 | 289 |
| Dysentery in the East | 11 | 327 |
| Dysentery, large doses of ipecacuan in | 14 | 332 |
| Dysentery, Mr. Annesley on | 15 | 102 |
| Dysentery, acute | 15 | 102 |
| Dysentery, hepatic | 15 | 105 |
| Dysentery, post-mortem appearances | 15 | 107 |
| Dysentery, treatment of | 15 | 111 |
| Dysentery, report on | 17 | 409 |
| Dysentery, Dr. O'Beirne on | 19 | 22 |
| Dysmenorrhœa, Dr. Campbell on. | 6 | 494 |
| Dysmenorrhœa cured by bougies! | 9 | 518 |
| Dysmenorrhœa | 13 | 336 |
| Dyspepsia, mode of treatment of | 4 | 43 |
| Dyspepsia, curious case of | 7 | 412 |
| Dyspepsia, Dr. Abercrombie on | 10 | 338 |
| Dyspepsia, Dr. Stokes on | 19 | 175 |
| Dyspepsia, Dr. Stokes on | 20 | 459 |
| Dysphagia, case of, by Dr. Hay | 1 | 300 |
| Dysphagia, case of, cured by iodine, | 4 | 15 |
| Dysphagia, some curious cases of | 6 | 236 |
| Dysphagia, treatment of | 19 | 149 |
| Dysphagia, from aneurism of the aorta | 20 | 261 |
| Dyspnœa, fatal cases of | 2 | 444 |
| Dyspnœa, chronic, case of | 6 | 254 |
| Dyspnœa cured by prussic acid | 9 | 173 |
| Dyspnœa, acute intermittent | 10 | 532 |
| Dyspnœa, spasmodic, cured by purging | 14 | 163 |

E

| | Vol. | Page |
|---|---|---|
| Ear, Mr. Buchanan, on the | 1 | 42 |
| Ear, supposed foreign body in the; reflections | 11 | 565 |
| Ear, internal, disease of the | 14 | 232 |
| Ear, inflammation of, in the insane. | 20 | 201 |
| Earle, Mr. H. on fractures | 1 | 58 |
| Earle, Mr. H. plate of his fracture-bed | 1 | 68 |
| Earle, Mr. his case of local irritation, | 6 | 199 |
| Earle, Mr. his cases of strangulated hernia | 7 | 472 |
| Earle, Mr. his case of hernia | 8 | 170 |
| Earle, Mr. on vesico-vaginal fistula. | 12 | 272 |
| Earle, Mr. his case of hæmorrhage. | 13 | 266 |
| Earle, Mr. on chimney-sweeper's cancer | 17 | 153 |
| Earle, Mr. on burns | 17 | 492 |
| East India Company, memorial to the, | 13 | 114 |
| Eastcott, Mr. on fatty discharge from the bowels | 19 | 477 |
| Easton, Mr. on spontaneous cholera, | 20 | 158 |
| Easton, Mr. on dysentery | 20 | 159 |
| Easton, Mr. on hæmatemesis | 20 | 159 |
| Easton, Mr. on ext. use of croton oil, | 20 | 159 |
| Eau bénite, formula for it | 3 | 356 |
| Eccentricity, curious examples of. | 13 | 292 |
| Eclampsia in infants | 20 | 240 |
| Eclectic review on peripneumony | 8 | 289 |
| Ecthyma cachecticum, case of | 19 | 242 |

| | Vol. | Page |
|---|---|---|
| Ectropion, new operation for | 13 | 566 |
| Ectropopharynx | 1 | 475 |
| Eczema rubrum, case of | 9 | 571 |
| Eczema and elephantiasis, M. Biett on | 11 | 570 |
| Eczema, M. Biett on | 16 | 386 |
| Eczema, treatment of | 16 | 390 |
| Edinburgh Med.-chir. Transactions | 1 | 285 |
| Edinburgh Senate, their regulations, | 3 | 294 |
| Edinburgh Society; the subject of their prize-essay | 4 | 299 |
| Edinburgh Med.-Chir. Transactions, | 5 | 392 |
| Edinburgh Medico-Chir. Society, transactions of | 6 | 61 |
| Edinburgh Royal Infirmary, report, | 9 | 559 |
| Edinburgh College of Surgeons, regulations of the | 11 | 552 |
| Edinburgh University, causes of its decline | 20 | 315 |
| Education, its effects upon the human frame | 3 | 190 |
| Education of girls, its evils | 3 | 372 |
| Education, importance of, to the medical man | 8 | 28 |
| Education, medical, inquiry into it | 8 | 379 |
| Edwards, Dr. on the influence of physical agents | 18 | 1 |
| Effects of opium taken in quantity | 3 | 155 |
| Effects of fear, M. Hellis on the | 8 | 462 |
| Effusion on the brain, recoveries after | 4 | 430 |
| Effusion, serous | 13 | 171 |
| Effusions into the spinal canal | 1 | 22 |
| Egg, Mr. on trusses | 15 | 516 |
| Egophony and resonance of voice | 3 | 515 |
| Egophony, description of | 4 | 462 |
| Egotism extraordinary | 12 | 280 |
| Egypt, diseases of | 19 | 194 |
| Egypt, calculous diseases in | 19 | 488 |
| Egyptian mummies, Dr. Granville on, | 5 | 46 |
| Egyptian School of Medicine | 19 | 193 |
| Elbow-joint, excision of the | 12 | 189 |
| Elbow-joint, excision of | 19 | 255 |
| Electric wire, transmissibility of diseases along | 18 | 215 |
| Electricity as a vital agent, Dr. Edwards' researches on | 4 | 288 |
| Electricity in the atmosphere, M. Pouillet on | 4 | 485 |
| Electricity, connected with cholera | 17 | 163 |
| Electricity, medical, on | 19 | 526 |
| Elementary fibre and tissues | 12 | 53 |
| Elements of physiology, Blumenbach's review of | 9 | 97 |
| Elements of surgery, Mr. Liston's | 16 | 242 |
| Elephant, fury fits of the | 14 | 254 |
| Elephantiasis, Dr. Ainslie's paper on, | 4 | 443 |
| Elephantiasis, treatment of | 4 | 448 |
| Elephantiasis Arabum, M. Biett on | 9 | 572 |
| Elephantiasis Græcorum and Arabum | 11 | 492 |
| Elephantiasis of the scrotum | 12 | 505 |
| Elephantiasis of the scrotum | 12 | 535 |
| Elephantiasis, M. Biett on | 16 | 406 |
| Elephantiasis Arabum, M. Biett on | 17 | 379 |
| Elephantiasis, Clot Bey on | 18 | 529 |
| Eleven-months' pregnancy, Dr. Collins' case | 5 | 212 |
| Elliotson, Dr. on sulphate of quinine, | 1 | 331 |
| Elliotson, Dr. his case of rupture of the stomach | 4 | 326 |
| Elliotson, Dr. on subcarbonate of iron | 6 | 345 |
| Elliotson, Dr. on sulphate of copper, | 8 | 403 |
| Elliotson, Dr. on the subcarb. of iron in tetanus | 11 | 440 |
| Elliotson, Dr. on the heart | 13 | 424 |
| Elliotson, Dr. on diseases of the heart | 14 | 60 |
| Elliotson, Dr. on glanders in man | 14 | 108 |
| Elliotson, Dr. on paralysis agitans | 14 | 167 |
| Elliotson, Dr. on diseases of the heart | 14 | 323 |
| Elliotson, Dr. on dropsy | 14 | 511 |
| Elliotson, Dr. on glanders in man | 20 | 10 |
| Elliott, Mr. his case of injury of the head | 17 | 538 |
| Elongation and displacement of colon | 12 | 570 |
| Emanations from marshes, queries as to their nature | 3 | 320 |
| Embryo, Dr. Pockels on the human, | 4 | 575 |
| Embryo in mammiferous animals | 20 | 508 |
| Embryology, Dr. Coste on | 19 | 527 |
| Emetic tartar used in 1790, by Dr. Marryat, in large doses | 11 | 253 |
| Emetics, their efficacy in hæmatemesis | 2 | 481 |
| Emetics, moxa, and blisters in insanity | 3 | 110 |
| Emetine | 1 | 50 |
| Emetine in violets | 1 | 484 |
| Emissions, seminal, on | 19 | 171 |
| Emmenagogues, Dr. Graves on | 19 | 444 |
| Emphysema from the violence of a labour | 3 | 419 |
| Emphysema post partum, a case of, | 5 | 287 |
| Emphysema of the lung, case of | 7 | 312 |
| Emphysema, Mr. Fereday on | 7 | 545 |
| Emphysema of the heart, curious case | 8 | 223 |
| Emphysema, singular case of | 10 | 533 |
| Emphysema of the lungs | 12 | 200 |
| Emphysema of the lungs, marked case of | 17 | 453 |
| Emphysema, pulmonary, in sudden death | 18 | 516 |
| Empirics, tenderness of the College to | 3 | 34 |
| Emplasto-dermic treatment of diseases | 2 | 248 |
| Empyema, Dr. Hastings' paper on | 4 | 557 |
| Empyema, successful case of operation | 4 | 562 |
| Empyema, a case of, cured by paracentesis | 5 | 267 |
| Empyema, Dr. Pitcairn's case of | 6 | 78 |
| Empyema, case of | 6 | 255 |
| Empyema, surprising case of | 7 | 491 |
| Empyema and pneumo-thorax, Dr. Duncan on | 8 | 131 |
| Empyema, M. Gasc's case of | 8 | 137 |
| Empyema, operation for, repeated | 8 | 224 |
| Empyema with external tumor | 12 | 238 |
| Empyema and pneumo-thorax | 12 | 466 |

| | Vol. | Page |
|---|---|---|
| Empyema, cases of | 13 | 447 |
| Empyema, paracentesis thoracis for, | 13 | 449 |
| Empyema, remarks on | 13 | 452 |
| Empyema, case of | 14 | 520 |
| Empyema; successful operation | 18 | 450 |
| Encephalic phlebitis, case of | 6 | 230 |
| Encephalitis, symptoms and pathology of | 6 | 409 |
| Encephaloid disease of the liver | 7 | 487 |
| Encephaloid disease of the lung | 20 | 439 |
| Encephaloid disease of the kidney | 20 | 517 |
| Encephalon, M. Raikem on disease of | 8 | 178 |
| Encephalon, memoir on the functions of the | 18 | 522 |
| Encysted dropsy cured, case of | 8 | 505 |
| Encysted tumor of the scalp | 10 | 173 |
| Encysted hydrocele of the cord | 12 | 269 |
| Encysted tumors of the liver | 20 | 11 |
| Encysted tumors of the kidney | 20 | 23 |
| Encysted tumor on the larynx | 20 | 519 |
| Endermic medication, M. Martin on, | 8 | 158 |
| Endermic medication | 8 | 203 |
| Endermic therapeutics | 18 | 176 |
| Endosmosis and exosmosis, M. Dutrochet on | 11 | 49 |
| Endosmosis and exosmosis | 18 | 213 |
| Enema, opiate, poisoning by | 13 | 563 |
| England, Dr. Clark on the climate of | 11 | 123 |
| England, southern coast of | 11 | 124 |
| England, west of | 11 | 129 |
| England, superior salubrity of | 11 | 424 |
| England, Dr. on the kidneys | 12 | 481 |
| England, cholera in | 16 | 266 |
| England, history of cholera in | 16 | 645 |
| English in Russia, invitation of the Autocrat | 9 | 166 |
| English medical politics | 12 | 454 |
| English manufacturers, health of | 16 | 77 |
| Englishmen abroad, consolation for | 9 | 270 |
| Engravings of nerves from Scarpa, &c. | 11 | 108 |
| Engravings of the bones, arteries, and nerves | 14 | 245 |
| Enlarged prostate pierced for retention | 15 | 191 |
| Enlargement of the heart, case of | 3 | 536 |
| Enlargement of the upper lip, case of, | 8 | 202 |
| Enlargement, enormous, of the uterus | 20 | 200 |
| Ennui, labour of | 19 | 185 |
| Enteritis, instructive case of | 5 | 202 |
| "Enterotome" for artificial anus, description of the | 9 | 318 |
| Entologists *v.* the Broussaians | 3 | 326 |
| Ephelides, M. Biett on | 17 | 361 |
| Epidemic of Groningen, Professor Barker on the | 8 | 189 |
| Epidemic erysipelas | 9 | 284 |
| Epidemic erysipelas, bleeding and leeching in | 9 | 494 |
| Epidemic, mysterious, at Paris | 10 | 156 |
| Epidemic croup | 11 | 169 |
| Epidemic at Gibraltar, Dr. Barry on, | 14 | 69 |
| Epidemic cholera | 16 | 163 |
| Epidemic, present, etiology of the | 16 | 484 |

| | Vol. | Page |
|---|---|---|
| Epidemic cholera, Bombay reports on | 17 | 57 |
| Epidemic in the northern circars in 1831 | 17 | 73 |
| Epidemic cholera, Dr. Webster on | 17 | 383 |
| Epidemic disease among poultry | 17 | 439 |
| Epidemic sweating fever in France | 17 | 446 |
| Epidemic cholera, Mr. Corbyn on | 18 | 63 |
| Epidemic influenza | 18 | 154 |
| Epidemics, causes of | 16 | 199 |
| Epidemics, Baron Alibert on the causes of | 18 | 477 |
| Epidemics, march of | 18 | 500 |
| Epidemics, independent of atmospheric changes | 18 | 535 |
| Epidemics, natural history of | 19 | 485 |
| Epidermis of plants | 17 | 392 |
| Epididymis, anatomy of the | 14 | 382 |
| Epilepsy, on the pathology of | 2 | 205 |
| Epilepsy, tartar-emetic ointment in | 2 | 486 |
| Epilepsy, nitrate of silver in | 3 | 160 |
| Epilepsy, musk and zinc in | 3 | 167 |
| Epilepsy, its 'local habitation' in the cerebellum | 3 | 223 |
| Epilepsy, Dr. Williams' case | 4 | 259 |
| Epilepsy in infants, history and treatment of | 5 | 163 |
| Epilepsy, tartar-emetic ointment in, | 5 | 195 |
| Epilepsy and mental alienation, their nature | 5 | 229 |
| Epilepsy, some cases of | 5 | 358 |
| Epilepsy from tænia, two cases of | 5 | 538 |
| Epilepsy, nitrate of silver in | 6 | 241 |
| Epilepsy, sympathetic, two cases of | 6 | 245 |
| Epilepsy, cases of, by Mr. Scott and Mr. Gunn | 6 | 508 |
| Epilepsy, sulphate of copper in | 8 | 552 |
| Epilepsy cured by an attack of the Parisian epidemic | 10 | 460 |
| Epilepsy cured by trephining | 12 | 504 |
| Epilepsy cured by iodine | 15 | 494 |
| Epilepsy, Dr. Bright on | 16 | 336 |
| Epilepsy combined with apoplexy | 16 | 340 |
| Epilepsy, pathology of | 17 | 178 |
| Epilepsy with scrofulous disease of cerebellum | 17 | 438 |
| Epilepsy, carotid tied for | 18 | 254 |
| Epileptic convulsions, Dr. Blake on, | 4 | 592 |
| Epileptic lunatics, treatment of | 7 | 30 |
| Epileptic, dissection of an | 8 | 136 |
| Epiploon, remarkable disease of the | 8 | 184 |
| Epistaxis, fatal, case of | 14 | 165 |
| Epizootics | 20 | 506 |
| Epps, Dr. his life of Dr. Walker | 15 | 26 |
| Epps, Dr. on counteraction | 18 | 170 |
| Erect posture natural to man | 9 | 119 |
| Erethism of the stomach | 7 | 124 |
| Erethismus ebriositatis, Dr. Blake on, | 13 | 196 |
| Ergot of rye, where procurable | 1 | 250 |
| Ergot of rye, remarks on | 3 | 418 |
| Ergot of rye, cases in which it was tried | 4 | 276 |
| Ergot of rye, effects of | 6 | 242 |
| Ergot of rye in uterine hæmorrhage, | 6 | 257 |

| | Vol. | Page |
|---|---|---|
| Ergot of rye, employment of | 7 | 278 |
| Ergot of rye, Mr. Michell on the | 9 | 358 |
| Ergot of rye in puerperal convulsions | 9 | 485 |
| Ergot of rye, cases illustrating its use | 9 | 514 |
| Ergot of rye for polypus uteri | 10 | 374 |
| Ergot of rye, observations on the | 11 | 480 |
| Ergot of rye, use of | 14 | 479 |
| Ergot of rye in hæmorrhages | 15 | 171 |
| Ergot of rye in leucorrhœa | 15 | 520 |
| Ergot in uterine hæmorrhage | 17 | 340 |
| Eruption, papular, history of | 3 | 449 |
| Eruptions, difficulties in distinguishing them | 3 | 462 |
| Erysipelas infantilis, quinine in | 1 | 224 |
| Erysipelas, ol. terebinth. in | 3 | 576 |
| Erysipelas, blood-letting in | 4 | 269 |
| Erysipelas, sulphate of quinine very valuable in | 4 | 404 |
| Erysipelas, contagious, Dr. Stephenson's cases of | 5 | 404 |
| Erysipelas œdematodes, remarks on, | 6 | 536 |
| Erysipelas, contagious | 7 | 148 |
| Erysipelas, treated by incisions | 7 | 244 |
| Erysipelas phlegmonodes, incisions in | 7 | 280 |
| Erysipelas after amputation | 7 | 554 |
| Erysipelas, death from incisions in | 7 | 536 |
| Erysipelas œdematodes, Mr. Fereday, | 7 | 556 |
| Erysipelas phlegmonodes, incisions in | 8 | 218 |
| Erysipelas treated by punctures | 8 | 514 |
| Erysipelas treated by incisions | 8 | 555 |
| Erysipelas, Sir A. Carlisle on | 8 | 569 |
| Erysipelas phlegmonodes, cases of | 9 | 179 |
| Erysipelas, Mr. Lawrence on | 9 | 361 |
| Erysipelas, inflammatory essence of | 9 | 363 |
| Erysipelas, "natural nosology" of | 9 | 366 |
| Erysipelas, causes of | 9 | 366 |
| Erysipelas, treatment of | 9 | 368 |
| Erysipelas, local applications in | 9 | 372 |
| Erysipelas, treatment by incisions | 9 | 375 |
| Erysipelas, Dr. Dobson on | 9 | 382 |
| Erysipelas, Mr. Hutchison on | 9 | 383 |
| Erysipelas, interesting case of | 9 | 574 |
| Erysipelas—effects of incisions | 10 | 236 |
| Erysipelas treated by nitrate of silver, | 10 | 445 |
| Erysipelas of the head and face | 10 | 579 |
| Erysipelas, with injury of the head | 11 | 233 |
| Erysipelas of the scalp, cases of | 14 | 180 |
| Erysipelas, followed by depôts, &c. | 14 | 237 |
| Erysipelas of the scalp, case of | 14 | 274 |
| Erysipelas, contagiousness of? | 15 | 298 |
| Erysipelas, punctures in | 15 | 300 |
| Erysipelas, extending into the skull | 15 | 302 |
| Erysipelas, erratic, M. Rennes on | 15 | 493 |
| Erysipelas, M. Biett on | 16 | 385 |
| Erysipelas treated by mercurial inunction | 18 | 175 |
| Erysipelatous inflammation | 13 | 352 |
| Erysipelatous ophthalmia | 14 | 413 |
| Erythema circinnatum, M. Biett on, | 11 | 504 |
| Eschars by lunar caustic, healing sores, &c. by | 4 | 380 |
| Esquirol, M. his treatment of insanity | 3 | 107 |
| Esquimaux, phrenological description of the | 19 | 474 |
| Esquirol on the insane | 19 | 352 |
| Essays and orations, by Sir H. Halford | 15 | 358 |
| Essays, surgical, by Mr. B. Cooper | 20 | 137 |
| Establishments, quarantine | 20 | 234 |
| Ethics, modern medical | 12 | 145 |
| Etiolation, human | 14 | 450 |
| Etiology of cutaneous diseases | 2 | 42 |
| Etiology of scrofula | 2 | 98 |
| Etiology of hæmorrhoids | 2 | 285 |
| Etiology of goitre | 2 | 442 |
| Etiology of the Penitentiary endemic, | 3 | 28 |
| Etiology of epidemics, Dr. Smith's | 3 | 62 |
| Etiology of the "Dock-yard disease" | 4 | 85 |
| Etiology of the epidemic Indian cholera | 4 | 94 |
| Euphorbia lathyrus, a substitute for croton | 3 | 273 |
| Euphorbia lathyrus | 3 | 589 |
| Euphorbia lathyrus, oil of, just imported | 4 | 301 |
| Eustachian tube, injection of | 1 | 43 |
| Eustachian tube, chronic inflammation of | 14 | 469 |
| Eustachian tube, mode of passing a bougie | 17 | 432 |
| Evacuations suppressed with danger, | 13 | 467 |
| Evans, Mr. on neuralgia | 1 | 198 |
| Evolution of the chick, Carus on the, | 18 | 171 |
| Examen des doctrines medicales, notice of it | 5 | 1 |
| Examination of recruits | 9 | 92 |
| Examinations in medicine | 10 | 241 |
| Exanthematous ophthalmia, Mr. Wardrop on | 5 | 393 |
| Excerpta choleralogica | 17 | 550 |
| Excerpta choleralogica | 18 | 261 |
| Excision of piles, arguments *pro* et *con* | 4 | 362 |
| Excision of nævi | 11 | 172 |
| Excision of nerves in tic douloureux, | 12 | 471 |
| Excision of joints, Mr. Syme on the, | 13 | 273 |
| Excision of a carious rib, fatal | 13 | 554 |
| Excision of diseased joints, Mr. Syme | 15 | 113 |
| Excision of joints, method of performing | 15 | 116 |
| Excision of shoulder-joint | 15 | 117 |
| Excision of elbow-joint | 15 | 119 |
| Excision of wrist-joint | 15 | 120 |
| Excision of hip and knee-joints | 15 | 121 |
| Excision of ankle-joint | 15 | 123 |
| Excision of scirrhous rectum | 15 | 159 |
| Excision of the cervix uteri, *successful* | 15 | 278 |
| Excision of head of humerus, cases of | 18 | 274 |
| Excoriation-sore, Mr. Johnson on the | 20 | 539 |
| Excrescences, curious case of | 9 | 189 |
| Excurvation of the spine | 2 | 114 |
| Exercise in distortion | 3 | 380 |
| Exercise, Dr. Johnson on | 10 | 520 |
| Exercise, corporeal, benefits of | 12 | 303 |

| | Vol. | Page |
|---|---|---|
| Exhalation of blood from the stomach | 8 | 216 |
| Exhalation, aromatic, from the arm, | 19 | 215 |
| Exhaustion in parturition, Dr. Davis on | 3 | 410 |
| Exhaustion, with sinking | 13 | 64 |
| Exhaustion, death from, after lithotomy | 15 | 263 |
| Exhumation and fumigation | 6 | 268 |
| Exomphalos, strangulated, case of | 8 | 479 |
| Exophthalmos | 9 | 565 |
| Exostoses, large, arising in infancy | 18 | 167 |
| Exostosis from the dorsum ilii | 9 | 565 |
| Exostosis, wonderful conversion of, into adipocire | 10 | 157 |
| Exostosis cured by mercury | 13 | 456 |
| Expectoration in phthisis, acid and alkaline | 2 | 452 |
| Expectoration in pneumonia | 3 | 516 |
| Expectoration, various kinds of | 4 | 465 |
| Expectoration of pus, mysterious | 13 | 567 |
| Experiments, strictures on | 1 | 182 |
| Experiments with calomel in large doses | 4 | 381 |
| Experiments on post-mortem changes | 6 | 474 |
| Exposition of state of Coll. of Surgeons | 19 | 565 |
| Exposure to air in phthisis | 12 | 241 |
| Exstrophy of the urinary bladder | 19 | 505 |
| External iliac, ligature of | 7 | 177 |
| External iliac, ligature of the | 8 | 557 |
| External iliac, ligature of the | 9 | 261 |
| External iliac artery tied, with remarks | 11 | 513 |
| Extinction of fire, recipe for | 2 | 530 |
| Extirpation of the parotid gland | 1 | 216 |
| Extirpation of the ovaria | 2 | 215 |
| Extirpation of the uterus, Dr. Siebold's case of | 3 | 264 |
| Extirpation of the uterus, Dr. Holscher's case of | 3 | 266 |
| Extirpation of the tonsils, Mr. Pettigrew's | 3 | 545 |
| Extirpation, successful, of a scirrhous parotid | 5 | 223 |
| Extirpation of uterus, Dr. Hesse on, | 8 | 195 |
| Extirpation of the kidneys, results of, | 8 | 208 |
| Extirpation of the cervix uteri, most wonderful case of | 9 | 518 |
| Extirpation of the womb, by Dr. Blundell | 9 | 519 |
| Extirpation of the breast, fatal results of | 10 | 238 |
| Extirpation of the tonsil | 11 | 208 |
| Extirpation, successful, of the uterus, | 11 | 564 |
| Extirpation of the ovaria, cases of | 12 | 440 |
| Extra-limites | 2 | 536 |
| Extra-uterine conception, Dr. Wishart's case | 4 | 280 |
| Extra-uterine and super-fœtation | 4 | 285 |
| Extra-uterine fœtus successfully removed | 5 | 275 |
| Extra-uterine pregnancy, new kind of | 5 | 518 |
| Extra-uterine conception, Dr. Doudement on | 5 | 618 |
| Extra-uterine conception, case of | 5 | 529 |
| Extra-uterine fœtus, Mr. Mackie's case | 6 | 496 |
| Extra-uterine pregnancy, case of | 8 | 231 |
| Extra and intra-uterine fœtation | 9 | 267 |
| Extra-uterine fœtation, remarkable case of | 10 | 223 |
| Extra-uterine fœtation, case of | 15 | 187 |
| Extra-uterine pregnancy, cases of | 17 | 310 |
| Extra-uterine pregnancy, case of | 19 | 506 |
| Extra-uterine peritoneal pregnancy | 20 | 239 |
| Extract of lettuce, a substitute for opium | 2 | 531 |
| Extract of garden lettuce, Dr. Francois on | 5 | 522 |
| Extraction of the ovaria considered | 3 | 336 |
| Extraction of diseased teeth and roots | 6 | 214 |
| Extraction of a foreign body in larynx | 13 | 465 |
| Extraneous bodies in the ear, extraction of | 3 | 113 |
| Extravasations in the chest | 2 | 71 |
| Extravasation of urine, incisions for, | 10 | 222 |
| Extravasation of urine, fatal case of | 12 | 275 |
| Eye, its indications in disease | 2 | 425 |
| Eye, peculiarly destructive inflammation of the | 6 | 342 |
| Eye, case of deep-seated inflammation in the | 7 | 265 |
| Eye, inflammation of, after typhus fever | 9 | 223 |
| Eye, unsuccessful removal of | 9 | 565 |
| Eye, affections of the | 11 | 506 |
| Eye, destruction of, in parturition | 12 | 35 |
| Eye, fungus hæmatodes of the | 12 | 236 |
| Eye, Mr. Lawrence on the venereal diseases of the | 14 | 80 |
| Eye, Mr. Lawrence on venereal diseases of the | 14 | 116 |
| Eye, Mr. Mackenzie on the diseases of the | 14 | 397 |
| Eye, injuries of the | 15 | 20 |
| Eye, Mr. Mackenzie on its diseases | 15 | 52 |
| Eye, on the strumous affections of | 19 | 258 |
| Eye, the effects of cholera upon the, | 19 | 478 |
| Eye, diseases of, Mr. Lawrence on | 20 | 40 |
| Eyeball, medullary tumor of the | 11 | 397 |
| Eyeball, suppuration of the | 19 | 444 |
| Eyelids and orbit, cancer of the | 11 | 397 |
| Eyelids, syphilitic ulceration of the | 14 | 135 |
| Eyes for the blind, Mr. Adams' | 13 | 481 |
| Eyes, malformations of the | 20 | 370 |
| Eyes, errors in number | 20 | 373 |
| Eyes, errors in position | 20 | 374 |
| Eyes, errors in size | 20 | 375 |

### F

| | Vol. | Page |
|---|---|---|
| Face-presentation considered | 4 | 154 |
| Face and forehead, form of | 10 | 506 |
| Face, malignant diseases of the | 11 | 395 |
| Face, medullary tumor of the | 11 | 396 |
| Face, distortion of the, oddly cured | 12 | 264 |
| Face, ulcerations of the | 19 | 279 |

| | Vol. | Page |
|---|---|---|
| Facial neuralgia, case of | 15 | 511 |
| Facial hemiplegia; use of phosph. ext. | 20 | 204 |
| Factories Bill, on the | 19 | 181 |
| Factory commission, report of the | 20 | 390 |
| Factory commission, organization of, | 20 | 391 |
| Factory commission, its instructions, | 20 | 392 |
| Factory commission, its mode of inquiry | 20 | 393 |
| Factory report, Sir David Barry's | 20 | 393 |
| Factory report, Dr. Loudon's | 20 | 398 |
| Factory report, Dr. Hawkins' | 20 | 400 |
| Factory report, Dr. Woolriche's | 20 | 401 |
| Facts, study of | 14 | 308 |
| Facts, generalization of | 14 | 310 |
| Facts, Dr. Todd's method of classifying | 16 | 40 |
| Fæces, accumulation of, in large intestines | 9 | 211 |
| Failures in lithotomy, Mr. Fletcher | 15 | 365 |
| Fallopian tube, rupture of | 1 | 234 |
| Falrich, Dr. his wax models | 17 | 242 |
| False reports! | 10 | 182 |
| False aneurism of the heart | 11 | 249 |
| False conceptions, Dr. Montgomery on | 20 | 124 |
| Falx, fungous tumor of the | 13 | 496 |
| Fantonelli on the itch | 19 | 215 |
| Farre, Dr. on angina pectoris | 10 | 196 |
| Fascia transversalis, Cloquet on | 19 | 471 |
| Fasciæ of the pelvis | 10 | 534 |
| Fasciculus of engravings from Chatham | 3 | 57 |
| Fasting, extraordinary cases of | 19 | 204 |
| Fat, *not* formed in the great intestines | 4 | 31 |
| Fat and blood vomited, a remarkable case | 5 | 247 |
| Fatal case of fever | 3 | 24 |
| Fatigue, a plea against exercise | 3 | 193 |
| Fatty matters in the stomach taken for arsenic | 3 | 290 |
| Fatty discharges from the bowels | 19 | 477 |
| Fatty discharge from the bowels | 20 | 8 |
| Faulkner, Sir A. B. his rambling notes | 8 | 27 |
| Fawdington, Mr. his case of melanosis | 5 | 335 |
| Fawdington, Mr. on nævus | 13 | 506 |
| Fear, its influence on the human frame | 4 | 110 |
| Fear, effects of, on operations | 9 | 236 |
| Fear of contagion | 18 | 148 |
| Febrile miasm, its mode of action on the system | 8 | 59 |
| Fecula, adulterations of | 20 | 516 |
| Fecundation of plants | 17 | 393 |
| Fecundity, causes of | 20 | 160 |
| Fecundity greater in the country | 20 | 162 |
| Fees, medical, in Turkey | 19 | 436 |
| Fellows of the College do not *consult* with surgeons | 8 | 33 |
| Fels, Dr. on softening of the stomach | 11 | 530 |
| Female youth, diseases of | 7 | 317 |
| Female diseases, Dr. Marshall Hall on | 11 | 289 |
| Female diseases, Dr. Gooch on | 11 | 289 |
| Female, lithotrity unsuccessful in the | 12 | 209 |
| Female breast, tumors of the | 12 | 524 |

| | Vol. | Page |
|---|---|---|
| Female disorders, Dr. Addison on | 13 | 39 |
| Female jurors, ignorance of | 20 | 122 |
| Females, venereal disease in | 18 | 388 |
| Females, examination of | 19 | 446 |
| Femoral hernia, curious case of | 6 | 611 |
| Femoral aneurism—iliac artery tied, | 8 | 557 |
| Femoral artery, wound of the | 9 | 257 |
| Femoral hernia, remarkable case of | 10 | 574 |
| Femoral artery and vein, wound of the, case of | 10 | 589 |
| Femoral hernia, diagnosis of | 12 | 453 |
| Femoral artery, ligature of the | 13 | 276 |
| Femoral artery tied, cases of | 13 | 283 |
| Femur; supposed case of partial fracture of it | 5 | 249 |
| Femur, fracture of neck of the | 17 | 165 |
| Fenning, E. her trial | 3 | 179 |
| Fereday, Mr. prize hospital report | 7 | 537 |
| Fergusson, Dr. on cholera | 16 | 580 |
| Fergusson, Dr. on contagion | 17 | 465 |
| Fetid abscesses near mucous membranes | 17 | 433 |
| Fever, new theory of | 1 | 439 |
| Fever, eclectic article on | 2 | 1 |
| Fever, Dr. Hosack on | 2 | 54 |
| Fever, caused by panic | 2 | 148 |
| Fever, intermittent, partial | 2 | 201 |
| Fever from animal putrefaction | 2 | 202 |
| Fever, Laennec's report on | 2 | 240 |
| Fever, congestive, on the sea | 2 | 249 |
| Fever, remarks on, by Dr. Dawson, | 2 | 394 |
| Fever, idiopathic, cases of | 3 | 525 |
| Fever, *symptomatic*, treatment of | 4 | 377 |
| Fever, a bugbear to French physicians | 4 | 570 |
| Fever, an inquiry into its seat and nature | 5 | 1 |
| Fever, idiopathic, its onset & progress | 5 | 9 |
| Fever, Dr. Chambers' cases of | 6 | 161 |
| Fever, Dr. Hewett on | 6 | 198 |
| Fever, M. Broussais' views of | 6 | 336 |
| Fever, Dr. Bally on the seat of | 7 | 498 |
| Fever, *latent* period of | 8 | 61 |
| Fever, Dr. Marsh on | 8 | 55 |
| Fever, pathology of | 8 | 495 |
| Fever, discussions on | 8 | 567 |
| Fever, eclectic review on | 9 | 1 |
| Fever, clinical report on | 9 | 245 |
| Fever, Dr. O'Reardon on | 10 | 259 |
| Fever, treatment of | 10 | 326 |
| Fever, Dr. O'Reardon on | 11 | 181 |
| Fever at Arracan, characters of the | 11 | 320 |
| Fever, &c. Dr. Stoker's observations, | 11 | 337 |
| Fever, treatment of | 11 | 349 |
| Fever, pathological appearances in | 11 | 353 |
| Fever, Dr. Smith on | 12 | 337 |
| Fever, Dr. Tweedie on | 12 | 337 |
| Fever, doctrines of | 12 | 338 |
| Fever, essential symptoms of | 12 | 340 |
| Fever, Dr. Johnson's views of | 12 | 342 |
| Fever and inflammation not the same | 12 | 344 |
| Fever, synochus, description of | 12 | 346 |
| Fever, functional disturbance in | 12 | 347 |

| | Vol. | Page |
|---|---|---|
| Fever, Drs. Smith and Tweedie on .. | 12 | 385 |
| Fever, causes of. | 12 | 385 |
| Fever, treatment of | 12 | 386 |
| Fever, causes of. | 12 | 396 |
| Fever, sources of, at Gibraltar | 13 | 52 |
| Fever, Dr. Smith's letter on | 13 | 230 |
| Fever, Dr. Hall on loss of blood in.. | 13 | 324 |
| Fever at Gibraltar, contagiousness of, considered | 13 | 343 |
| Fever and cholera, resemblances | 13 | 379 |
| Fever hospital, Dr. O'Brien's report, | 13 | 408 |
| Fever, typhoid, types of | 13 | 409 |
| Fever, state of the brain in | 13 | 411 |
| Fever at Gibraltar, Dr. Gilkrest on.. | 14 | 224 |
| Fever, state of intestinal canal in | 14 | 443 |
| Fever, rapidly fatal case of | 14 | 472 |
| Fever, climatorial and endemic, of Africa | 16 | 411 |
| Fever, Dr. Stevens's crotchets on .. | 17 | 323 |
| Fever, report on, by Dr. Hackett .. | 18 | 149 |
| Fever, after operations | 18 | 404 |
| Fever, M. Andral on | 19 | 236 |
| Fever, petechial, in Italy | 19 | 508 |
| Fever, Dr. Roots on | 19 | 538 |
| Fever, Dr. Latham on | 19 | 544 |
| Fevers, intermittent and remittent.. | 3 | 75 |
| Fevers, local bleeding in | 7 | 49 |
| Fibrinous polypi of the heart | 20 | 551 |
| Fingers and toes, Dr. Rynd on the .. | 14 | 214 |
| Fingers, contraction of the | 17 | 175 |
| Fingers, fatal case of amputation of, | 17 | 530 |
| Fingers, state of, in phthisis | 18 | 189 |
| Fingers, on permanent retraction of, | 18 | 290 |
| Fingers, permanent flexions of the .. | 18 | 461 |
| Fish not so digestive as is imagined.. | 6 | 16 |
| Fishes, as affected by physical agents | 18 | 8 |
| Fishes, life of, out of water | 18 | 9 |
| Fishes, epidemics among | 19 | 485 |
| Fissure of anus, Dupuytren on | 19 | 542 |
| Fissures, hæmorrhoidal | 2 | 281 |
| Fistula lacrymalis, success of iodine in | 4 | 11 |
| Fistula in perineo cured, Mr. White's case | 6 | 245 |
| Fistula of the parotid duct, M. Minault on | 9 | 473 |
| Fistula in perineo, new mode of treating | 11 | 265 |
| Fistula of the parotid duct, treatment of | 11 | 443 |
| Fistula ani—a *fistulous* report | 14 | 157 |
| Fistula perinei, report on | 14 | 501 |
| Fistula ani, Mr. Liston's treatment of, | 18 | 330 |
| Fistula ani, Graefe's treatment of .. | 20 | 219 |
| Fistula, congenital, in the neck, memoir on | 20 | 481 |
| Fistulæ, M. Roux's mode of treating, | 5 | 594 |
| Fistulæ treated by iodine | 10 | 131 |
| Fistulous sinuses, M. Dupuytren on, | 18 | 303 |
| Flatfootedness, treatment of | 20 | 520 |
| Flaubert, M. on the reduction of dislocations | 7 | 452 |
| Fleming, Mr. his ligature of the carotid | 6 | 167 |
| Fleming, Mr. on disease of the nose, | 19 | 454 |
| Fletcher, Mr. his notes & illustrations | 15 | 364 |
| Fletcher, Mr. on prolapsus ani | 15 | 483 |
| Fletcher, Mr. on the influence of the mind on the body | 19 | 341 |
| Fletcher, Dr. his case of apoplectiform disease | 20 | 164 |
| Fleurens on the brain | 1 | 179 |
| Flexure, abnormal, of the colon | 20 | 200 |
| Flooding labour, Mr. Ashwell on | 10 | 112 |
| Flora medica | 8 | 120 |
| Florence, a medical glance at | 6 | 96 |
| Florence, climate of | 14 | 455 |
| Flour, the panacea for burns | 12 | 199 |
| Fluidity of the blood after death | 11 | 185 |
| Fluids of the mother, their effects on the fœtus | 1 | 356 |
| Fluids, state of, in typhus fever | 9 | 348 |
| Fodéré, M. his case of abortion | 2 | 529 |
| Fodéré, M. on the conium mac. | 19 | 218 |
| Fœtal monstrosity, curious case of.. | 17 | 162 |
| Fœtal heart, sounds of | 20 | 105 |
| Fœticide, prevalence of | 1 | 340 |
| Fœticide, on the crime of | 1 | 340 |
| Fœtus in the abdomen of a boy | 1 | 180 |
| Fœtus in embryo, diseases of the | 8 | 507 |
| Fœtus, fungus hæmatodes in the | 12 | 163 |
| Fœtus in utero, vaccination of the .. | 12 | 214 |
| Fœtus, modes of proving it to have lived | 13 | 104 |
| Fœtus, sanguification of the | 15 | 48 |
| Fœtus, organic diseases of | 19 | 140 |
| Fœtus, position of the | 19 | 235 |
| Fœtus, position of | 19 | 515 |
| Fœtus, discharge of a, from umbilicus | 20 | 162 |
| Fœtus breathing in utero | 20 | 163 |
| Fœtus, turning of, by the head | 20 | 237 |
| Follicular ulceration, Dr. Hewett on, | 5 | 557 |
| Follicular ulceration, case of | 7 | 490 |
| Fomentations, some choice ones | 3 | 351 |
| Food, new article of | 19 | 506 |
| Foramen ovale, open, in adults | 9 | 249 |
| Foramen ovale, open, cases of | 11 | 230 |
| Foramen ovale, open, case of | 19 | 212 |
| Foramen ovale, open, case of | 19 | 242 |
| Forbes, Dr. J. on the stethoscope, &c. | 2 | 61 |
| Forbes, Dr. his 2d edition of Laennec | 8 | 103 |
| Forceps for extracting teeth perpendicularly | 2 | 560 |
| Forceps, average estimate of its use.. | 3 | 398 |
| Forceps, its employment considered, | 3 | 403 |
| Forceps, its history | 3 | 407 |
| Forceps, when to be used | 3 | 409 |
| Forceps, its dangers and evils pointed out | 3 | 428 |
| Forceps, long, remarks on their use, | 4 | 149 |
| Forceps, long, Dr. Davis's modification of the | 4 | 153 |
| Forceps' operations, treatment after them | 4 | 156 |
| Forcible stretching of the spine, Mr. Shaw on | 3 | 375 |

| | Vol. | Page |
|---|---|---|
| Foreign bodies in bodies natural.... | 8 | 483 |
| Foreign bodies in the larynx, cases of | 10 | 517 |
| Foreign opinions on medical politics, | 12 | 454 |
| Foreign body in trachea .......... | 13 | 464 |
| Foreign body in larynx extracted... | 13 | 465 |
| Foreign medical politics .......... | 20 | 561 |
| Formation of bone; Monro's opinion | 4 | 56 |
| Formula of a powerful tonic ....... | 4 | 374 |
| Formulary of the French hospital medicines .................. | 3 | 350 |
| Formulary of the Parisian hospitals.. | 12 | 540 |
| Fothergillian medal, Mr. Bampfield, | 1 | 251 |
| Fouquier, M. his views ......... .. | 3 | 99 |
| Fracture of the patella and neck of the femur .................. | 4 | 221 |
| Fracture of the skull, with depression, Dr. Corbyn's case ...... | 4 | 241 |
| Fracture of the spine, M. Lisfranc on, | 5 | 560 |
| Fracture of the spine, case of ...... | 6 | 605 |
| Fracture, ununited, of the tibia .... | 7 | 315 |
| Fracture of the spine—extension employed .................. | 8 | 201 |
| Fracture of a cervical vertebra...... | 8 | 478 |
| Fracture of the trochanters—tetanus, | 9 | 176 |
| Fracture of the spine, case of ...... | 9 | 477 |
| Fracture of the tibia—traumatic delirium.................... | 9 | 479 |
| Fracture of the upper extremity of the humerus ................ | 10 | 135 |
| Fracture of the neck of the humerus mistaken................ | 10 | 140 |
| Fracture of bones in cancer........ | 11 | 449 |
| Fracture, cases of................ | 11 | 508 |
| Fracture, compound, of the skull, cases of .................... | 17 | 512 |
| Fracture, compound of the femur .. | 18 | 162 |
| Fracture of a rib, from coughing.... | 19 | 441 |
| Fracture of the lower jaw, Dr. Chester on .. ............... | 20 | 469 |
| Fracture-bed, plate of Mr. Earle's .. | 1 | 63 |
| Fractured spine, symptoms of...... | 3 | 43 |
| Fractured ribs—empyema ........ | 8 | 465 |
| Fractured bones, non-union of .... | 8 | 524 |
| Fractured cervix humeri .......... | 9 | 235 |
| Fractured humerus, non-union of .. | 10 | 568 |
| Fractured thigh, fatal case of ...... | 14 | 228 |
| Fractured leg, non-union of........ | 14 | 229 |
| Fractured bones, re-union of ...... | 15 | 193 |
| Fractured pelvis ................ | 17 | 535 |
| Fractured sternum .............. | 17 | 536 |
| Fractured scapula................ | 17 | 537 |
| Fractured cervix femoris, bony union | 20 | 170 |
| Fractured limbs treated by plaster-moulds .................... | 20 | 492 |
| Fractures, Mr. H. Earle on ........ | 1 | 58 |
| Fractures, on non-union of ........ | 2 | 249 |
| Fractures of the patella, Amesbury's apparatus ........... | 3 | 301 |
| Fractures and contusions, severe, cases of .................... | 5 | 115 |
| Fractures of the spine not *necessarily* fatal.................... | 6 | 107 |
| Fractures, Baron Larrey on......... | 8 | 202 |
| Fractures and dislocations of upper extremity .................. | 10 | 132 |
| Fractures, treatment of, at Glasgow, | 10 | 165 |
| Fractures and dislocations, cases of.. | 10 | 175 |
| Fractures of the neck of thigh-bone, | 10 | 235 |
| Fractures, simple and compound, remarks on.................... | 11 | 226 |
| Fractures, successful issue of ...... | 12 | 172 |
| Fractures, simple and compound.... | 12 | 566 |
| Fractures, treated by plaster casts .. | 16 | 247 |
| Fractures, partial, in children...... | 16 | 588 |
| Fractures of the spine ...... ..... | 17 | 14 |
| Fractures of fibula, M. Dupuytren on | 18 | 308 |
| Fractures of patella, M. Dupuytren on | 18 | 316 |
| Fractures of the extremities, M. Dupuytren on ............... | 18 | 317 |
| Fractures of bones by muscles..... | 18 | 531 |
| Fractures of neck of the thigh-bone, | 19 | 83 |
| Fragilitas ossium ................ | 10 | 413 |
| France and Germany, their surgery compared .................... | 4 | 213 |
| French professors, scandalous occurrence amongst............ | 4 | 283 |
| France, climate of................ | 11 | 130 |
| France, south-east of ............ | 11 | 132 |
| France, characteristics of.......... | 14 | 450 |
| Francis, Dr. his case of laryngitis ... | 3 | 209 |
| Frank's description of cholera....... | 16 | 631 |
| Franklin, Sir William—his death ... | 20 | 572 |
| Fraser, Mr. his critique on Dr. Barry | 13 | 337 |
| Freemason's Tavern, the illustrious radicals .................... | 10 | 305 |
| French 'amour propre' instanced ... | 3 | 349 |
| French *pratique*, Dr. Scudamore's ideas on.................... | 5 | 74 |
| French criticism, or vive la *grande nation!* .................... | 5 | 253 |
| French medical reform............. | 10 | 542 |
| French machines for the spine...... | 13 | 58 |
| French operations................ | 14 | 564 |
| Fright, idiotcy from .............. | 13 | 512 |
| Frogs, in water, existence of ...... | 18 | 4 |
| Frontal sinus, Dr. Milligan on the .. | 19 | 359 |
| Froriep, Dr. manual of midwifery .. | 19 | 418 |
| Froriep on induction of labour...... | 19 | 420 |
| Froriep, Dr. on the cholera........ | 19 | 514 |
| Frost, Mr. on the gathering of the colchicum flowers............ | 3 | 573 |
| Fruits in dyspepsia .............. | 6 | 17 |
| Fumigation, mercurial, Dr. Gibson on, | 3 | 568 |
| Functional disorders, Dr. Philip on.. | 13 | 311 |
| Functions of the brain............ | 1 | 179 |
| Fungating venereal sore, Dr. Hart on, | 20 | 165 |
| Fungoid tumor of the uterus, case of, | 3 | 287 |
| Fungoid disease of the breast, case of, | 8 | 128 |
| Fungoid tumors of the diaphragm and lung ................... | 8 | 481 |
| Fungoid disease of the ovaria ...... | 12 | 422 |
| Fungoid disease of the testis........ | 14 | 1 |
| Fungoid disease of the testis........ | 14 | 263 |
| Fungoid disease, extensive, case of.. | 15 | 304 |

| | Vol. | Page |
|---|---|---|
| Fungoid tumor of neck—operation fatal | 16 | 525 |
| Fungoid tumor of the dura mater | 20 | 526 |
| Fungous eruption, curable by mercury | 8 | 401 |
| Fungous tumors from the gum—operation | 10 | 588 |
| Fungous tumors of meninges | 13 | 494 |
| Fungous tumor of radius, case of | 15 | 522 |
| Fungus hæmatodes, case of | 3 | 227 |
| Fungus melanodes, Mr. Wardrop's account of | 4 | 122 |
| Fungus of the uterus extirpated by ligature | 4 | 544 |
| Fungus hæmatodes of the left lung | 6 | 519 |
| Fungus on the dura mater, hemiplegia, | 8 | 558 |
| Fungus hæmatodes—carodid artery tied | 9 | 256 |
| Fungus hæmatodes of the stomach, cases of | 9 | 400 |
| Fungus hæmatodes of the breast—amputation | 10 | 255 |
| Fungus hæmatodes of the brain | 12 | 217 |
| Fungus hæmatodes, cases of | 12 | 235 |
| Fungus of the dura mater | 12 | 489 |
| Fungus hæmatodes of stomach & liver | 12 | 563 |
| Fungus hæmatodes of the lung | 13 | 189 |
| Fungus hæmatodes of the liver | 13 | 226 |
| Fungus hæmatodes of the lungs | 13 | 514 |
| Fungus hæmatodes said to be cured, | 15 | 155 |
| Fungus cerebri, cases of | 16 | 518 |
| Fungus of dura mater and cranium | 17 | 483 |
| Funis, omission of ligature to | 1 | 350 |
| **G** | | |
| Gairdner, Dr. on iodine | 1 | 104 |
| Gairdner, Dr. on perforations of the stomach | 2 | 170 |
| Gairdner, Dr. on purpura hæmorrhagica | 2 | 193 |
| Gairdner, Dr. his case of carditis | 6 | 79 |
| Gairdner, Dr. his case of disorganized stomach | 6 | 86 |
| Gairdner, Dr. on mineral springs | 19 | 28 |
| Gaitskell, Mr. his obstetrical bandage | 9 | 177 |
| Gaitskill, Mr. on spinal consumption, | 1 | 456 |
| Galen, historic notice of | 15 | 400 |
| Gall, Dr. death of | 10 | 190 |
| Gall-bladder, case of inflammation of | 7 | 187 |
| Gallic *practice* in cephalitis | 3 | 216 |
| Galvanism for dissolving calculi | 1 | 223 |
| Galvanism and nervous influence | 15 | 83 |
| Galvanism, medical | 19 | 503 |
| Galvanism, misapplication of, in physiological experiments | 20 | 226 |
| Gambling, effects of | 8 | 438 |
| Ganglia, semilunar, inflamed | 1 | 187 |
| Ganglia of the sympathetic, state in tetanus | 3 | 388 |
| Ganglion, Dr. Cumin's mode of treating | 3 | 551 |
| Ganglionic and cerebral system of nerves considered | 6 | 316 |

| | Vol. | Page |
|---|---|---|
| Ganglionic system, researches on the, | 14 | 33 |
| Ganglionic system, general & special functions of | 14 | 34 |
| Ganglionic system, its influence on various organs | 14 | 35 |
| Gangræna senilis, Dr. Andry on | 10 | 151 |
| Gangræna senilis, Dupuytren on | 19 | 492 |
| Gangrene of the heart | 1 | 189 |
| Gangrene at the mouth of the uterus in puerperal fever | 2 | 511 |
| Gangrene after extraction of an ovarium | 3 | 342 |
| Gangrene of the foot, Mr. Chevalier's case | 4 | 324 |
| Gangrene, treatment of | 4 | 402 |
| Gangrene of the lungs, M. Bouillaud's paper on | 4 | 498 |
| Gangrene from cold | 5 | 84 |
| Gangrene of the feet, case of | 8 | 187 |
| Gangrene of the lungs, case of | 8 | 226 |
| Gangrene of the lungs, not fatal | 9 | 168 |
| Gangrene after fracture—cured by depletion | 9 | 237 |
| Gangrene, traumatic, cases of, and remarks on | 10 | 145 |
| Gangrene of a limb after wound of its principal vessel | 10 | 593 |
| Gangrene, deceptive appearances of | 11 | 228 |
| Gangrene after operating for popliteal aneurism | 11 | 263 |
| Gangrene of the teeth, or caries | 11 | 330 |
| Gangrene, hospital, Dr. Adam on | 11 | 371 |
| Gangrene of the left upper extremity, | 13 | 180 |
| Gangrene of the leg from scalp-wound | 14 | 235 |
| Gangrene, spontaneous, case of | 18 | 182 |
| Gangrene, spontaneous, from arteritis | 19 | 207 |
| Gangrene of the lungs, Bouillaud on, | 19 | 209 |
| Gangrene, spreading, amputation in, | 20 | 265 |
| Gangrenous erysipelas, odd case | 8 | 517 |
| Gangrenous ulcer, Mr. Leslie on | 11 | 374 |
| Gangrenous ulcer, treatment of | 11 | 377 |
| Gangrenous erysipelas | 14 | 499 |
| Gardner peerage case, report on | 5 | 170 |
| Gardner, Mr. on purpura | 19 | 153 |
| Gargle, a smart mercurial one. | 3 | 352 |
| Gastralgia mistaken for gastritis, Dr. Barras on | 5 | 489 |
| Gastralgia, some cases of | 5 | 493 |
| Gastralgia and gastritis, diagnosis of, | 5 | 496 |
| Gastralgia, Dr. Barras on | 8 | 45 |
| Gastralgia, severe case of | 8 | 48 |
| Gastralgia, chlorotic, on the | 19 | 220 |
| Gastric *excitation* | 7 | 80 |
| Gastric & diaphragmatic perforation, | 10 | 221 |
| Gastric irritation | 12 | 251 |
| Gastritis, a peculiar species of | 2 | 173 |
| Gastritis, Dr. Uwirs's view of | 2 | 391 |
| Gastritis, its prevalence in phthisical patients | 3 | 530 |
| Gastritis, chronic, case of | 6 | 261 |
| Gastritis, sero & muco, symptoms of, | 9 | 355 |
| Gastritis, acute, with arachnitis | 13 | 158 |

| | Vol. | Page |
|---|---|---|
| Gastritis, Dr. Stokes on | 19 | 175 |
| Gastrodynia, its history & treatment, | 2 | 396 |
| Gastrodynia, M. Barrut on | 7 | 408 |
| Gastrodynia, Dr. Abercrombie on | 10 | 340 |
| Gastro-enteralgia, M. Legallois on | 9 | 271 |
| Gastro-enterite, fatal case of | 7 | 481 |
| Gastro-enterite, considerations on | 9 | 10 |
| Gastro-enterite simulating cholera | 18 | 173 |
| Gastro-enteritis, supposed case of | 2 | 463 |
| Gastro-enteritis, how it causes death, | 3 | 330 |
| Gastro-enteritis, progress of, &c | 5 | 25 |
| Gastro-enteritis, M. Broussais' view of | 6 | 327 |
| Gastro-enteritis, treatment of | 7 | 57 |
| Gastro-enteritis, without pain | 7 | 458 |
| Gastro-enteritis, acute, cases of | 8 | 145 |
| Gastro-enteritis of infants | 9 | 471 |
| Gastrology—diet and longevity | 9 | 217 |
| Gastro-malaxia, Dr. Fels on | 11 | 530 |
| Gastrotomy in ruptured uterus, case, | 4 | 26 |
| Gastrotomy, a successful instance of, | 4 | 285 |
| Gastrotomy, a successful case of, at Parma | 6 | 100 |
| Gastrotomy for strangulated intestine | 7 | 223 |
| Gastrotomy, successful case of | 19 | 491 |
| Gateshead, Dr. White on cholera at | 16 | 541 |
| Gattei's new lithotome | 20 | 203 |
| General practitioner, observations on the | 2 | 246 |
| General practitioners vilified by the Lancet | 8 | 550 |
| General anatomy, Mr. Grainger's | 12 | 49 |
| General poisoning, remarks on | 12 | 365 |
| General practitioners | 12 | 486 |
| General practitioners, dinner of the | 12 | 518 |
| General practitioners, dinner of the | 13 | 192 |
| General practitioners, society of the | 13 | 270 |
| Geneva, characteristics of | 14 | 452 |
| Genitals in women, inflammation of, | 15 | 414 |
| Genitals, ulcers of the | 15 | 416 |
| Genito-urinary organs, neuralgia of | 11 | 276 |
| Genius, infirmities of, by Mr. Madden | 19 | 316 |
| Genoa to Nice, new road | 14 | 462 |
| Gentianin | 1 | 54 |
| George the Fourth, illness & death of | 13 | 468 |
| George the Fourth, post-mortem examination of | 13 | 470 |
| George, Mr. on the small-pox | 20 | 59 |
| Georget, M. on insanity | 6 | 482 |
| German literature, extracts from | 19 | 493 |
| German whey-establishments | 20 | 197 |
| Ghosts at Wakefield, with speculations thereon | 4 | 490 |
| Gibbs, Dr. his removal of a nervous tumor | 12 | 184 |
| Gibraltar epidemic | 10 | 564 |
| Gibraltar fever, Dr. Barry on the | 12 | 539 |
| Gibraltar, medical topography of | 13 | 49 |
| Gibraltar fever, critique of Dr. Barry on | 13 | 337 |
| Gibraltar fever, treatment of | 13 | 348 |
| Gibraltar epidemic, Dr. Barry on the, | 14 | 69 |

| | Vol. | Page |
|---|---|---|
| Gibraltar fever, Dr. Gilkrest on the | 14 | 224 |
| Gibraltar fever; code sanitaire | 14 | 246 |
| Gibson, Dr. on mercurial fumigation, | 3 | 568 |
| Gilders, diseases of | 18 | 105 |
| Gilkrest, Dr. on yellow fever | 18 | 362 |
| Gland, parotid, extirpation of | 1 | 216 |
| Gland, thyroid, extirpation of | 1 | 472 |
| Glanders, on the cure of | 2 | 249 |
| Glanders, the human subject infected by the matter of, some cases, | 5 | 280 |
| Glanders in the human subject | 14 | 108 |
| Glanders in the human subject | 20 | 10 |
| Glans-penis, inflammation of the | 15 | 413 |
| Glasgow; regulations of this university | 6 | 294 |
| Glasgow Royal Infirmary, report from | 9 | 564 |
| Glasgow, regulations, &c. respecting degrees | 11 | 73 |
| Glasgow Infirmary, Dr. Buchanan's history | 18 | 559 |
| Glasgow Infirmary, cases from | 19 | 249 |
| Glasgow, diseases of | 20 | 158 |
| Glaucoma, Mr. Mackenzie on | 13 | 491 |
| Gleet, Dr. M'Knight on | 9 | 194 |
| Globus antiperistalticus, M. Troilet on | 4 | 510 |
| Glossitis, Dr. Hosack on | 2 | 55 |
| Glossitis cured by incisions | 7 | 411 |
| Glossitis, idiopathic, cases of | 15 | 154 |
| Glossitis, Cyclopædia article on | 18 | 234 |
| Glottis, fatal affection of | 2 | 458 |
| Glottis, fatal case of œdema of the | 8 | 175 |
| Glottis, spasm of, Dr. Marsh on | 14 | 366 |
| Glottis, Mr. Fletcher on spasm of the | 15 | 378 |
| Gluteal aneurism | 18 | 476 |
| Gluteal artery, wound and ligature of, | 20 | 165 |
| Godman, Dr. on tight lacing | 12 | 512 |
| Goethe, last illness of | 20 | 499 |
| Goitre, efficacy of iodine in | 2 | 229 |
| Goitre, its etiology | 2 | 442 |
| Goitre, its etiology | 3 | 228 |
| Goitre | 14 | 453 |
| Goitre in the plains of the Andes, Humboldt on | 18 | 520 |
| Goitre, different sorts of | 19 | 498 |
| Gold & silver, workers in, diseases of, | 18 | 107 |
| Gold-headed cane, memoirs of. | 7 | 38 |
| Gondret, Dr. on cauterization in cataract | 5 | 177 |
| Gonorrhœa, cubebs in | 1 | 228 |
| Gonorrhœa, copaiba in | 3 | 152 |
| Gonorrhœa, case of, treated by iodine | 3 | 279 |
| Gonorrhœa, treatment of | 3 | 456 |
| Gonorrhœa, Mr. B. Bell's views of | 4 | 535 |
| Gonorrhœa—purulent opththalmia | 10 | 528 |
| Gonorrhœa, Mr. Travers on | 13 | 162 |
| Gonorrhœa, nitrate of silver in | 13 | 424 |
| Gonorrhœa and its consequences | 15 | 221 |
| Gonorrhœa combined with syphilis | 15 | 232 |
| Gonorrhœa in females | 17 | 162 |
| Gonorrhœa, Mr. Russell on | 19 | 172 |
| Gonorrhœa, communicated from the stomach | 19 | 498 |

| | Vol. | Page |
|---|---|---|
| Gonorrhœa, cases of | 19 | 549 |
| Gonorrhœa, cases of, by Mr. Johnson, | 20 | 269 |
| Gonorrhœa, simple treatment of | 20 | 269 |
| Gonorrhœal ophthalmia, case of | 11 | 506 |
| Gonorrhœal rheumatism | 12 | 548 |
| Gonorrhœal inflammation of the eye, | 14 | 82 |
| Gonorrhœal ophthalmia, treatment | 14 | 88 |
| Gonorrhœal ophthalmia, cases of | 14 | 91 |
| Gonorrhœal ophthalmia and rheumatism | 15 | 224 |
| Gooch, Dr. on the diseases of women | 11 | 289 |
| Gooch, Dr. on the disorders of the mind | 11 | 359 |
| Gooch, Dr. on the irritable uterus | 11 | 404 |
| Gooch, Dr. on pregnancy | 12 | 42 |
| Gooch, Dr. on pseudo-congestion of the brain in children | 12 | 75 |
| Gooch, Dr. on uterine hæmorrhage | 12 | 194 |
| Gooch, Dr. on polypus uteri | 12 | 321 |
| Gooch, Dr. his dream | 13 | 478 |
| Gooch, Dr. notice of him | 13 | 490 |
| Good, Dr. his study of medicine | 4 | 387 |
| Goodlad, Dr. on croup | 15 | 241 |
| Gorget, objections to, in lithotomy | 1 | 209 |
| Gout, Dr. Hosack on | 2 | 58 |
| Gout, anomalous, M. Bayle | 2 | 187 |
| Gout flying to the stomach, curious case | 4 | 42 |
| Gout, ancient treatment of | 4 | 46 |
| Gout, Dr. Scudamore's mode of treating | 4 | 48 |
| Gout of the stomach, fatal case of | 4 | 142 |
| Gout treated by hot water | 8 | 221 |
| Gout, fatal case of | 15 | 520 |
| Gout of the testis | 19 | 174 |
| Gracchus, his insane letter on anatomy | 11 | 529 |
| Graduates of Scotland—degraded in England | 5 | 636 |
| Graefe, Baron, his surgical report | 20 | 218 |
| Graefe, Baron, his new ligature instrument | 20 | 218 |
| Graefe, Baron, his treatment of polypi | 20 | 219 |
| Graefe, Baron, his treatment of fistula ani | 20 | 219 |
| Graefe, Baron, his treatment of congenital omphalocele | 20 | 221 |
| Grant, Dr. his introductory lecture | 20 | 152 |
| Granville, Dr. his case of indigestion, | 4 | 566 |
| Granville, Dr. on mummies | 5 | 46 |
| Granville, Dr. on gastrotomy | 5 | 576 |
| Granville, Dr. on disinfecting liquids, | 6 | 629 |
| Granville, Dr. illustrations of abortion | 19 | 130 |
| Grave, fatal effects from opening a | 2 | 202 |
| Gravel, M. Majendie on | 18 | 49 |
| Gravel, formation of | 18 | 50 |
| Gravel, red | 18 | 51 |
| Gravel, red, causes of | 18 | 52 |
| Gravel, white | 18 | 53 |
| Gravel, hairy | 18 | 54 |
| Gravel, grey | 18 | 54 |
| Gravel, yellow | 18 | 54 |
| Gravel, transparent | 18 | 55 |
| Gravel, peculiar causes of | 18 | 55 |
| Gravel, symptoms of | 18 | 56 |
| Gravel, treatment of | 18 | 56 |
| Graves, Dr. on free acid in the stomach | 2 | 145 |
| Graves, Dr. on the effects of posture, | 15 | 152 |
| Graves, Dr. on various diseases | 17 | 458 |
| Graves, Dr. on periostitis | 18 | 481 |
| Graves, Dr. on various diseases | 19 | 145, 439 |
| Graves on hydrophobia | 19 | 451 |
| Graves, Dr. on German literature | 19 | 493 |
| Graves, Dr. on the secretion of air | 20 | 442 |
| Greatrex, Mr. his letter on Dr. Stevens | 16 | 295 |
| Green, Mr. his lectures at the college | 1 | 251 |
| Green tea, Mr. Newnham on | 7 | 143 |
| Green, Mr. his taliacotian operation, | 10 | 571 |
| Green, Mr. on distinction without separation | 15 | 161 |
| Green, Mr. his plan of reform | 15 | 168 |
| Green, Mr. on fracture of the cervix femoris | 17 | 150 |
| Greenhow, Mr. on medical remuneration | 2 | 246 |
| Greenhow, Mr. on cholera | 16 | 555 |
| Gregory, Dr. his small-pox report | 2 | 551 |
| Gregory's, Dr. practice of physic | 5 | 408 |
| Gregory, Dr. on vaccination | 6 | 192 |
| Gregory, Dr. his case of hydrophobia, | 6 | 347 |
| Gregory, Dr. on vaccination | 7 | 459 |
| Gregory, Dr. his practice of physic | 10 | 115 |
| Gregory's Conspectus, selections from | 16 | 513 |
| Grinders' asthma, Dr. Knight on the, | 14 | 202 |
| Groin, scirrhous testicle in the | 7 | 239 |
| Groin, cases of obstinate ulceration in the | 18 | 282 |
| Grossesse apparente, ou fausse | 20 | 114 |
| Guaco, Mr. Hawkins on | 14 | 571 |
| Guersent, M. on the diseases of children | 13 | 552 |
| Guilbert, M. on leeching the os uteri, | 5 | 508 |
| Guinea-worm, Clot Bey on the | 18 | 483 |
| Guinea-worm within the hip-joint | 19 | 501 |
| Gullet, Dr. Monro on the spasm of | 5 | 500 |
| Gums, diseases of the | 11 | 412 |
| Gun-shot wound, severe case of | 6 | 251 |
| Gun-shot wound of scalp | 15 | 154 |
| Gun-shot wounds, enlargements of | 16 | 50 |
| Gun-shot wound, effects of, on memory | 19 | 214 |
| Guthrie, Mr. on sloughing of bladder | 1 | 235 |
| Guthrie, Mr. on cataract | 1 | 396 |
| Guthrie, Mr. his mode of amputating at the hip | 3 | 552 |
| Guthrie, Mr. and Professor Walther, | 3 | 553 |
| Guthrie, Mr. on fractures of the femur | 4 | 540 |
| Guthrie, Mr. on phlebitis | 5 | 562 |
| Guthrie, Mr. on gun-shot wounds | 7 | 398 |
| Guthrie, Mr. his use of the chlorurets | 8 | 184 |

| | Vol. | Page |
|---|---|---|
| Guthrie, Mr. his report | 9 | 569 |
| Guthrie, Mr. his letter on anatomy | 10 | 355 |
| Guthrie, Mr. on cataract & glaucoma, | 10 | 472 |
| Guthrie, Mr. on muscæ volitantes | 11 | 184 |
| Guthrie, Mr. his lectures at the College of Surgeons | 11 | 225 |
| Guthrie, Mr. on injuries of the head, | 11 | 283 |
| Guthrie, Mr. on the ol. terebinth. in iritis | 11 | 525 |
| Guthrie, Mr. on oil of turpentine | 12 | 232 |
| Guthrie, Mr. his Hunterian oration | 12 | 528 |
| Guthrie, Mr. on the anatomy bill | 12 | 573 |
| Guthrie, Mr. on the arteries | 13 | 133 |
| Guthrie, Mr. on diseases of arteries | 13 | 350 |
| Guthrie, Mr. his ligature of the iliac, | 16 | 516 |
| Guthrie, Mr. on herniæ | 19 | 471 |
| Guthrie, Mr. his introductory lecture | 20 | 153 |
| Guthrie, Mr. case of ligature of the common iliac | 20 | 278 |
| Guthrie, Mr. on stricture | 20 | 548 |
| Guy's Hospital, resolutions of the students at | 10 | 305 |
| Guy's Hospital, anatomical catalogue of | 13 | 367 |
| Gymnastics, observations on their effects | 8 | 159 |
| Gymnastics, description of them as practised here | 8 | 162 |

**H**

| | Vol. | Page |
|---|---|---|
| Habits, their influence on constitution | 3 | 195 |
| Hackan, Dr. his report on fever | 18 | 149 |
| Hacket, Dr. his letter to Dr. Johnson, | 16 | 289 |
| Hacket, Dr. his letter respecting Dr. Stevens | 17 | 577 |
| Hackman on softening of the spleen, | 17 | 257 |
| Haddington, cholera at | 16 | 640 |
| Haddington, non-contagiousness of cholera | 16 | 641 |
| Haden, Mr. on new remedies | 1 | 47 |
| Haden, Mr. death of | 1 | 56 |
| Haden, Mr. on inflammatory diarrhœa | 1 | 112 |
| Haden, Mr. on cut throat | 1 | 132 |
| Hæmatemesis, cured by emetics | 2 | 481 |
| Hæmatemesis, quickly fatal case of | 3 | 240 |
| Hæmatemesis, discussion on | 8 | 512 |
| Hæmatemesis, its causes in the liver, | 14 | 548 |
| Hæmatemesis, Dr. Cumming on | 20 | 441 |
| Hæmatocele, Sir A. Cooper on | 14 | 26 |
| Hæmaturia, cured by the sound | 7 | 145 |
| Hæmaturia, Dr. Watson on | 17 | 491 |
| Hæmoptoe treated with tartar-emetic | 19 | 486 |
| Hæmoptysis, large doses of nitre in, | 4 | 211 |
| Hæmoptysis, a forerunner of consumption | 4 | 495 |
| Hæmoptysis, employment of cold in, | 5 | 93 |
| Hæmoptysis, small bleedings in | 15 | 146 |
| Hæmorrhage, a good styptic for | 1 | 142 |
| Hæmorrhage into the bladder | 2 | 224 |
| Hæmorrhage from leech-bites | 5 | 57 |
| Hæmorrhage & suppuration, cases of, | 5 | 125 |
| Hæmorrhage in the brain, cases of, &c. | 9 | 77 |
| Hæmorrhage, M. Amussat's new mode of stopping | 11 | 545 |
| Hæmorrhage, peculiar, from uterus, | 12 | 194 |
| Hæmorrhage, modes of stopping | 13 | 134 |
| Hæmorrhage from the throat | 13 | 144 |
| Hæmorrhage, fatal, from the spleen, | 13 | 227 |
| Hæmorrhage from the throat | 13 | 260 |
| Hæmorrhage, syncope from | 13 | 463 |
| Hæmorrhage, secondary | 13 | 501 |
| Hæmorrhage in lithotomy | 15 | 375 |
| Hæmorrhage after amputations | 17 | 181 |
| Hæmorrhage, secondary, Dr. Buchanan on | 17 | 209 |
| Hæmorrhage, uterine, Mr. Ingleby on | 17 | 214 |
| Hæmorrhage, uterine, Dr. Ramsbotham on | 17 | 298 |
| Hæmorrhage, uterine, Mr. Ingleby on | 17 | 337 |
| Hæmorrhage, in last months of pregnancy | 17 | 347 |
| Hæmorrhage, uterine, consequences of | 17 | 351 |
| Hæmorrhage, from plurality of children | 17 | 352 |
| Hæmorrhage, internal and concealed, | 17 | 357 |
| Hæmorrhage, experiments relating to | 18 | 256 |
| Hæmorrhage, uterine, Dr. R. Lee on | 19 | 67 |
| Hæmorrhage, uterine, case of | 20 | 156 |
| Hæmorrhage, secondary, case of | 20 | 250 |
| Hæmorrhage, secale cornutum in | 20 | 451 |
| Hæmorrhages, ergot of rye in | 15 | 171 |
| Hæmorrhagic state of the intestine | 11 | 100 |
| Hæmorrhagic intestine, symptoms of, | 11 | 105 |
| Hæmorrhagic pleurisy—operation | 11 | 462 |
| Hæmorrhoidal excrescences, Mr. Kirby on | 4 | 358 |
| Hæmorrhoids, Mr. Calvert & others on | 2 | 273 |
| Hæmorrhoids, French treatment of | 10 | 527 |
| Hahnemann, his doctrine homoiopathique | 17 | 132 |
| Half-blindness, curious state of | 3 | 201 |
| Halford, Sir H. his oration | 3 | 586 |
| Halford, Sir Henry, on tic douloureux | 9 | 185 |
| Halford, Sir Henry, Dr. Burrows' letter to | 13 | 147 |
| Halford, Sir H. on the mental effects of disease | 15 | 197 |
| Halford, Sir H. his essays | 15 | 358 |
| Halford, Sir H. his 1st letter on cholera | 16 | 178 |
| Halford, Sir H. his 2d letter on ditto | 16 | 179 |
| Hall, Dr. Marshall, his Medical Essays | 4 | 341 |
| Hall, Dr. M. on a destructive inflammation of the eye, &c. | 6 | 342 |
| Hall, Dr Marshall, on the diseases of females | 7 | 316 |
| Hall, Dr. Marshall, on female diseases, | 11 | 289 |
| Hall, Dr. on puerperal mania | 11 | 359 |
| Hall, Dr. Marshall, on blood-letting, | 12 | 40 |
| Hall, Dr. on the loss of blood | 13 | 60, 323 |
| Hall, Dr. his experiments on loss of blood | 16 | 364 |
| Hall, Dr. Marshall, on blood-letting, | 17 | 243 |
| Hall, Dr. on loss of blood | 18 | 354 |

| | Vol. | Page |
|---|---|---|
| Halliday, Dr. on facial neuralgia ... | 19 | 421 |
| Hallucinations, cold water for...... | 9 | 203 |
| Hallucinations, Esquirol on........ | 19 | 353 |
| Hamett's valuable report on cholera, | 16 | 305 |
| Hamett, Dr. on cholera at Dantzick, | 18 | 136 |
| Hamilton, Dr. on panic fever ...... | 2 | 148 |
| Hamilton, Mr. on animal effluvia .. | 2 | 202 |
| Hamilton, Dr. on a peculiar sore throat.................... | 6 | 223 |
| Hammick, Mr. on amputations, &c.. | 12 | 405 |
| Hancock, Dr. on external stimulants, | 18 | 145 |
| Hanging, effects of the rope in ..... | 14 | 345 |
| Hanging, effects of, on the brain.... | 16 | 3 |
| Harrison, Dr. his circular on the College .................. | 7 | 268 |
| Harrison, Dr. on spinal diseases.... | 9 | 87 |
| Harrison, Dr. his trial with the College | 9 | 454 |
| Harrison, Dr. on sterility.......... | 14 | 470 |
| Harrogate, Dr. Hunter on waters of, | 15 | 354 |
| Hart, Dr. on the fungating venereal ulcer ........................ | 20 | 167 |
| Harty, Dr. on polypi of the heart .. | 15 | 96 |
| Harvey, memoranda respecting..... | 13 | 498 |
| Harwood, Dr. on the southern coast of England ................ | 10 | 24 |
| Haslam, Dr. his lectures .......... | 8 | 405 |
| Haslam—his *resignation* of the presidential chair .............. | 10 | 496 |
| Hastings, Dr. on empyema ........ | 4 | 557 |
| Hastings, Dr. on mollescence of the lungs ................. | 6 | 517 |
| Hastings, Dr. on the signs of pulmonary tubercles ............ | 10 | 501 |
| Hastings, Dr. his case of ischuria .. | 11 | 274 |
| Hastings, Dr. on use of stethoscope, | 11 | 497 |
| Hastings, Dr. on cysts connected with the liver .............. | 11 | 536 |
| Hawkins, Dr. on antimonial powder, | 1 | 227 |
| Hawkins, Dr. on medical statistics .. | 11 | 420 |
| Hawkins, Mr. on the arrest of hæmorrhage ................. | 18 | 38 |
| Hawkins, Mr. experiments of...... | 18 | 39 |
| Hawkins, Mr. conclusions of ...... | 18 | 41 |
| Hawkins, Mr. on encysted tumors of the liver ................. | 20 | 11 |
| Hawkins, Mr. on hydatidic tumors.. | 20 | 19 |
| Hawkins' Mr. cases of diseased testis, | 20 | 247 |
| Hay, Dr. case of dysphagia ........ | 1 | 300 |
| Hay fever, Dr. Bostock on ........ | 9 | 462 |
| Hayes, Mr. on blighted ovum ...... | 1 | 124 |
| Hayes, Mr. on poisoning by opium.. | 1 | 135 |
| Head, on various injuries of ....... | 1 | 464 |
| Head, case of severe affection of the, | 2 | 490 |
| Head, morbid appearances of, in fever, | 12 | 357 |
| Head, injuries of, report on........ | 13 | 233 |
| Head, on injuries of the........... | 13 | 557 |
| Head, report on injuries of the..... | 14 | 274 |
| Head, M. Larrey on injuries of the.. | 15 | 7 |
| Head, cases of injury of........... | 15 | 505 |
| Head, on injuries of the........... | 15 | 532 |
| Head, severe injury of the ........ | 17 | 538 |
| Head, injuries of the ............. | 18 | 506 |

| | Vol. | Page |
|---|---|---|
| Head, injuries of the............. | 19 | 253 |
| Head, bloody tumors of the........ | 20 | 210 |
| Headach, two kinds of.......... | 6 | 32 |
| Headach, obstinate, removed by erysipelas ................... | 11 | 473 |
| Headach, Mr. Carmichael on ...... | 19 | 464 |
| Headachs, Dr. Vaughan on ........ | 16 | 83 |
| Headachs, divisions of............ | 16 | 85 |
| Headachs in young women ........ | 19 | 145 |
| Head-bones and face, varieties in their anatomy ............... | 9 | 200 |
| Heads of children, often swollen in parturition ................ | 1 | 350 |
| Health, how influenced by education, &c. ................. | 3 | 191 |
| Health in ancient and modern times, | 11 | 420 |
| Health, pilgrim of................ | 19 | 183 |
| Health, effects of misery on........ | 19 | 342 |
| Hearing, sudden loss of .......... | 1 | 180 |
| Hearing & speech restored; an instance | 4 | 138 |
| Hearing, illustrations of the organ of, | 9 | 204 |
| Hearing, Dr. Caswall on .......... | 18 | 537 |
| Hearing through apertures from the trephine ................. | 20 | 516 |
| Heart, Dr. Abercrombie on the .... | 1 | 72 |
| Heart, gangrene of .............. | 1 | 189 |
| Heart, on valvular diseases of the .. | 1 | 193 |
| Heart, malformation of ............ | 1 | 301 |
| Heart, diseased; an important case of | 6 | 48 |
| Heart, an interesting case of disease of | 6 | 215 |
| Heart, Dr. Johnson's case of disease of | 6 | 343 |
| Heart, metastasis of rheumatism to, | 7 | 348 |
| Heart, peculiar case of disease of the, | 8 | 151 |
| Heart and arteries, functional affections of the ................. | 9 | 20 |
| Heart, spasm of the .............. | 9 | 24 |
| Heart diseased or disordered by onanism ................... | 9 | 147 |
| Heart, diseases of its muscular structure .................. | 9 | 388 |
| Heart, diseases of the openings of the | 9 | 393 |
| Heart, neuralgic and sympathetic affections of ................ | 10 | 93 |
| Heart, imperfection of the ........ | 11 | 159 |
| Heart, extensive wound of ........ | 11 | 165 |
| Heart, false aneurism of the........ | 11 | 249 |
| Heart, pus in the cavities of the.... | 11 | 475 |
| Heart, diseased, interesting cases of, | 11 | 517 |
| Heart, report on diseases of the .... | 11 | 556 |
| Heart, great dilatation and hypertrophy of .................. | 11 | 561 |
| Heart, apoplexy of the............ | 12 | 88 |
| Heart, neuralgia of the............ | 12 | 122 |
| Heart, Dr. Williams on the auscultation of the ................ | 12 | 155 |
| Heart, state of, in sleep .......... | 12 | 299 |
| Heart, hypertrophy of right side of.. | 12 | 555 |
| Heart, Dr. Corrigan on the........ | 13 | 122 |
| Heart, diseases of the............. | 13 | 176 |
| Heart, *simple* diseases of the....... | 13 | 317 |
| Heart, Dr. Elliotson on the........ | 13 | 424 |
| Heart, M. Larrey on the ......... | 13 | 535 |

| | Vol. | Page |
|---|---|---|
| Heart, marvellous disease of the.... | 13 | 538 |
| Heart, Dr. Elliotson on the diseases, | 14 | 60 |
| Heart, affections of its lining membrane.... ................ | 14 | 60 |
| Heart, on the sounds of the ....... | 14 | 61 |
| Heart, disease of the auriculo-ventricular openings of .......... | 14 | 64 |
| Heart, remarkable disease of the.... | 14 | 196 |
| Heart, malformation of the........ | 14 | 231 |
| Heart, Dr. Elliotson on diseases of the | 14 | 323 |
| Heart, sounds of, in disease........ | 14 | 325 |
| Heart, diseased, general symptoms of, | 14 | 327 |
| Heart, rupture of the. ............ | 14 | 330 |
| Heart, neuralgia of the............ | 14 | 330 |
| Heart, anomalous state of the...... | 14 | 364 |
| Heart, disease of the.............. | 14 | 521 |
| Heart, motions and sounds of the. .. | 15 | 220 |
| Heart, abstinence in diseases of..... | 15 | 491 |
| Heart, case of ulceration of........ | 15 | 566 |
| Heart, congenital malformations of.. | 16 | 68 |
| Heart, symptoms of malformations of the...................... | 16 | 71 |
| Heart, relative anatomy of the...... | 16 | 265 |
| Heart, Dr. Hope on the action of the, | 16 | 358 |
| Heart, sounds of the, in disease .... | 16 | 362 |
| Heart, on murmurs of the......... | 16 | 364 |
| Heart, inflammation of the ........ | 16 | 366 |
| Heart, abscess of the ............. | 17 | 170 |
| Heart, disease of the.............. | 17 | 175 |
| Heart, purulent cysts in the ....... | 17 | 182 |
| Heart, dilatation of the cavities of .. | 17 | 183 |
| Heart, disease of, unsuspected...... | 17 | 406 |
| Heart, disease of, remarks on...... | 17 | 464 |
| Heart, abscess in the ............. | 18 | 174 |
| Heart, case of wound of .......... | 18 | 453 |
| Heart, new theory of sounds of .... | 18 | 454 |
| Heart; M. Pigeaux's theory ....... | 18 | 455 |
| Heart, spontaneous ruptures of .... | 18 | 455 |
| Heart, malformation of .......... | 18 | 456 |
| Heart, Dr. Billing's theory of its sounds | 18 | 564 |
| Heart, wounds of.................. | 19 | 84 |
| Heart, diseases of................. | 19 | 196 |
| Heart, on the sounds of .......... | 19 | 216 |
| Heart, disease of the, Dr. Jeffreys on, | 19 | 365 |
| Heart, polypous concretions of ..... | 19 | 518 |
| Heart, the bruits of .............. | 19 | 519 |
| Heart, abnormal cavity in ........ | 19 | 528 |
| Heart, fœtal, sounds of............ | 20 | 101 |
| Heart, sounds of, explained by Laennec, Turner, Corrigan, Williams, Pigeaux, Majendie, Rouanet, Carlile, and Hope ........... | 20 | 133, 134 |
| Heart, differences in the fœtal & adult | 20 | 163 |
| Heart, presence of a needle in the .. | 20 | 235 |
| Heart, symptomatic affections of the, | 20 | 494 |
| Heart, fibrinous polypi in cavities of, | 20 | 551 |
| Heat, low power of resisting ...... | 17 | 494 |
| Hectic relieved on the appearance of dropsy ........ ......... | 4 | 133 |
| Heermans, Dr. his case of phlegmatia dolens ........ ....... | 1 | 386 |
| Heiniker, Dr. his complaint answered | 3 | 600 |

| | Vol. | Page |
|---|---|---|
| Helbich, Dr. on the Polish carbuncle, | 11 | 204 |
| Heliotrope, the.................. | 19 | 183 |
| Helleborus albus, for simulating cardiac disease ............... | 11 | 196 |
| Helminthology, M. St. Hilaire on .. | 19 | 527 |
| Helot, the contented ............. | 7 | 583 |
| Hemeralopia, or night blindness.... | 13 | 214 |
| Hemeralopia, Dr. Patterson on ..... | 19 | 266 |
| Hemicrania, cured by acupuncture.. | 3 | 263 |
| Hemicrania, M. Piorry on......... | 16 | 236 |
| Hemicrania, Dr. Bright on ........ | 16 | 334 |
| Hemiplegia—strychnine employed .. | 12 | 239 |
| Hemiplegia, treatment of.......... | 14 | 477 |
| Hemiplegia, intermittent, case of... | 14 | 514 |
| Hemiplegia, with & without visible cause ...................... | 14 | 547 |
| Hemiplegia, ligature of carotid for .. | 15 | 180 |
| Hemiplegia, carotid tied for........ | 18 | 253 |
| Henderson, Dr. his history of ancient and modern wines ...... | 4 | 305 |
| Hennen, Dr. his military surgery.... | 12 | 361 |
| Hennen, Dr. on the Mediterranean.. | 13 | 49 |
| Hennen, Dr. on the state of Gibraltar | 13 | 339 |
| Hennen, Dr. his medical topography, | 14 | 97 |
| Hennen, Dr. on the climate of Malta, | 14 | 336 |
| Henning, Dr. his dissection........ | 2 | 457 |
| Hepatic phthisis ................. | 2 | 184 |
| Hepatic ileus, Dr. Musgrave's paper on | 4 | 248 |
| Hepatic abscess, curious case of .... | 7 | 135 |
| Hepatic tumor, M. Luroth's case .. | 7 | 186 |
| Hepatic diseases, Drs. Graves and Stokes on .................. | 8 | 494 |
| Hepatic disease, curious case of .... | 8 | 560 |
| Hepatic abscess, external pointing of, | 10 | 438 |
| Hepatic abscess, opening into the lungs | 10 | 440 |
| Hepatic abscess—Mr. Waldron's case | 10 | 515 |
| Hepatic phthisis, case of .......... | 11 | 87 |
| Hepatic abscess—curious case...... | 13 | 567 |
| Hepatic dysentery... ............. | 14 | 334 |
| Hepatic abscess, case of .......... | 19 | 540 |
| Hepatitis, its insidious nature...... | 5 | 347 |
| Hepatitis, diagnostic symptoms of .. | 6 | 209 |
| Hepatitis, acute, Mr. Annesley on .. | 9 | 337 |
| Hepatitis, acute, pathology of ..... | 9 | 341 |
| Hepatitis, acute, etiology of........ | 9 | 342 |
| Hepatitis, acute, treatment of...... | 9 | 344 |
| Hepatitis, chronic, Mr. Annesley on, | 10 | 309 |
| Hepatitis, chronic, treatment of .... | 10 | 317 |
| Hepatitis, acute, "morbid matters," | 11 | 167 |
| Hepatitis, hysteria mistaken for .... | 12 | 240 |
| Hepatitis, ending in abscess ....... | 17 | 182 |
| Hepatitis, different forms of ....... | 19 | 416 |
| Hepatization of the lungs—what.... | 2 | 72 |
| Hepatization, objections to the term, | 3 | 509 |
| Hepatization, red, case of ......... | 3 | 518 |
| Herbarium, formation of an ....... | 9 | 552 |
| Hereditary nature of diseases ...... | 3 | 187 |
| Hereditary nature of bronchocele .. | 4 | 4 |
| Hereditary predisposition to madness | 10 | 11 |
| Hermaphrodism, M. Hilaire on .... | 19 | 231 |
| Hermaphrodism, M. Dutrochet on.. | 19 | 233 |
| Hermaphrodism, extraordinary case of | 19 | 237 |

| | Vol. | Page |
|---|---|---|
| Hermaphrodism, M. Castel on | 20 | 236 |
| Hernia, Mr. Yeatman's treatment of, | 2 | 467 |
| Hernia humoralis, cases of, cured by iodine | 3 | 278 |
| Hernia, delay in operating for | 4 | 233 |
| Hernia, strangulated, case of | 4 | 550 |
| Hernia, cases of, by Messrs. Shaw and Boyle | 5 | 569 |
| Hernia, cases of, at St. Thomas's | 6 | 609 |
| Hernia, with retention of urine | 7 | 275 |
| Hernia, cases of | 7 | 521 |
| Hernia, strangulated, cases of, with remarks | 7 | 523 |
| Hernia, curious case of, by Mr. Earle, | 8 | 170 |
| Hernia, curious case of | 9 | 159 |
| Hernia complicated with hydrocele, &c. | 9 | 160 |
| Hernia, case of, with Dr. Ballingall's remarks | 9 | 188 |
| Hernia, femoral, fatal case of | 9 | 556 |
| Hernia—operation—difficult reduction of the gut | 10 | 475 |
| Hernia, adherent, Mr. Stephens on | 11 | 109 |
| Hernia, inflamed, Mr. Stephens on | 11 | 113 |
| Hernia, suggestion for radical cure of, | 11 | 115 |
| Hernia; testicles absent from scrotum | 11 | 263 |
| Hernia, curious cases of | 11 | 547 |
| Hernia, inguinal, new species of | 12 | 180 |
| Hernia of the bladder, case of | 12 | 444 |
| Hernia, on the diagnosis of | 12 | 459 |
| Hernia, umbilical, remarkable case of, | 12 | 463 |
| Hernia humoralis, symptoms of | 13 | 389 |
| Hernia, Mr. Geoghegan on | 14 | 149 |
| Hernia, rapidly fatal case of | 15 | 153 |
| Hernia—Mr. Egg's trusses | 15 | 516 |
| Hernia cerebri, cases of | 16 | 518 |
| Hernia, cases of, by Mr. Clement | 17 | 45 |
| Hernia, entero-vaginal, cases of | 17 | 253 |
| Hernia, without external swelling | 18 | 463 |
| Hernia, cord inflamed, mistaken for, | 18 | 468 |
| Hernia with a double sac | 19 | 283 |
| Hernia, Mr. James on | 19 | 368 |
| Hernia, umbilical, case of | 19 | 453 |
| Hernia diaphragmatica, case of | 19 | 513 |
| Hernia humoralis, cases of | 19 | 550 |
| Hernia, strangulated, Mr. Key on | 20 | 80 |
| Hernia, umbilical, of the gravid uterus | 20 | 164 |
| Hernia, femoral, thickness of sac in, | 20 | 204 |
| Herniæ, curious treatment of | 2 | 211 |
| Herniæ of children, leeches in the | 10 | 166 |
| Herniæ of the muscles, M. Dupuytren on | 11 | 566 |
| Herniæ, Mr. Guthrie on | 19 | 470 |
| Herpes genitalis | 3 | 556 |
| Herpes Zoster, lunar caustic in | 5 | 573 |
| Herpes, M. Biett on | 11 | 568 |
| Herpes, M. Biett on | 16 | 392 |
| Hesse, Dr. on extirpation of the uterus | 8 | 195 |
| Hetling, Mr. on osteo-sarcoma of jaw | 19 | 261 |
| Hetling, Mr. his introductory lecture | 20 | 181 |
| Heurteloup, Baron, his instruments, | 11 | 573 |
| Heurteloup, Baron, on lithotrity | 16 | 134 |
| Heurteloup, M. his instruments | 16 | 140 |

| | Vol. | Page |
|---|---|---|
| Hewett, Dr. on follicular ulceration, | 5 | 557 |
| Hewett, Dr. on the treatment of fever | 6 | 198 |
| Hewlett, Mr. his case of ovarian disease | 18 | 46 |
| Hewson, Mr. on ophthalmia | 2 | 305 |
| Hibernation, Dr. Edwards on | 18 | 13 |
| Hibernation, facts relating to | 18 | 15 |
| Hibernation, Dr. M. Hall on | 18 | 497 |
| Hibernation, respiration during | 18 | 498 |
| Hibernation, irritability, &c. during, | 18 | 499 |
| Hiccup lasting 12 days, case of | 7 | 196 |
| Higginbottom, Mr. his Essay on Lunar Caustic | 4 | 372 |
| Higginbottom, Mr. on nitrate of silver | 10 | 444 |
| High operation for the stone, case of, | 10 | 569 |
| Highlands, salutarium in | 19 | 160 |
| Hindoo treatment of diseased spleen, | 11 | 317 |
| Hints to medical officers, Mr. Marshall's | 9 | 90 |
| Hip-dislocation, Dr. Hunter on | 1 | 88 |
| Hip-joint amputation, successful | 2 | 225 |
| Hip-joint amputation, objections to, | 3 | 49 |
| Hip-joint, congenital dislocation of | 6 | 544 |
| Hip-joint, disease of the | 7 | 254 |
| Hip-joint, successful amputation of, | 7 | 418 |
| Hip-joint, injury of the | 8 | 438 |
| Hip-joint, Mr. Bell on the | 8 | 444 |
| Hip-joint, successful amputation at, | 9 | 502 |
| Hip-joint, amputation at the | 15 | 513 |
| Hip-joint, amputation at | 19 | 195 |
| Hippocrates, notices respecting | 15 | 395 |
| *Hirsutus tubulus*—description of the disease | 9 | 205 |
| Histoire des Marais, M. Monfalcon's, | 3 | 317 |
| History of medicine, Mr. Moir's | 15 | 394 |
| History of medicine, Mr. Hamilton's, | 15 | 394 |
| Hitchcock, Professor, on dyspepsia | 15 | 571 |
| Hodgkin, Dr. on intermissions of the pulse | 10 | 562 |
| Hodgkin, Dr. on adventitious structures | 13 | 44 |
| Hodgkin, Dr. his catalogue | 13 | 367 |
| Hohenloe, Prince, his miracles | 1 | 176 |
| Hohenloe in Panton-square! | 6 | 253 |
| Holland, Dr. on the spleen, &c. | 15 | 47 |
| Holmes, Dr. on malformation of the heart | 1 | 301 |
| Holscher, Dr. his extirpation of the uterus | 3 | 266 |
| Home, Sir E. on tumors | 14 | 41 |
| Homer, Dr. his case of gastritis | 13 | 158 |
| Homicidal monomania, curious cases | 9 | 244 |
| Homicide & infanticide, propensity to | 6 | 226 |
| Homicide; case of Harriette Cornier, | 6 | 482 |
| Homicide and matricide, cases of | 6 | 489 |
| Homoiopathic doctrine of Hahnemann | 19 | 429 |
| Homoiopathy, wonders of | 7 | 432 |
| Homoiopathy in Egypt | 19 | 194 |
| Hoo Loo, operation on | 15 | 150 |
| Hood, Dr. on lunar caustic | 11 | 211 |
| Hook, the inventor of auscultation | 13 | 426 |
| Hooping-cough, Dr. Webster on | 1 | 192 |

| | Vol. | Page |
|---|---|---|
| Hooping-cough, Dr. Gregory's opinions on | 5 | 409 |
| Hooping-cough, Dr. Dawson's opinions on | 5 | 413 |
| Hooping-cough, dissection | 6 | 612 |
| Hooping-cough, case of | 6 | 613 |
| Hooping-cough, Dr. Palmer on | 12 | 308 |
| Hooping-cough, Mr. Alcock on | 13 | 29 |
| Hooping-cough, pathology of | 14 | 142 |
| Hooping-cough, M. Bland on | 15 | 480 |
| Hooping-cough, Dr. Autenrieth's treatment of | 18 | 527 |
| Hooping-cough treated by tartar-emetic | 19 | 244 |
| Hope, Dr. on diseases of the heart | 16 | 262, 356 |
| Hope, Dr. on aortic aneurism | 16 | 463 |
| Hope, Dr. on morbid anatomy | 18 | 284, 367 |
| Hope, Dr. on morbid anatomy | 19 | 329 |
| Hope, Dr. on the intestinal glands in cholera | 20 | 67 |
| Hope's, Dr. illustrations of morbid anatomy | 20 | 296 |
| Hôpital de la Charité | 3 | 98 |
| Hôpital des Enfans, account of it | 3 | 344 |
| Hopkins, Dr. his obstetric apparatus, | 7 | 582 |
| Horner, Dr. on the mucous membrane | 8 | 361 |
| Horse-exercise, Dr. Parry's opinion of | 3 | 193 |
| Hosack, Dr. on phlegmatia dolens | 1 | 387 |
| Hosack, Dr. miscellaneous papers | 2 | 53 |
| Hospital reports of M. Laennec | 2 | 240 |
| Hospital reports, by M. Cayol | 2 | 460 |
| Hospital reports by Dr. Hosack | 2 | 482 |
| Hospital reports, M. Recamier's | 3 | 217 |
| Hospital reports, from l'Hôtel Dieu, | 3 | 524 |
| Hospital surgeons, in England | 3 | 584 |
| Hospital physicians and surgeons, their duty | 4 | 197 |
| Hospital reports, Dr. Carter's | 5 | 195 |
| Hospital reports, Dr. Bland's | 5 | 567 |
| Hospital reports, by M. Lisfranc | 5 | 570 |
| Hospital reports, observations on | 7 | 584 |
| Hospital report in the Lancet—unjustifiable conduct | 9 | 164 |
| Hospital gangrene, Dr. Boggie on | 9 | 323 |
| Hospital gangrene, varieties of | 9 | 324 |
| Hospital gangrene, causes of | 9 | 325 |
| Hospital gangrene, treatment of | 9 | 329 |
| Hospital diseases | 9 | 564 |
| Hospital gangrene, cases of | 10 | 167 |
| Hospital reports and elections | 10 | 546 |
| Hospital gangrene, mode of treating, | 10 | 589 |
| Hospital gangrene, Dr. Adam on | 11 | 371 |
| Hospital gangrene, treatment of | 11 | 373 |
| Hospital facts and observations, Dr. Bardsley's | 12 | 129 |
| Hospital for children at Paris | 12 | 541 |
| Hospital facts and observations | 13 | 333 |
| Hospital gangrene, M. Larrey on | 15 | 4 |
| Hospital physicians, remarks on | 15 | 289 |
| Hospital reports, Mr. Crampton on, | 17 | 507 |

| | Vol. | Page |
|---|---|---|
| Hospital reports, further remarks on, | 17 | 510 |
| Hospital reports, remarks on | 17 | 527 |
| Hospital, St. George's, cases from | 19 | 274 |
| Hospitals and infirmaries, advantages of | 12 | 146 |
| Hospitals, military, history of | 18 | 431 |
| Hôtel Dieu, sketch of it | 3 | 96 |
| Hôtel Dieu—M. Recamier's reports, | 4 | 209 |
| Hôtel Dieu, clinical report from | 9 | 225 |
| Hough, Mr. on the Neilgherry | 13 | 308 |
| Hour-glass contraction of the bladder | 5 | 503 |
| Houston, Dr. on the rectal mucous membrane | 14 | 213 |
| Houston, Dr. his pathological observations | 14 | 344 |
| Howison, Dr. his case of enlarged heart | 3 | 536 |
| Howship, Mr. on the spine | 1 | 8 |
| Howship, Mr. on indigestion | 4 | 34 |
| Howship, Mr. his case of mollities ossium | 5 | 405 |
| Howship, Mr. his case of chronic tumor | 15 | 498 |
| Howship, Mr. his case of cerebral disease | 18 | 44 |
| Hufeland, M. on diabetes | 9 | 436 |
| Hufeland on the influenza | 19 | 485 |
| Human pregnancy, evidence on the duration of | 5 | 170 |
| Human anatomy, M. Cloquet's, by Dr. Knox | 11 | 117 |
| Human mind, differences of the | 13 | 10 |
| Human horns | 13 | 567 |
| Human subject, glanders in the | 14 | 108 |
| Humboldt, M. on goitre in the Andes | 18 | 520 |
| Humerus, excision of its head | 6 | 235 |
| Humerus, fracture of the neck of the | 10 | 136 |
| Humerus, dislocation of, into the axilla | 18 | 165 |
| Humerus, excised and amputated; cases | 18 | 274 |
| Humoral pathology, M. Stultz on the | 8 | 534 |
| Humoral pathology, reflections on the | 9 | 15 |
| Humoral pathology, remarks on the, | 9 | 134 |
| Humoralists, difficulties for the | 11 | 345 |
| Humors of the eye, nutrition, &c. of, | 20 | 505 |
| Hunger-cure successfully employed, | 5 | 527 |
| Hunger, a cause of gastritis | 7 | 52 |
| Hunger and thirst; state of alimentary canal | 14 | 437 |
| Hunt, Mr. his operation for ptosis | 14 | 243 |
| Hunter, Dr. Adam, on dislocations | 1 | 88 |
| Hunter, Mr. his evidence on Donellan's trial | 3 | 183 |
| Hunter, Dr. his influence on midwifery | 3 | 402 |
| Hunter, Dr. life of | 13 | 488 |
| Hunterian Museum, Dr. Ager's circular | 6 | 283 |
| Hunterian Museum, Dr. M'Cleod's circular | 6 | 285 |
| Hunterian Museum, remarks on the regulations of admission to it | 6 | 288 |

| | Vol. | Page |
|---|---|---|
| Hunterian oration, Mr. Guthrie's .. | 12 | 528 |
| Hunterian operation inapplicable to wounded arteries ............ | 13 | 138 |
| Husbandmen, healthiness of ...... | 18 | 102 |
| Huskisson, Mr. on his death....... | 14 | 198 |
| Hutchinson, Dr. on croton oil ..... | 19 | 448 |
| Hutchison's, Mr.A.Copland,operation | 2 | 224 |
| Hutchison, Mr. on imperforate anus, | 5 | 511 |
| Hutchison, Mr. on incisions in erysipelas ..................... | 9 | 383 |
| Hyaloid membrane, inflammation of, | 15 | 73 |
| Hydatid formations, Dr. Bailey's cases of ........................ | 4 | 524 |
| Hydatid cyst, immense, in the abdomen ......................... | 9 | 195 |
| Hydatid cyst in the liver, cases of .. | 9 | 499 |
| Hydatid tumor of the breast ...... | 12 | 527 |
| Hydatid disease of testis ...... .... | 13 | 400 |
| Hydatid disease of the testis ........ | 14 | 200 |
| Hydatid cysts in abdomen and thorax | 18 | 514 |
| Hydatid tumors of the wrist ..... | 20 | 241 |
| Hydatidic disease of the testicle .... | 20 | 245 |
| Hydatids of the liver, cured by an operation ..................... | 8 | 459 |
| Hydatids of the placenta, M. Cruveilhier on.......................... | 12 | 80 |
| Hydatids of the uterus............ | 15 | 25 |
| Hydatids, conversion of ........... | 19 | 201 |
| Hydrarthrosis, Dr. Vilette on ...... | 8 | 506 |
| Hydrocele ; Baron Larrey's treatment successful ............... | 8 | 501 |
| Hydrocele cured by astringents .... | 9 | 172 |
| Hydrocele, difficulty of diagnosis of.. | 9 | 279 |
| Hydrocele, sacculated, Mr. Guthrie's remarks on .............. | 10 | 231 |
| Hydrocele, excision of the tunica vaginalis ........................ | 10 | 238 |
| Hydrocele—varieties of .......... | 10 | 539 |
| Hydrocele, injection of, followed by peritonitis, &c............... | 11 | 174 |
| Hydrocele of spermatic cord, with peritonitis ...................... | 11 | 178 |
| Hydrocele, adipocire in .......... | 11 | 533 |
| Hydrocele, Mr. Symes on.......... | 12 | 192 |
| Hydrocele, Sir A. Cooper on....... | 14 | 14 |
| Hydrocele, modes of treatment for.. | 14 | 18 |
| Hydrocele of the spermatic cord.... | 14 | 22 |
| Hydrocele and hæmatocele cured.... | 15 | 522 |
| Hydrocele, diffused, of the cord .... | 17 | 529 |
| Hydrocele, spontaneous cure of .... | 18 | 489 |
| Hydrocephalic fever, Dr. Evanson on | 1 | 364 |
| Hydrocephalic effusion, is it removable? ................... | 5 | 357 |
| Hydrocephalic water, analysis of.... | 15 | 316 |
| Hydrocephalus, operated on by tapping ......................... | 1 | 214 |
| Hydrocephalus, with bifid spine .... | 1 | 297 |
| Hydrocephalus internus ........... | 1 | 479 |
| Hydrocephalus cured by mercury... | 2 | 501 |
| Hydrocephalus, drawing off water in, | 3 | 306 |
| Hydrocephalus acutus, Dr. Jadelot's treatment of ................ | 3 | 348 |

| | Vol. | Page |
|---|---|---|
| Hydrocephalus, Dr. H. Davies on .. | 3 | 571 |
| Hydrocephalus, instance of......... | 4 | 145 |
| Hydrocephalus tapped, Mr.Sym's case | 4 | 236 |
| Hydrocephalus cured by pressure, supposed case of ............ | 4 | 262 |
| Hydrocephalus internus, nature and treatment of .................. | 4 | 408 |
| Hydrocephalus, some cases of...... | 5 | 355 |
| Hydrocephalus chronicus, Mr. Miller's case .......... ......... | 6 | 81 |
| Hydrocephalus, Dr. Monro on....... | 8 | 385 |
| Hydrocephalus, Dr. Mills on ...... | 10 | 28 |
| Hydrocephalus, acute, description of, | 10 | 30 |
| Hydrocephalus, chylopoietics, disorder in.......................... | 10 | 33 |
| Hydrocephalus, diagnosis of.. ..... | 10 | 35 |
| Hydrocephalus, treatment of ...... | 10 | 37 |
| Hydrocephalus, chronic, symptoms of | 10 | 41 |
| Hydrocephalus, chronic, treatment of | 10 | 41 |
| Hydrocephalus, prevention of...... | 10 | 43 |
| Hydrocephalus, encysted .......... | 14 | 346 |
| Hydrocephalus, Dr. Bright on...... | 15 | 306 |
| Hydrocephalus, treatment of....... | 15 | 312 |
| Hydrocephalus, chronic, case of .... | 15 | 314 |
| Hydrocephalus, tapping for ........ | 15 | 317 |
| Hydrocephalus, cured by puncturing, | 17 | 187 |
| Hydrocephalus, spurious, with psoas abscess..................... | 18 | 160 |
| Hydrocephalus, Dr. Graves on...... | 19 | 445 |
| Hydrochloric acid, its powers in diptheritis ..................... | 5 | 429 |
| Hydrocyanic acid, its dose & strength, | 3 | 149 |
| Hydrocyanic acid in dyspnœa....... | 9 | 173 |
| Hydrocyanic acid, properties of .... | 16 | 378 |
| Hydrometra and dry gangrene, Dr. Thomson's case of ........... | 6 | 340 |
| Hydrometra, case of............... | 20 | 117 |
| Hydrophia, dissections of ......... | 1 | 185 |
| Hydrophobia, supposed fatal case of, | 2 | 497 |
| Hydrophobia prevented by mercury, | 2 | 499 |
| Hydrophobia, prevented by the actual cautery ................. | 3 | 299 |
| Hydrophobia, state of the nerves in, | 3 | 534 |
| Hydrophobia, Dr. Booth on......... | 3 | 571 |
| Hydrophobia, Dr. Brandreth's case of | 3 | 575 |
| Hydrophobia...................... | 4 | 263 |
| Hydrophobia, preventions of ...... | 4 | 265 |
| Hydrophobia spontaneous, Dr. Whymper's case of .......... | 4 | 518 |
| Hydrophobia simulata, curious case, | 5 | 261 |
| Hydrophobia, doubtful case of...... | 5 | 564 |
| Hydrophobia, some cases of ....... | 7 | 236 |
| Hydrophobia, amputation of the limb in ......................... | 7 | 237 |
| Hydrophobia, supposed case of .... | 8 | 191 |
| Hydrophobia cured by venesection.. | 9 | 469 |
| Hydrophobia, Murray and Youatt on, | 13 | 433 |
| Hydrophobia, M. Larrey on......... | 15 | 2 |
| Hydrophobia, Dr. Bright on........ | 16 | 349 |
| Hydrophobia, injection into veins for | 17 | 145 |
| Hydrophobia, case of recovery from, | 19 | 451 |
| Hydrophthalmia, Mr. Middlemore on | 19 | 259 |

| | Vol. | Page |
|---|---|---|
| Hydro-pneumo-thorax, case of .... | 8 | 462 |
| Hydrorachis, congenital ........... | 1 | 5 |
| Hydro-rachitis, case of............ | 14 | 162 |
| Hydro-rachitis, Mr. Stafford on .... | 17 | 21 |
| Hydrostatic test .................. | 1 | 344 |
| Hydrostatic bed, Dr. Arnott's...... | 17 | 564 |
| Hydrothorax, Drs. Graves & Stokes on | 9 | 513 |
| Hydrothorax, curious case of ...... | 13 | 569 |
| Hydrothorax, paracentesis thoracis.. | 15 | 149 |
| Hymen, imperforate, effects of ..... | 19 | 190 |
| Hyosciamus and camphor, mode of exhibiting ............... | 6 | 227 |
| Hyosciamus, effects of an over-dose, | 6 | 242 |
| Hyosciamus, seeds of, poisoning from | 20 | 205 |
| Hyperæmia, M. Andral on.......... | 16 | 87 |
| Hyperæmia, active or sthenic ...... | 16 | 88 |
| Hyperæmia, secondary, characters of | 16 | 89 |
| Hyperæmia, bloodletting & emetic-tartar in.................... | 16 | 91 |
| Hyperæmia, state of the capillaries in | 16 | 92 |
| Hyperæmia, asthenic.............. | 16 | 94 |
| Hyperæmia, mechanical........... | 16 | 97 |
| Hyperæmia, cadaveric ............. | 16 | 98 |
| Hyperæmiæ, co-existence of several.. | 16 | 91 |
| Hypertrophia cerebri, case of ...... | 4 | 523 |
| Hypertrophia cordis, remarkable case | 9 | 537 |
| Hypertrophies of the heart, real and apparent ..................... | 4 | 199 |
| Hypertrophy of the heart ......... | 7 | 199 |
| Hypertrophy of the brain, M. Laennec on ...................... | 10 | 565 |
| Hypertrophy of left ventricle—ulcers of ileum, curious case of .. | 11 | 241 |
| Hypertrophy of the heart in apoplexy, | 11 | 458 |
| Hypertrophy of the heart, extensive, case of ................ | 11 | 466 |
| Hypertrophy of the left ventricle, cases of ..................... | 11 | 557 |
| Hypertrophy, enormous, of the heart, | 12 | 463 |
| Hypertrophy of the stomach ....... | 13 | 491 |
| Hypertrophy of the heart.......... | 14 | 323 |
| Hypertrophy of the mammæ ...... | 19 | 495 |
| Hypertrophy of the mamma, cases of | 20 | 464 |
| Hypochondriasis, marked case of ... | 6 | 466 |
| Hypochondriasis, cured by ague, case of ...................... | 8 | 186 |
| Hypochondriasis from chronic inflammation of the bowels....... | 11 | 150 |
| Hypochondriasis, Mr. Annesley on.. | 11 | 510 |
| Hypochondriasis, remarkable case of, | 12 | 509 |
| Hypochondriasis, Dr. Reid on...... | 13 | 83 |
| Hysteralgia, on.................. | 19 | 162 |
| Hysteria, its Protean forms........ | 3 | 385 |
| Hysteria, various forms of ........ | 7 | 329 |
| Hysteria, curious case of.......... | 10 | 461 |
| Hysteria, cases of and remarks on .. | 12 | 186 |
| Hysteria, Mr. Tate on ............ | 13 | 30 |
| Hysteria, heroic treatment of ...... | 13 | 564 |
| Hysteria, Dr. Bright on........... | 16 | 323 |
| Hysteria aping various affections.... | 16 | 324 |
| Hysteria, Dr. Graves on .......... | 19 | 147 |
| Hysteria, fatal, Dr. D. Davis on .... | 19 | 190 |
| Hysteric affections of the larynx.... | 6 | 419 |
| Hysterical affections of the joints. .. | 9 | 46 |

**I**

| | Vol. | Page |
|---|---|---|
| Iatraleptic treatment of dropsy ..... | 10 | 199 |
| Ice to aneurisms .................. | 8 | 576 |
| Ice to the epigastrium, for biliary calculi ..................... | 11 | 447 |
| Icterus, fatal case of.............. | 9 | 440 |
| Ictus solaris, a case of, by Mr. Brown | 5 | 290 |
| Idiopathic tetanus, cases of........ | 3 | 391 |
| Idiopathic fever, cases of.......... | 3 | 525 |
| Idiopathic carditis, description of. .. | 11 | 248 |
| Idiopathic phlebitis, case of........ | 20 | 253 |
| Idiosyncrasy of individuals, M. Martinet on ................. | 2 | 433 |
| Idiosyncrasy and temperament ..... | 13 | 2 |
| Idiotism, remarkable case of, cured.. | 3 | 557 |
| Idiotcy from nervous injury ....... | 2 | 469 |
| Idiotcy from fright ............... | 13 | 512 |
| Ileum, rupture of the ............ | 9 | 498 |
| Ileus, from calculous concretions ... | 6 | 574 |
| Ileus, Dr. Abercrombie on ........ | 11 | 3 |
| Ileus, cases of................... | 14 | 509 |
| Iliac tumor in parturition, case of .. | 3 | 416 |
| Iliac region, peculiar abscess in the.. | 9 | 274 |
| Iliac fossa, phlegmonous tumors in, | 10 | 217 |
| Iliac abscesses and tumours, M. Teallier on................... | 12 | 160 |
| Iliac aneurism—aorta tied ........ | 14 | 50 |
| Iliac artery (common) tied by Mr. Crampton .................... | 14 | 54 |
| Iliac fossa, phlegmonous tumors on.. | 16 | 225 |
| Iliac aneurism successfully treated, cases ...................... | 16 | 514 |
| Iliac, common artery, ligature of ... | 20 | 278 |
| Ilium, enormous tumor attached to, | 18 | 236 |
| ILL-HEALTH, considerations on its origin...................... | 9 | 55 |
| Illustrations of amputations ...... | 16 | 242 |
| Imagination, portrait of the........ | 13 | 291 |
| Imperforate anus, successful operation | 5 | 187 |
| Imperforate anus, Mr. Hutchison on, | 5 | 511 |
| Imperforate anus, various cases of .. | 5 | 515 |
| Imperforate rectum, case of, cured.. | 5 | 615 |
| Imperforate anus—operation—death, | 10 | 515 |
| Imperforate rectum, treatment of .. | 11 | 261 |
| Imperforate vagina—fatal operation, | 13 | 466 |
| Imperforate os uteri—operation for, | 13 | 552 |
| Imperforate anus, case of.......... | 15 | 268 |
| Imperforate vagina, operation for .. | 16 | 602 |
| Imperforate hymen, effects of... .. | 19 | 190 |
| Impetigo, M. Biett on ............ | 16 | 396 |
| Importance of accoucheurs ........ | 3 | 175 |
| *Important* works on the venereal ... | 3 | 436 |
| Improvements on the forceps, Dr. Davis's ..................... | 3 | 433 |
| Impulse of the heart.............. | 13 | 124 |
| Inanition, dissection after death from | 17 | 185 |
| Incarcerated intestine, fatal adhesion of its surfaces .......... | 4 | 231 |
| Incised wounds, Mr. Symes' treatment | 3 | 545 |

| | Vol. | Page |
|---|---|---|
| Incisions in erysipelas | 7 | 244 |
| Incisions in dissection-wound | 9 | 174 |
| Incisions in erysipelas | 10 | 236 |
| Inclined plane, its advantages considered | 3 | 373 |
| Incontinence of urine, M. Lair's remarks on | 6 | 244 |
| Incontinence of urine, cured by dry cupping | 7 | 278 |
| Incontinence of urine, cure of | 14 | 569 |
| Incurvations of the spine | 2 | 132 |
| *Independence* of organs considered | 6 | 102 |
| Indian diseases, calomel in | 3 | 576 |
| Indian diseases, Mr. Annesley on | 4 | 88 |
| Indian diseases, Mr. Annesley on | 13 | 213 |
| Indian cholera, Dr. Smith on | 14 | 347 |
| Indigestion, Mr. Howship on | 4 | 34 |
| Indigestion, a *fashionable* case of | 4 | 566 |
| Indigestion, definition and symptoms of | 6 | 27 |
| Indigestion, Dr. Philip on | 7 | 362 |
| Indigestion, Mr. Mayo on | 15 | 41 |
| Indo-Russian cholera | 15 | 447 |
| Induration of the cellular membrane, | 6 | 565 |
| Induration, general, of the arterial system | 10 | 174 |
| Infamy of the Lancet | 11 | 153 |
| Infant, amputation in an | 20 | 191 |
| Infanticidal monomania, dreadful instance of | 9 | 441 |
| Infanticide, various authors on | 1 | 337 |
| Infanticide, alledged case of | 5 | 239 |
| Infanticide, new tests for the detection of | 5 | 297 |
| Infanticide, question of insanity in | 5 | 617 |
| Infantile erysipelas, quinine in | 1 | 224 |
| Infants, ophthalmia of, Mr. Ryall | 2 | 220 |
| Infants, newly-born, when they should be put to the breast | 3 | 488 |
| Infants, their natural language | 5 | 150 |
| Infants, diseases of the | 9 | 234 |
| Infants, diseases of | 19 | 361 |
| Infants, eclampsia in | 20 | 240 |
| Infection, remarks on | 3 | 83 |
| Inferior cava, obliteration of | 13 | 550 |
| Infiltration of urine, in lithotomy, prevented | 3 | 248 |
| Infirmaries and dispensaries | 10 | 544 |
| Inflamed absorbents, caustic for | 6 | 246 |
| Inflammation, Dr. Armstrong on | 1 | 118 |
| Inflammation of the semilunar ganglia | 1 | 187 |
| Inflammation of veins—Dr. Duncan, | 2 | 161 |
| Inflammation of nerves—M. Martinet | 2 | 165 |
| Inflammation of appendix cœci | 2 | 200 |
| Inflammation, hæmorrhoidal | 2 | 280 |
| Inflammation, Dr. Uwins' remarks on | 2 | 387 |
| Inflammation of veins, Dr. Chapman's case of | 2 | 447 |
| Inflammation of veins, Mr. Hodgson's case of | 2 | 449 |
| Inflammation and irritation of the bronchiæ | 2 | 451 |

| | Vol. | Page |
|---|---|---|
| Inflammation and fever not identical | 3 | 88 |
| Inflammation, tracheal & laryngeal | 3 | 347 |
| Inflammation of the retina, anglicè, iritis | 3 | 362 |
| Inflammation & reddening considered | 3 | 496 |
| Inflammation of the lungs, its progress | 3 | 510 |
| Inflammation, gouty, how treated | 4 | 51 |
| Inflammation, abdominal, in irritative fever | 4 | 74 |
| Inflammation, irritable, Sir A. Cooper's definition of | 4 | 110 |
| Inflammation of the membranes of the brain | 4 | 187 |
| Inflammation of the liver, &c. remarkable case of | 4 | 520 |
| Inflammation from injuries and operations | 5 | 121 |
| Inflammation of the blood-vessels, cases of | 6 | 501 |
| Inflammation of the spinal marrow | 7 | 464 |
| Inflammation of the prepuce, with phymosis | 7 | 526 |
| Inflammation by contiguity | 8 | 505 |
| Inflammation of the placenta | 9 | 194 |
| Inflammation, effects of calomel and opium in | 9 | 238 |
| Inflammation; influence of the nervous system | 10 | 198 |
| Inflammation of an ovarian cyst | 11 | 258 |
| Inflammation of the veins | 12 | 1 |
| Inflammation of the veins, secondary affection in | 12 | 19 |
| Inflammation of the spleen, cases of, | 12 | 252 |
| Inflammation of the peritoneum | 12 | 313 |
| Inflammation and fever | 12 | 344 |
| Inflammation of the ovarium | 12 | 417 |
| Inflammation simulated by hysteria, | 13 | 32 |
| Inflammation of the pericardium | 13 | 183 |
| Inflammation of the choroid | 13 | 253 |
| Inflammation, on the loss of blood in, | 13 | 325 |
| Inflammation, simulated by irritation | 13 | 331 |
| Inflammation, acute, of testis | 13 | 388 |
| Inflammation of the tunica vaginalis, | 14 | 23 |
| Inflammation of brain, from old injury | 14 | 231 |
| Inflammation, theory of | 14 | 393 |
| Inflammation of the lens | 15 | 71 |
| Inflammation of uterus, Dr. Lee on | 15 | 425 |
| Inflammation, Mr. James on | 17 | 411 |
| Inflammation, theory of, considered, | 17 | 411 |
| Inflammation from various poisons | 17 | 419 |
| Inflammation of the brain, Mr. Crampton on | 18 | 237 |
| Inflammation, granular, of os tincæ, | 19 | 524 |
| Inflammation, Mr. Rogerson on | 20 | 320 |
| Inflammation, a general cause of disease | 20 | 321 |
| Inflammation, divisions of | 20 | 322 |
| Inflammation, Dr. Phillips on | 20 | 324 |
| Inflammation, theory of | 20 | 324 |
| Inflammations simulated by hysteria, | 12 | 186 |
| Inflammations, secondary, M. Velpeau on | 18 | 398 |

| | Vol. | Page |
|---|---|---|
| Inflammations, secondary, history of, | 18 | 399 |
| Inflammations, secondary, treatment, | 18 | 405 |
| Inflammatory form of yellow fever, Dr. Wilson on | 8 | 75 |
| Inflammatory origin of tubercles.... | 10 | 74 |
| Inflammatory asthma! | 11 | 517 |
| Inflation for colic | 15 | 157 |
| Inflation of the bowels, in ileus, &c.. | 15 | 267 |
| Influence of climate on chronic diseases, Dr. Clark on | 11 | 121 |
| Influenza, surmise on a cause of it.. | 3 | 449 |
| Influenza, epidemic | 18 | 154 |
| Influenza at Berlin | 19 | 484 |
| Influenza, Dr. Ward on the | 20 | 364 |
| Ingleby, Mr. on uterine hæmorrhage, | 17 | 214 |
| Inguinal hernia, strangulated, case of, | 6 | 609 |
| Inguinal aneurism, Mr. Brodie's case, | 8 | 547 |
| Inguinal hernia, new species of .... | 12 | 180 |
| Inguinal hernia, remarks on | 12 | 450 |
| Inhalation of chlorine in consumption | 14 | 103 |
| Inhalation in phthisis, Sir C. Scudamore on | 14 | 352 |
| Inhalation of iodine, &c. in phthisis, | 20 | 420 |
| Injection of tartar-emetic into veins, | 1 | 484 |
| Injection of the meatus auditorius .. | 3 | 112 |
| Injection of a psoas abscess | 10 | 153 |
| Injection, successful, of ovarian tumor | 15 | 501 |
| Injection of encysted dropsy, death from | 17 | 483 |
| Injections employed in France | 3 | 352 |
| Injuries of the head, insidious nature of | 4 | 242 |
| Injuries of the head, Mr. Jeffreys on, | 5 | 599 |
| Injuries of the thorax, Messrs. Bell and Shaw on | 5 | 625 |
| Injuries of the head, M. Paillard on, | 6 | 169 |
| Injuries of the head, cases, with remarks | 6 | 263 |
| Injuries of the head, cases of | 6 | 529 |
| Injuries of the head, further cases of, | 6 | 541 |
| Injuries of the head, cases of | 9 | 186 |
| Injuries of the brain, Mr. Brodie on, | 9 | 400 |
| Injuries of the head, hospital report on | 9 | 444 |
| Injuries of the spine, hospital report on | 9 | 449 |
| Injuries of the head | 11 | 232 |
| Injuries of the head, cases of | 11 | 238 |
| Injuries of the head, cases of | 11 | 283 |
| Injuries of the head | 12 | 28 |
| Injuries of arteries, Mr. Guthrie on, | 13 | 133 |
| Injuries of the head, report on | 13 | 233 |
| Injuries of the head | 13 | 557 |
| Injuries of the head, hospital report on | 14 | 274 |
| Injuries of head, report on | 15 | 505 |
| Injuries of the head, treatise on | 15 | 532 |
| Injuries of the head, Mr. Crampton on | 17 | 507 |
| Injuries, cause of death after | 19 | 195 |
| Injuries of the head, Mr. B. Cooper on, | 20 | 556 |
| Injury to the spinal column | 3 | 225 |
| Injury of the head—trephine applied, | 8 | 521 |
| Injury, fatal, of the knee-joint | 12 | 230 |
| Injury of the spine, cases of | 14 | 226 |
| Injury of the lungs—recovery | 15 | 270 |
| Injury of the spine—partial paralysis | 15 | 274 |
| Inoculation of syphilitic virus | 1 | 199 |
| Inorganic bodies, the analysis of.... | 19 | 337 |
| Inquest on St. John Long | 13 | 520 |
| Inquests, abuses of | 3 | 173 |
| Insane, illusions of the | 19 | 352 |
| Insane, confinement of the | 19 | 356 |
| Insane, otitis in the | 20 | 201 |
| Insanity, Dr. Willis on | 2 | 360 |
| Insanity, not always elucidated by dissection | 3 | 231 |
| Insanity, idiopathic and symptomatic | 5 | 226 |
| Insanity, pathology of | 7 | 17 |
| Insanity, the work of " a Keeper" on, | 9 | 29 |
| Insanity, incipient, the treatment of, | 9 | 31 |
| Insanity, discussion on | 9 | 190 |
| Insanity, Dr. Burrows on | 10 | 1 |
| Insanity, moral causes of | 10 | 3 |
| Insanity, physical causes of | 10 | 6 |
| Insanity, hereditary predisposition to | 10 | 11 |
| Insanity, divisions of | 10 | 11 |
| Insanity, characters, or symptoms of, | 10 | 13 |
| Insanity, seclusion in | 10 | 17 |
| Insanity, moral treatment of | 10 | 18 |
| Insanity, medical treatment of | 10 | 19 |
| Insanity—trial of Mr. Davies | 12 | 433 |
| Insanity, Dr. Conolly on | 13 | 289 |
| Insanity, theory of | 14 | 304 |
| Insanity and paralysis—dissection .. | 15 | 186 |
| Insanity, Sir H. Halford on | 15 | 361 |
| Insanity, post-mortem appearances of | 16 | 538 |
| Insanity, Dr. Seymour on | 18 | 139 |
| Insanity, treatment of | 18 | 141 |
| Insanity; post-mortem appearances, | 18 | 502 |
| Insanity in France | 18 | 531 |
| Insanity in Italy | 19 | 203 |
| Insanity, curability of, Dr. Uwins on, | 20 | 56 |
| Insects discharged from the stomach—case of | 1 | 367 |
| Insensibility of the retina | 4 | 303 |
| Insipidity of the bile, remarks on.... | 7 | 367 |
| Instinct, as compared with intellect, | 6 | 318 |
| Institute of France, proceedings at.. | 18 | 540 |
| Institute, French, proceedings of.... | 19 | 230, 502 |
| Institute, French, proceedings of.... | 20 | 231 |
| Institutions, medical, of this country | 15 | 162 |
| Instrument of justice, sample of the, | 8 | 214 |
| Instrument for lateral curvature.... | 15 | 420 |
| Instruments, parturient, Dr. Davis's, | 3 | 406 |
| Instruments in twin-births | 3 | 417 |
| Instruments, when indicated in labour | 3 | 424 |
| Insurance of lives—medical evidence, | 10 | 213 |
| Intellect and disease, march of | 12 | 300 |
| Intellect, march of the | 19 | 184 |
| Intellectual labour, deplorable effects of | 12 | 302 |
| Intellectual powers, Dr. Abercrombie on the | 14 | 289 |
| Intelligence, &c. | 3 | 313 |

| | Vol. | Page |
|---|---|---|
| Intemperance, melancholy consequences of; a case | 5 | 361 |
| Intemperance as a disease, Dr. Kain on | 10 | 193 |
| Intemperance, powerful remarks on, | 13 | 87 |
| Intermissions of the pulse, on what depending | 10 | 562 |
| Intermittent, partial | 2 | 201 |
| Intermittent affection of the chest, case of | 3 | 272 |
| Intermittent irritations, M. Dufau on, | 6 | 457 |
| Intermittent arachnitis, supposed cases of | 6 | 460 |
| Intermittent fever, cases of | 6 | 606 |
| Intermittent fever, modes of cure ... | 6 | 606 |
| Intermittent inflammation | 7 | 60 |
| Intermittent rheumatic fever | 7 | 171 |
| Intermittent nasal hæmorrhage | 7 | 196 |
| Intermittent fever, Dr. Macculloch on | 9 | 291 |
| Intermittent fever, treatment of | 9 | 302 |
| Intermittent dyspnœa | 10 | 532 |
| Intermittent, malignant, case of | 11 | 278 |
| Intermittent aphonia, cases of | 11 | 488 |
| Intermittent ophthalmia | 12 | 534 |
| Intermittent gastro-enteralgia | 14 | 468 |
| Intermittent hemiplegia | 14 | 514 |
| Intermittent affections, cases of | 15 | 238 |
| Intermittent tetanus | 15 | 570 |
| Intermittents, masked | 1 | 485 |
| Intermittents denied by Dr. Mills .. | 2 | 16 |
| Intermittents, sulph. zinci in | 3 | 168 |
| Intermittents, a stumbling-block to the Broussaians | 3 | 328 |
| Intermittents, Broussais' views of .. | 3 | 332 |
| Intermittents, M. Recamier's *philosophical* treatment of | 4 | 209 |
| Intermittents, an *eye-sore* to the *exclusionists* | 5 | 11 |
| Intermittents, Broussais *v.* bark | 5 | 29 |
| Intermittents, new mode of treating, | 6 | 229 |
| Intermittents; tartar-emetic in . .. | 6 | 531 |
| Intermittents, treatment of | 13 | 154 |
| Internal abscess, an interesting case, | 4 | 286 |
| Internal strangulation | 6 | 272 |
| Internal strangulation, M. Louis's cases of | 8 | 129 |
| Internal iliac artery, ligature of the.. | 9 | 232 |
| Internal strangulation, remarkable case of | 11 | 247 |
| Internal strangulations | 13 | 544 |
| Internal strangulations | 13 | 568 |
| Internal iliac; dissection of Dr. Stevens' case | 14 | 57 |
| Interstitial absorption of bone | 10 | 413 |
| Intestinal tympanitis, operation for.. | 1 | 220 |
| Intestinal calculi, Mr. Torbet's case of | 3 | 532 |
| Intestinal calculi, causes of | 3 | 533 |
| Intestinal irritation, its symptoms .. | 4 | 343 |
| Intestinal ulcers, cicatrization of | 5 | 192 |
| Intestinal tube, spasm of the, case .. | 5 | 504 |
| Intestinal ulceration after fever, Dr. Johnson's case of | 6 | 497 |
| Intestinal invaginations, M. Buet on, | 8 | 154 |
| Intestinal inflammation, terminations of | 11 | 24 |
| Intestinal irritation, Dr. Hall on | 11 | 291 |
| Intestinal irritation, cases of | 11 | 293 |
| Intestinal irritation, treatment of ... | 11 | 296 |
| Intestinal glands, state of, in cholera, | 20 | 67 |
| Intestinal tube, state of, in cholera.. | 20 | 76 |
| Intestine small, loss of upwards of four feet of | 4 | 30 |
| Intestine, wounded, cases of | 20 | 182 |
| Intestines, perforation of | 2 | 182 |
| Intestines, M. Paillard on wounds of, | 6 | 196 |
| Intestines, chronic diseases of the .. | 11 | 26 |
| Intestines, sudden protrusion of, into scrotum | 11 | 268 |
| Intestines, Dr. Wise on wounds of.. | 18 | 250 |
| Intestines, wounds of, B. Cooper on, | 20 | 141 |
| Intestinum ileum, perforated, case of, | 8 | 225 |
| Intoxication, asphyxia during | 20 | 196 |
| Introduction of the forceps | 3 | 429 |
| Introduction of a bougie, followed by purulent depôts | 10 | 458 |
| Intus-susceptio, a remarkable case of | 2 | 460 |
| Intus-susceptio | 11 | 105 |
| Intus-susception, operation for | 3 | 537 |
| Intus-susception, interesting case of, | 3 | 539 |
| Intus-susception, M. Dance's memoir on | 6 | 176 |
| Intus-susception, *supposed* case of .. | 6 | 177 |
| Intus-susception, diagnosis and treatment of | 6 | 185 |
| Inversio vesicæ, case of | 18 | 459 |
| Inverted toe-nail, M. Dupuytren on, | 5 | 575 |
| Inverted uterus, extirpation of | 14 | 251 |
| Iodide of mercury in lupus | 12 | 570 |
| Iodine | 1 | 55 |
| Iodine, Dr. Gairdner on | 1 | 104 |
| Iodine, memoirs on the use of | 2 | 229 |
| Iodine, dangers of | 2 | 233 |
| Iodine in venereal affections | 3 | 277 |
| Iodine in sulphureous springs | 3 | 298 |
| Iodine, Dr. Manson's researches on.. | 4 | 1 |
| Iodine, formula for a tincture of it.. | 4 | 4 |
| Iodine, its powers in a case of palsy, | 4 | 7 |
| Iodine, for enlargements of the liver and spleen | 9 | 168 |
| Iodine in chronic ulcerations of the tongue | 9 | 197 |
| Iodine in diseases of the joints | 10 | 129 |
| Iodine in enlargement of the uterus.. | 10 | 200 |
| Iodine, Dr. Baron on | 11 | 451 |
| Iodine in bronchocele, scrofula, and ascites | 12 | 248 |
| Iodine, morbid effects of | 12 | 572 |
| Iodine in leucorrhœa | 13 | 423 |
| Iodine in cutaneous diseases | 15 | 208 |
| Iodine for epilepsy | 15 | 494 |
| Iodine, M. Lugol on, in scrofula | 16 | 109 |
| Iodine, effects of | 16 | 111 |
| Iodine, M. Lugol's form of | 16 | 112 |
| Iodine, external local effects of ... Iodine, internal | 16 | 113 |

| | Vol. | Page |
|---|---|---|
| Iodine for scrofulous tubercles...... | 16 | 119 |
| Iodine for scrofulous ophthalmia.... | 16 | 120 |
| Iodine for scrofulous cutaneous tissue | 16 | 121 |
| Iodine for scrofulous cellular tissue.. | 16 | 123 |
| Iodine for scrofulous bones........ | 16 | 123 |
| Iodine, preparations of............ | 16 | 125 |
| Iodine, caustic ................ | 16 | 127 |
| Iodine, use of, in scrofula ......... | 17 | 169 |
| Iodine for secondary eruptions ..... | 17 | 263 |
| Iodine in caries of the vertebræ .... | 19 | 266 |
| Iodine against salivation ......... | 20 | 205 |
| Iodine in pseudo-syphilitic affections, | 20 | 211 |
| Iodine used in cases of tumor...... | 20 | 251 |
| Iodine used in dropsy ............. | 20 | 258 |
| Iodine to check salivation ......... | 20 | 455 |
| Iodine, remarks on its effects ...... | 20 | 467 |
| Ioduret of lead, effects of.......... | 16 | 127 |
| Ioduret of mercury .............. | 16 | 129 |
| Iodurettedbaths in scrofula......... | 16 | 115 |
| Ionian Islands, medical topography of | 14 | 97 |
| Ipecacuan emetics in mænorrhagia .. | 9 | 178 |
| Ipecacuan, large doses of, in dysentery | 14 | 332 |
| Iris, absorption of the ............ | 15 | 158 |
| Irish College of Physicians ...... .. | 8 | 490 |
| Irish Apothecaries' Company, injustice of the............... | 11 | 196 |
| Irish College of Surgeons, curriculum of the................. | 12 | 446 |
| Irish epidemics, want not the cause, | 13 | 408 |
| Irish pharmacy, Mr. Phelan on .... | 19 | 166 |
| Iritis, Dr. Robertson on .......... | 2 | 468 |
| Iritis, Mr. Melin on ............. | 2 | 471 |
| Iritis, account of ................ | 3 | 451 |
| Iritis, mercury and antiphlogistics in, | 3 | 460 |
| Iritis, closure of the pupil in, Mr. Mackenzie's theory .......... | 4 | 239 |
| Iritis, chronic, Mr. Watson on ..... | 5 | 400 |
| Iritis, oil of turpentine in.......... | 11 | 525 |
| Iritis, oil of turpentine in ......... | 12 | 232 |
| Iritis, Mr. Middlemore on ......... | 13 | 459 |
| Iritis, syphilitic, Mr. Lawrence on .. | 14 | 116 |
| Iritis, syphilitic, symptoms of...... | 14 | 116 |
| Iritis, syphilitic, effects of......... | 14 | 120 |
| Iritis, syphilitic, in infants......... | 14 | 122 |
| Iritis, syphilitic, treatment of ...... | 14 | 123 |
| Iritis, syphilitic, turpentine in...... | 14 | 127 |
| Iritis syphilitic, cases of .......... | 14 | 128 |
| Iritis, Mr. Mackenzie on .......... | 15 | 53 |
| Iritis, rheumatic ................ | 15 | 56 |
| Iritis, pseudo-syphilitic ........... | 15 | 59 |
| Iritis, scrofulous ................ | 15 | 60 |
| Iritis, arthritic .................. | 15 | 60 |
| Iritis, cases of .................. | 17 | 278 |
| Iron, remarks on, by Mr. Phillips. .. | 2 | 358 |
| Iron nail swallowed .............. | 3 | 301 |
| Iron-filings in cases of poisoning.... | 5 | 611 |
| Iron blisters .................... | 6 | 269 |
| Iron, subcarbonate of, in tetanus ... | 11 | 440 |
| Iron, nitrate of, in diarrhœa. ...... | 17 | 214 |
| Irregular determination, causes of, &c. | 4 | 130 |
| Irritable uterus.................. | 2 | 512 |
| Irritable fever, fatal case of........ | 3 | 211 |
| Irritable ulcer, application for...... | 4 | 401 |
| Irritable uterus, Dr. Gooch on the.. | 11 | 404 |
| Irritable uterus, causes of the...... | 11 | 405 |
| Irritable uterus, treatment of the .. | 11 | 406 |
| Irritable breast, case of .......... | 12 | 524 |
| Irritable testis .................. | 13 | 397 |
| Irritable uterus, on the .......... | 19 | 162 |
| Irritable testis, on the ............ | 19 | 174 |
| Irritable brain .................. | 19 | 346 |
| Irritability, proper and sympathetic, | 5 | 97 |
| Irritability and irritation.......... | 6 | 104 |
| Irritability and respiration, connexion between ............... | 18 | 2 |
| Irritability, nervous, utility of digitalis | 20 | 58 |
| Irritation, Sir A. Cooper's views of, | 4 | 100 |
| Irritation, urethral, very remarkable case of ................ | 4 | 102 |
| Irritation, treatment of ........... | 4 | 108 |
| Irritation, direct, Mr. Travers' remarks on .................. | 5 | 110 |
| Irritation of the body, its effects on the mind ............... | 5 | 388 |
| Irritation, Mr. Earle's case of...... | 6 | 199 |
| Irritation, cerebral and spinal, Dr. Darwall on ................ | 11 | 433 |
| Irritation of the brain and spine, Dr. Palmer on .............. | 12 | 293 |
| Irritation of the uterus .......... | 13 | 40 |
| Irritation, loss of blood in ........ | 13 | 327 |
| Irritation; what is it?............ | 16 | 321 |
| Irritative fever, Messrs. Butter and Tripe on ................... | 4 | 66 |
| Irritative fever, interesting case of.. | 4 | 68 |
| Irritative fever from retained placenta ..................... | 17 | 355 |
| Irving, Mr. his character by Dr. Uwins | 20 | 55 |
| Ischiatic notch, dislocation into the.. | 10 | 238 |
| Ischiatic notch, diagnosis of dislocation into the ............. | 10 | 251 |
| Ischiatic notch, dislocation into the, | 10 | 469, 537 |
| Ischuria renalis, Dr. Brown on. .... | 10 | 254 |
| Ischuria, remarkable case of ....... | 11 | 274 |
| Ischuria renalis, case of .......... | 14 | 165 |
| Ischuria renalis, of 14 years standing, | 19 | 236 |
| Issue on the vertex capitis in epilepsy | 5 | 360 |
| Issues, on the employment of...... | 18 | 512 |
| Italian doses of medicine .......... | 1 | 221 |
| Italian remedies ................. | 2 | 530 |
| Italy, new medical doctrine........ | 6 | 626 |
| Italy, climate of................. | 11 | 136 |
| Italy, climate of ................. | 14 | 148 |
| Italy, characteristics of .......... | 14 | 459 |
| Italy, climate of ................ | 14 | 462 |
| Itch, the best remedies for ........ | 3 | 570 |
| Itch, treatment of, by chloride of lime | 19 | 215 |
| Itch, bad effects from the cure of ... | 19 | 361 |
| Itching, morbid, of the scrotum.... | 12 | 493 |

J

| | Vol. | Page |
|---|---|---|
| Jackson, Dr. his case of paracentesis thoracis................ | 3 | 542 |

| | Vol. | Page |
|---|---|---|
| Jackson, Dr. his report on fever.... | 9 | 245 |
| Jackson, Dr. on cynanche ........ | 12 | 218 |
| Jacob, Dr. on a peculiar ulcer of the eye-lids ................ | 7 | 123 |
| Jacob, Mr. on internal inflammation of the eye .............. | 9 | 223 |
| Jacobson, Dr. his anastomosis between the fifth and sympathetic, | 7 | 172 |
| Jadelot, Dr. his *pratique* .......... | 3 | 345 |
| Jadelot, M. on infantile diseases .... | 12 | 541 |
| Jahn, Dr. on the bad effects of iodine | 12 | 572 |
| James, Mr. on excision of cicatrices, | 4 | 553 |
| James, Mr. his ligature of the aorta, | 14 | 50 |
| James, Mr. on inflammation ...... | 17 | 411 |
| James, Mr. on herniary sacs ....... | 19 | 368 |
| Jameson, Mr. his report on cholera.. | 17 | 72 |
| Jarrold, Dr. on spinal diseases...... | 1 | 303 |
| Jaundice, composition of blood in .. | 18 | 490 |
| Jaundice, effects of, upon vision.... | 19 | 444 |
| Jaundice, yellow, vision in......... | 19 | 447 |
| Jaundice and cerebral affection .... | 20 | 454, 479 |
| Jaw, lower, amputation of ........ | 1 | 213 |
| Jaw, M. Delpech on cancer of the .. | 13 | 546 |
| Jaw, morbid growths from the lower | 19 | 274 |
| Jaw, upper, extirpation of ........ | 19 | 281 |
| Jaw, on fracture of the lower ...... | 20 | 469 |
| Jaws, anchylosis of .............. | 2 | 472 |
| Jaws, osteo-sarcoma of the upper and lower .................. | 19 | 261 |
| Jeffreys, Dr. on disease of the heart, | 19 | 365 |
| Jejunum, rupture of the .......... | 20 | 143 |
| Jelly from bones ................ | 18 | 543 |
| Jenner, Dr. his life, by Dr. Baron .. | 7 | 102 |
| Jenner, Dr. notices of ............ | 13 | 489 |
| Jewel, Mr. on nitrate of silver...... | 12 | 517 |
| Jewel, Mr. on leucorrhœa ......... | 13 | 416 |
| John Bull, no *leper* .............. | 4 | 447 |
| John Hunter & M. Anel compared !.. | 5 | 254 |
| John Bull, letter to, on anatomy.... | 11 | 529 |
| John St. John Long.............. | 14 | 241 |
| Johnson, Dr. his reply to Dr. Good's criticisms ............ | 4 | 394 |
| Johnson, Dr. Rawlinson, on the leech | 5 | 52 |
| Johnson, Dr. his case of pneumothorax ..................... | 10 | 478 |
| Johnson, Dr. James, his case of imperfection of the heart...... | 11 | 159 |
| Johnson, Dr. James, his case of atrophy, &c. ................ | 11 | 241 |
| Johnson, Dr. his case of phthisis.... | 13 | 529 |
| Johnson, Dr. on change of air...... | 14 | 448 |
| Johnson, Dr. his propositions on cholera .................. | 16 | 274 |
| Johnson, Dr. Hackett's letter to .... | 16 | 289 |
| Johnson, Dr. S. character... ...... | 19 | 320 |
| Johnson, Mr. H. J. on gonorrhœa .. | 19 | 549 |
| Johnson, Mr. H. J. on cases of gonorrhœa .................. | 20 | 269 |
| Johnson, Mr. H. J. on venereal sores, | 20 | 529 |
| Johnson, Dr. on the intimate union of medicine and surgery ...... | 20 | 282 |
| Johnson, Dr. on the necessity of having one faculty of medicine.. | 20 | 284 |
| Joints, their injuries from exercise.. | 3 | 381 |
| Joints, carious, cases of excision of, | 7 | 301 |
| Joints, affections of, after injuries .. | 12 | 24 |
| Joints, affections of .............. | 12 | 31 |
| Joints, affection of............... | 12 | 383 |
| Joints, Mr. Syme's excision of ..... | 13 | 273 |
| Joints, diseased, excision of........ | 15 | 113 |
| Joints, ulceration of, Mr. Key on ... | 20 | 25 |
| Jolly, Dr. on visceral neuralgia ..... | 9 | 333 |
| Jones, Mr. his remarks on dysentery, | 3 | 272 |
| Jowett, Mr. his successful case of operation for empyema ..... | 4 | 564 |
| Jowett, Mr. his case of empyema cured by paracentesis ........ | 5 | 267 |
| Jowett, Mr. taps the pericardium ... | 6 | 623 |
| July, 1830, M. Larrey on the wounded of .............. | 16 | 49 |
| July, 1830, kinds of wounds received in ................... | 16 | 50 |
| Jurisprudence, medical............. | 20 | 120 |
| Jurors, female, ignorance of........ | 20 | 122 |
| Jury, its powers on medical investigations .................. | 3 | 585 |
| Jussieu's natural classification...... | 17 | 398 |
| **K** | | |
| Karrait snake, bite from .......... | 17 | 525 |
| Kay, Dr. his experiments on tubercles | 10 | 81 |
| Kellie, Dr. on the brain............ | 1 | 285 |
| Kellie, Dr. on a peculiar spasm in children ..................... | 2 | 454 |
| Keloide, M. Biett on............... | 17 | 382 |
| Kennedy, Dr. on the management of children................. | 3 | 485 |
| Kennedy, Dr. his case of intestinal concretion .................. | 3 | 533 |
| Kennedy, Dr. on blisters .......... | 10 | 564 |
| Kennedy, Dr. on life and mind..... | 13 | 145 |
| Kennedy, Mr. his impartiality...... | 16 | 180 |
| Kennedy, Dr. on obstetric auscultation.................... | 20 | 92 |
| Kent man-of-war, fever in ........ | 2 | 10 |
| Keratonyxis for cataract .......... | 7 | 208 |
| Kermes mineral in pneumonia...... | 19 | 216 |
| Key, Mr. Aston, on lithotomy...... | 1 | 206 |
| Key, Mr. Aston, his case of ligature of the subclavian ............ | 4 | 319 |
| Key, Mr. his operation on Hoo Loo.. | 15 | 150 |
| Key, Mr. on ulceration of the joints, | 20 | 25 |
| Key's, Mr. plan of operating in hernia | 20 | 80 |
| Key, Mr. on dangers of dividing the sac ...................... | 20 | 81 |
| Kidney, state of, in dropsical diseases, | 8 | 94 |
| Kidney, varieties of diseased structure of the ................. | 8 | 99 |
| Kidney, cancer and abscess of the .. | 12 | 81 |
| Kidney, ruptured, case of.. ....... | 15 | 281 |
| Kidney, ruptured, case of.......... | 16 | 594 |
| Kidney, abscess of............... | 19 | 159, 234 |

